# A Review of Allergen Characteristics from Major Plant Allergenic Foods

**DOI:** 10.3390/foods15152600

**Published:** 2026-07-24

**Authors:** Xu Zhou, Jinshen Chu, Wenjing Du, Haochen Ye, Chen Wang, Xiaowen Pi

**Affiliations:** 1 College of Food Science, Southwest University, Chongqing 400715, China; 2 JiuJiang City Key Laboratory of Cell Therapy, Jiujiang No. 1 People’s Hospital, Jiujiang 332000, China

**Keywords:** plant food allergens, structure, epitopes, characteristics

## Abstract

With the increasing consumption of plant-based foods, allergies to these foods are becoming more widespread and seriously affect human health. This review focuses on the properties of major plant-based food allergens, including those from soybeans, peanuts, wheat, sesame, and tree nuts. The composition, stability, structure, and IgE-binding epitopes of allergens from these major plant allergenic foods are summarized. For peanuts, the major allergens include Ara h 1, Ara h 2, Ara h 3, and Ara h 6, with Ara h 2 and Ara h 6 being notably heat- and protease-resistant. Gly m 4, Gly m 5, and Gly m 6 are the predominant allergens in soybeans. The primary sesame allergens are Ses i 1, Ses i 2, Ses i 3, Ses i 6, and Ses i 7, with Ses i 1 serving as a stable and highly specific diagnostic marker. Wheat allergens are categorized by exposure route, with ingestion of allergens such as gliadins and glutenins being particularly relevant for food-induced reactions. Major storage proteins and lipid transfer proteins are central to the allergenicity of nut varieties (such as almonds, hazelnuts, and walnuts). Understanding these molecular characteristics is crucial for developing hypoallergenic food products using targeted processing methods. Further studies are needed to comprehensively investigate these allergenic characteristics.

## 1. Introduction

Allergic diseases triggered by allergens (allergenic proteins) reflect an aberrant immune response and are the sixth most widespread chronic illness worldwide [1]. Food allergies are immune system responses to specific food items. They commonly manifest as type I hypersensitivity, mediated by immunoglobulin E (IgE), which may lead to anaphylactic shock and death in severe cases [2]. Food allergies are believed to affect approximately 10% of the global population [3], with children accounting for 6–8% of cases and adults for 3–4% [2], making them a significant health concern.

While avoiding allergenic foods remains the gold standard for preventing allergic reactions in sensitive individuals, this approach can compromise balanced nutrition, potentially leading to various nutritional deficiencies, particularly in children. Moreover, allergen avoidance is often difficult in practice because it can be laborious and, in some situations, impossible. In addition, medical interventions such as antihistamines, epinephrine, and both steroidal and non-steroidal medications have their own limitations. For example, some are not suitable for children under two years of age, whereas others may impose a substantial economic burden on patients with limited financial resources. Furthermore, certain treatments, such as steroids, may cause adverse long-term side effects [4]. Thus, food processing to decrease the allergenic potential may help avert allergic responses. Before developing processing methods to reduce food allergenicity, it is critical to identify key allergen characteristics, such as composition, stability, structure, and epitopes, as these are prerequisites for the development of hypoallergenic food technologies.

Over 90% of food allergies are attributed to the so-called “nine major allergenic foods,” which include soybeans, peanuts, wheat, tree nuts, sesame, milk, eggs, shrimp, and fish [5,6]. With the increasing consumption of plant-based foods, allergies to these foods are becoming more prevalent and widespread. It has been estimated that major plant allergenic foods, namely soybean, peanut, wheat, tree nut, and sesame, cause allergic reactions in 0.5% [7], 2.2% [8], 0.2–1.0% [9], 4.9% [10], and 0.1–0.2% [11] of the global population, respectively. However, existing studies mostly focus on individual allergens or a single plant food, and relevant data are fragmented. The systematic collation and comparative analysis of major plant food allergens across categories remain insufficient. This review systematically summarizes the characteristics of the dominant allergens in soybean, peanut, wheat, sesame, and tree nuts, based on the published literature and the official database of the World Health Organization and the International Union of Immunological Societies (WHO/IUIS). This study integrates scattered research data and provides theoretical support for component-resolved clinical diagnosis, allergen detection, and the development of novel hypoallergenic processing technologies.

## 2. Literature Retrieval and Data Collection

This review followed the standard systematic review procedures for literature retrieval, screening, and data extraction. A comprehensive literature search was performed in four databases, namely PubMed, Web of Science, ScienceDirect, and Scopus, together with the official WHO/IUIS Allergen Nomenclature Database (http://www.allergen.org). The search terms included, but were not limited to, plant food allergen, peanut allergen, soybean allergen, wheat allergen, sesame allergen, and tree nut allergen, as well as allergen-characteristic terms such as structure, epitope, IgE-binding, stability, 2S albumin, 7S globulin, 11S globulin, lipid transfer protein, and prolamin. Both subject and free-text terms were combined to broaden the search scope. The inclusion criteria were as follows: (1) peer-reviewed original research articles and comprehensive reviews focusing on allergens of peanut, soybean, wheat, sesame, and tree nuts; (2) studies reporting original data on allergen composition, molecular structure, stability (thermal, enzymatic, or acid), or linear IgE-binding epitopes; and (3) authoritative data from the WHO/IUIS Allergen Nomenclature Database. Allergen characterization data were extracted into a structured table according to categories, including biochemical name, molecular weight, secondary/tertiary structure information, frequency of IgE recognition among allergic patients, isoforms/variants, and IgE-binding epitope sequences. Epitope data were cross-validated against multiple sources where available and were included only when the epitope identification method (e.g., peptide microarray, SPOT synthesis, or phage display) was clearly described in the original study. All allergen designations and nomenclature were verified against the WHO/IUIS Allergen Nomenclature Database (www.allergen.org).

## 3. Characteristics of Plant Food Allergens

### 3.1. Peanut Allergens

To date, the WHO/IUIS Allergen Nomenclature Sub-Committee has identified 16 peanut allergens (Table 1). Ara h 1 and Ara h 3 belong to the globulin family, whereas Ara h 2, Ara h 6, and Ara h 7 belong to the albumin family. Notably, most of these allergens belong to relatively minor protein families, including non-specific lipid transfer proteins (Ara h 9, Ara h 16, and Ara h 17), oleosins (Ara h 10, Ara h 11, Ara h 14, and Ara h 15), defensins (Ara h 12 and Ara h 13), profilin (Ara h 5), and pathogenesis-related proteins (Ara h 8). Apart from Ara h 1 (64 kDa) and Ara h 3 (61 and 57 kDa), the molecular masses of the remaining peanut allergens ranged from 1.2 to 17 kDa. Detection of IgE binding to Ara h 2, Ara h 6, and Ara h 7 can serve as a reliable indicator of peanut allergy [12,13].

Ara h 1, Ara h 2, Ara h 3, and Ara h 6 are the main allergens in peanuts, accounting for 12–16%, 10%, 3.7–4.3%, and 6–9% of total peanut protein, respectively [14]. Ara h 2 and Ara h 6 are more resistant to heat and proteolysis than Ara h 1 and Ara h 3, a property often attributed to their numerous disulfide bonds. Microarray immunoassay studies have shown that 95% of individuals with peanut allergy react to at least one of the three major allergens (Ara h 1, Ara h 2, and Ara h 3), and that the sera of 45% of these individuals recognize all three [15]. A biosensor-based study revealed that Ara h 1, Ara h 2, and Ara h 6 are recognized by the serum IgE of over 95% of peanut-allergic individuals [16,17]. The IgE-binding epitope sequences of these allergens are listed in Table 1. Ara h 1 can exist as monomers, trimers formed through hydrophobic interactions [18], or polymers formed through ionic bonds [19]. Several regions, residues AA25–34, 65–74, and 89–98 near the N-terminus, and residues 498–507 near the C-terminus, are immunodominant IgE-binding epitopes recognized by serum IgE in more than 80% of the patients studied [20]. The amino acids at positions 499 and 503, together with the alanine at position 502, are critical for the allergenicity of Ara h 1; mutations at these positions markedly reduce its allergenic potential [21]. Ara h 2 contains five α-helices and four disulfide bonds [22]. Epitopes 27–36, 57–66, and 65–74 have been identified as dominant [23], owing to their strong ability to bind IgE in all serum samples tested [24]. For Ara h 3, epitope 279-293 is dominant, as it is recognized by the serum IgE of all Ara h 3-reactive patients [14]. Ara h 6 consists of 127 amino acids [25] and contains five α-helices, which account for over 44% of its secondary structure and are cross-linked by five disulfide bonds [24]. Ara h 6 and Ara h 2 share 59% amino acid sequence similarity overall and 75% within their α-helical domains [26].

**Table 1:** The characteristics of peanut allergens [14,27,28].

Allergen	Biochemical Name	MW (SDS-PAGE)	Structure	Identification Rate of Allergic Patients	Isomer	Epitopes
Ara h 1	Cupin (vicillin-type, 7S globulin)	64 kDa	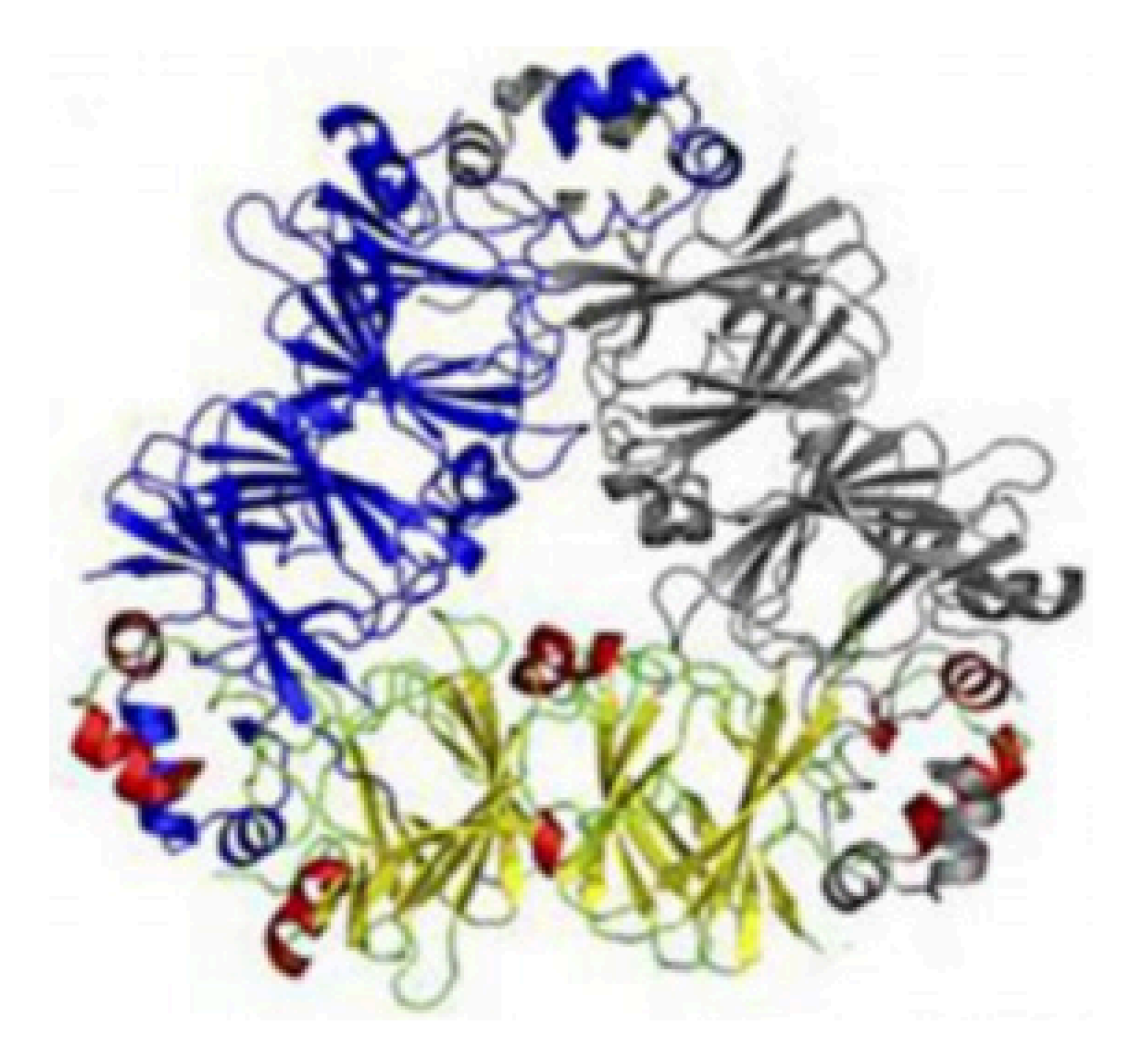	63%		AA25-34, AA48-57, AA65-74, AA89-98, AA97-105, AA107-116, AA123-132, AA134-143, AA143-152, AA294-303, AA311-320, AA325-334, AA344-353, AA361-385, AA393-402, AA409-418, AA461-470, AA498-507, AA525-534, AA539-548, AA551-560, AA559-568, AA578-587 and AA597-606
Ara h 2	Conglutin (2S albumin)	17 kDa 19 kDa	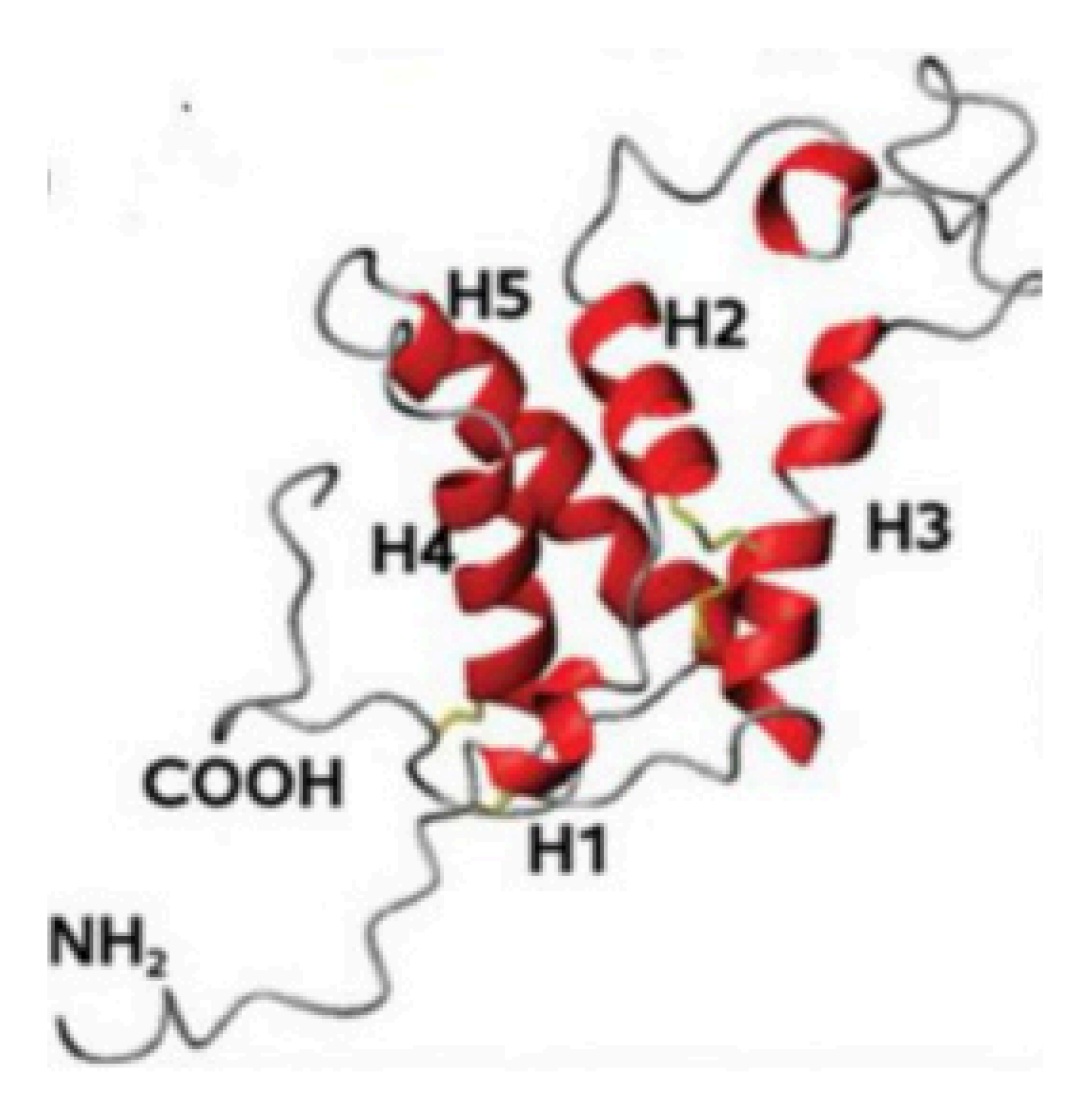	85%	Ara h 2.0101 Ara h 2.0201	AA15-24, AA21-30, AA27-36, AA39-48, AA49-58, AA57-66, AA65-74, AA115-124, AA127-136 and AA143-152
Ara h 3	Cupin (legumin-type, 11S globulin, glycinin)	58 kDa 61 kDa	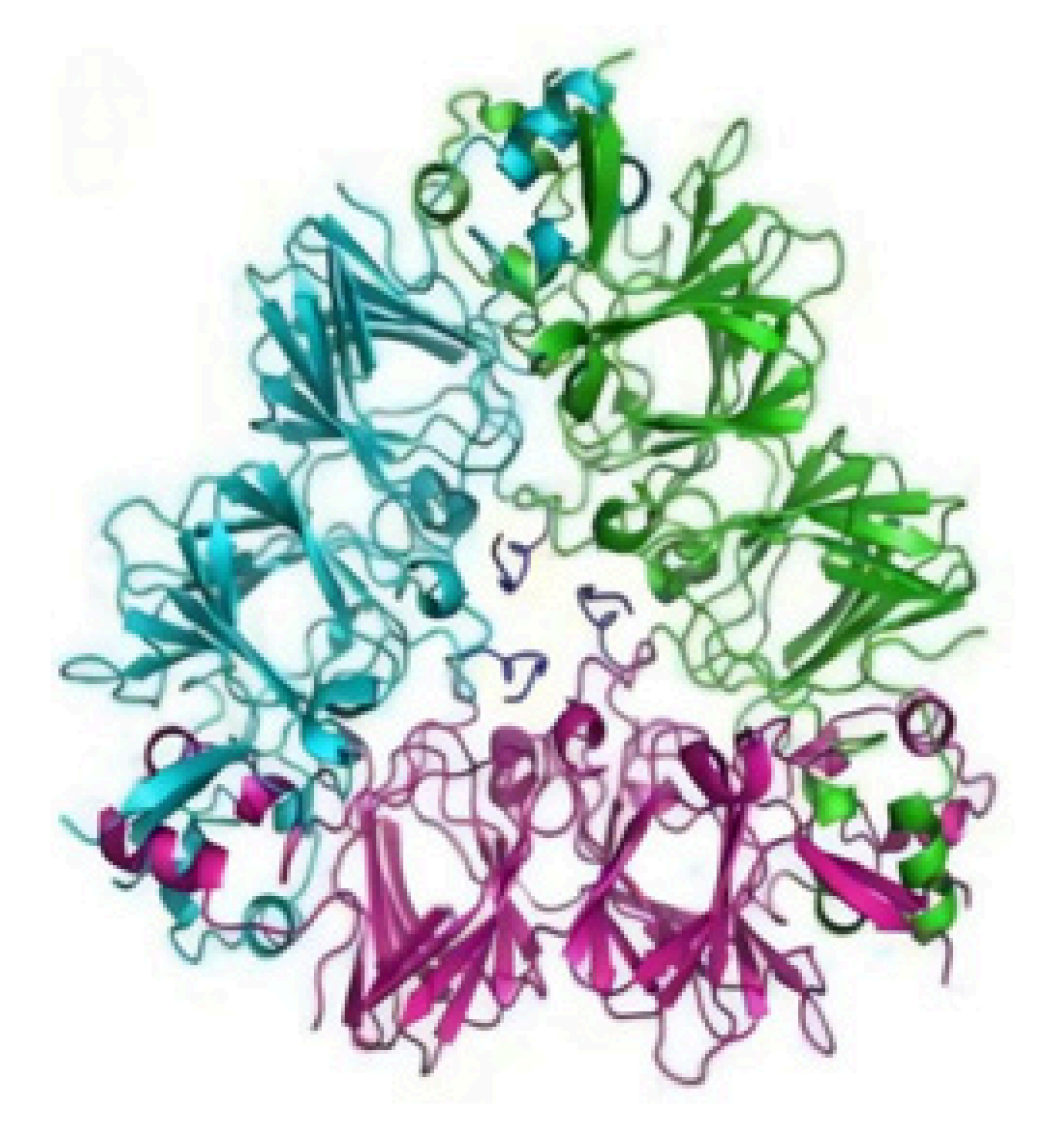	44%	Ara h 3.0101 Ara h 3.0201 (Ara h 4)	AA33-47, AA240-254, AA279-293 and AA303-317
Ara h 5	Profilin	15 kDa	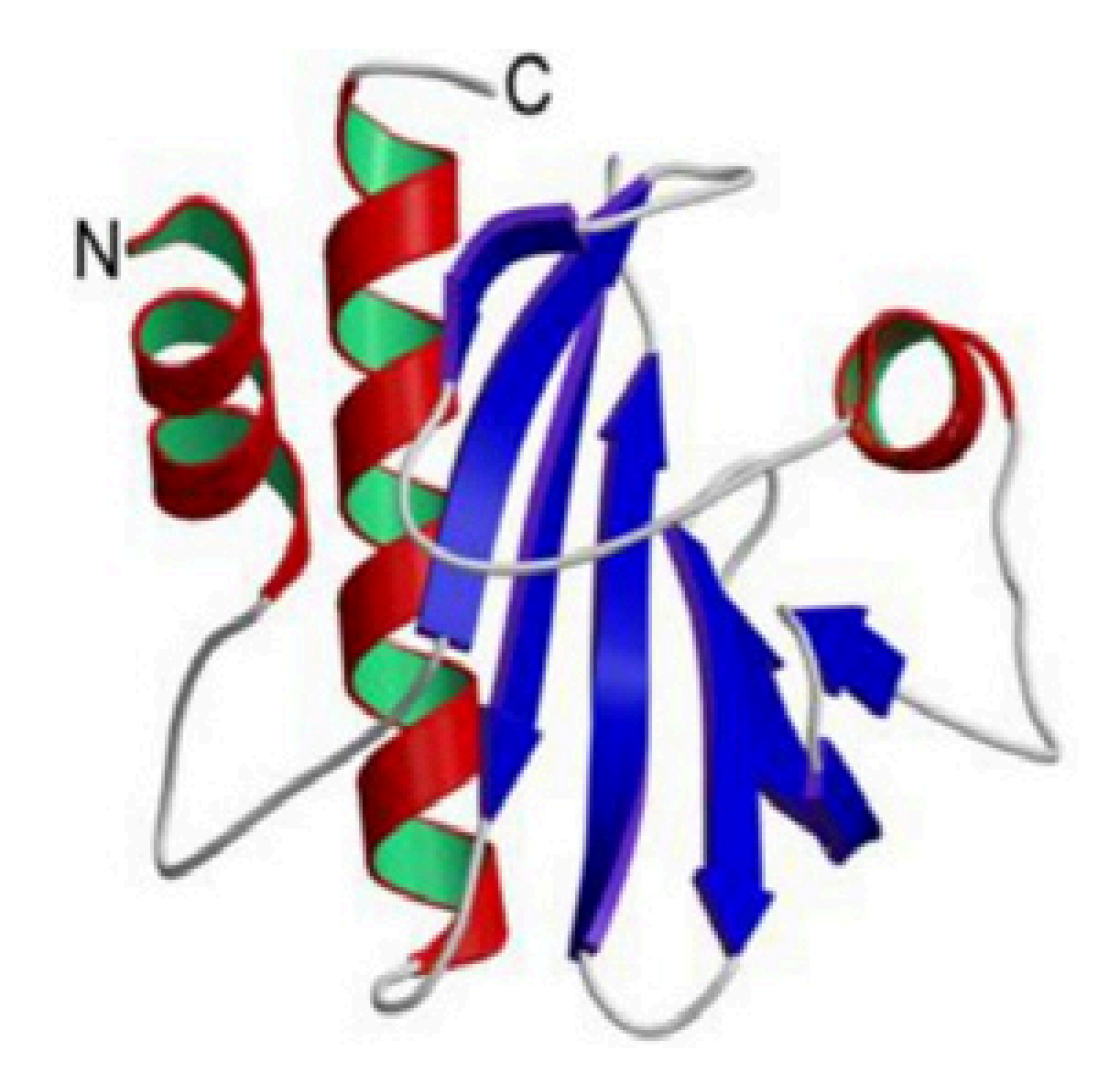	13%		
Ara h 6	Conglutin (2S albumin)	15 kDa	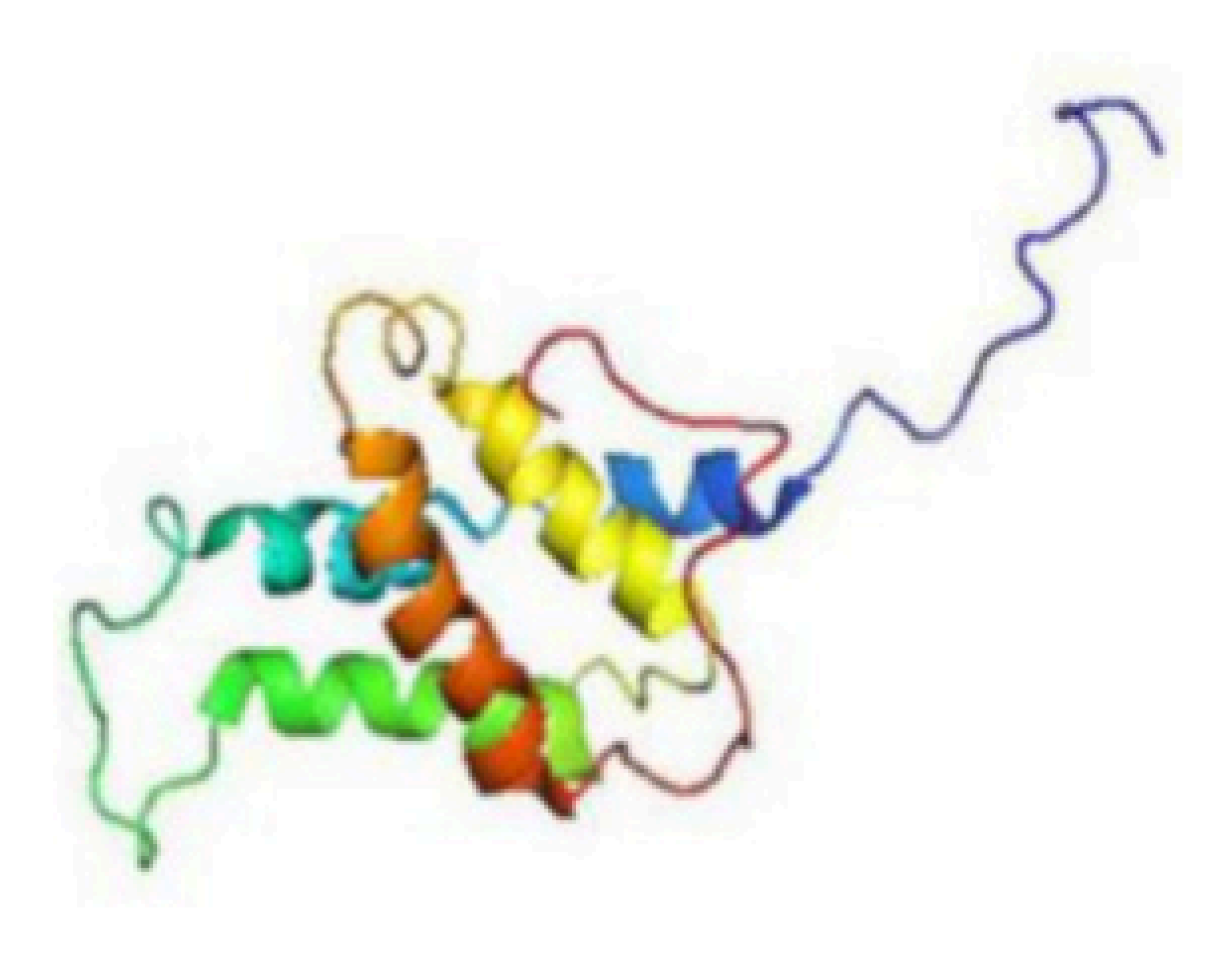	38%		AA7-25, AA41-62, AA67-74 and AA85-96
Ara h 7	Conglutin (2S albumin)	15 kDa	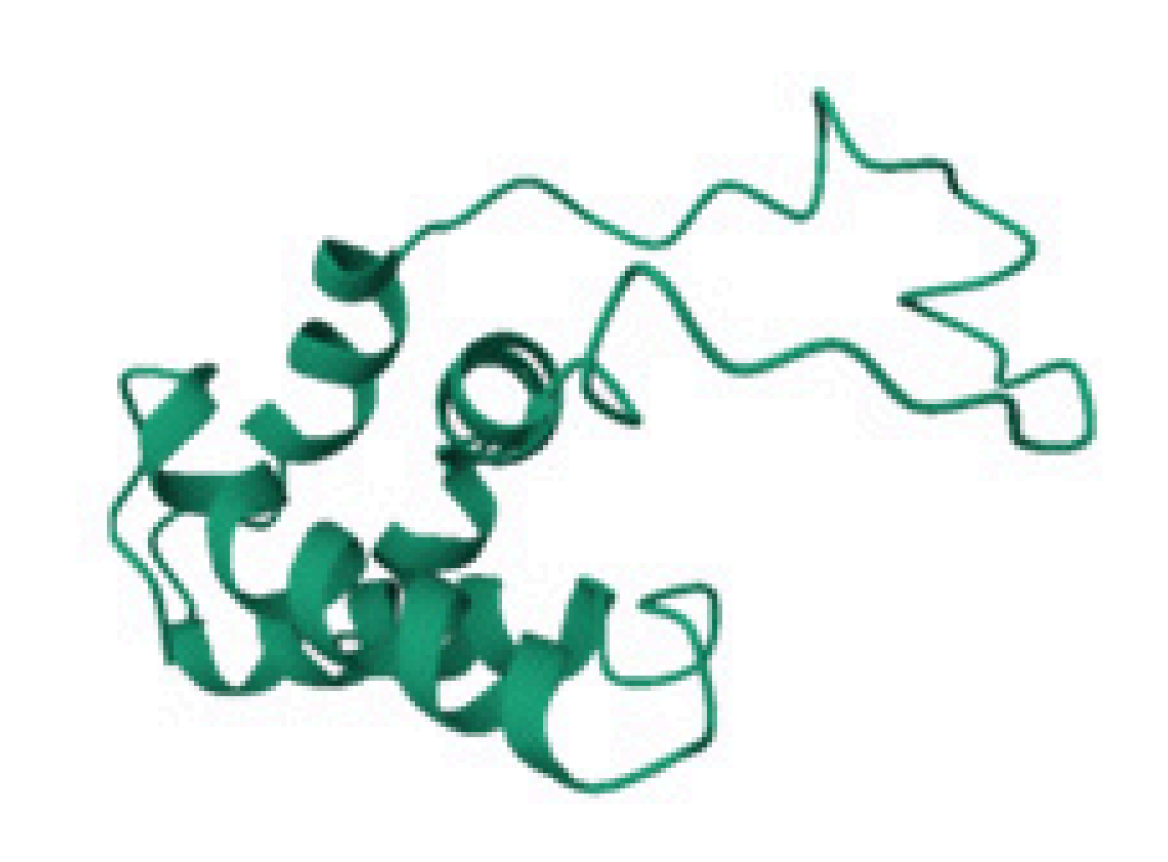	43%	Ara h 7.0101 Ara h 7.0201 Ara h 7.0301	
Ara h 8	Pathogenesis-related protein, PR-10, Bet v 1 family member	17 kDa	** 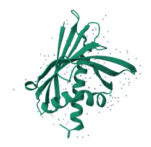 **	85%	Ara h 8.0101 Ara h 8.0201	
Ara h 9	Nonspecific lipid-transfer protein type 1	9.8 kDa	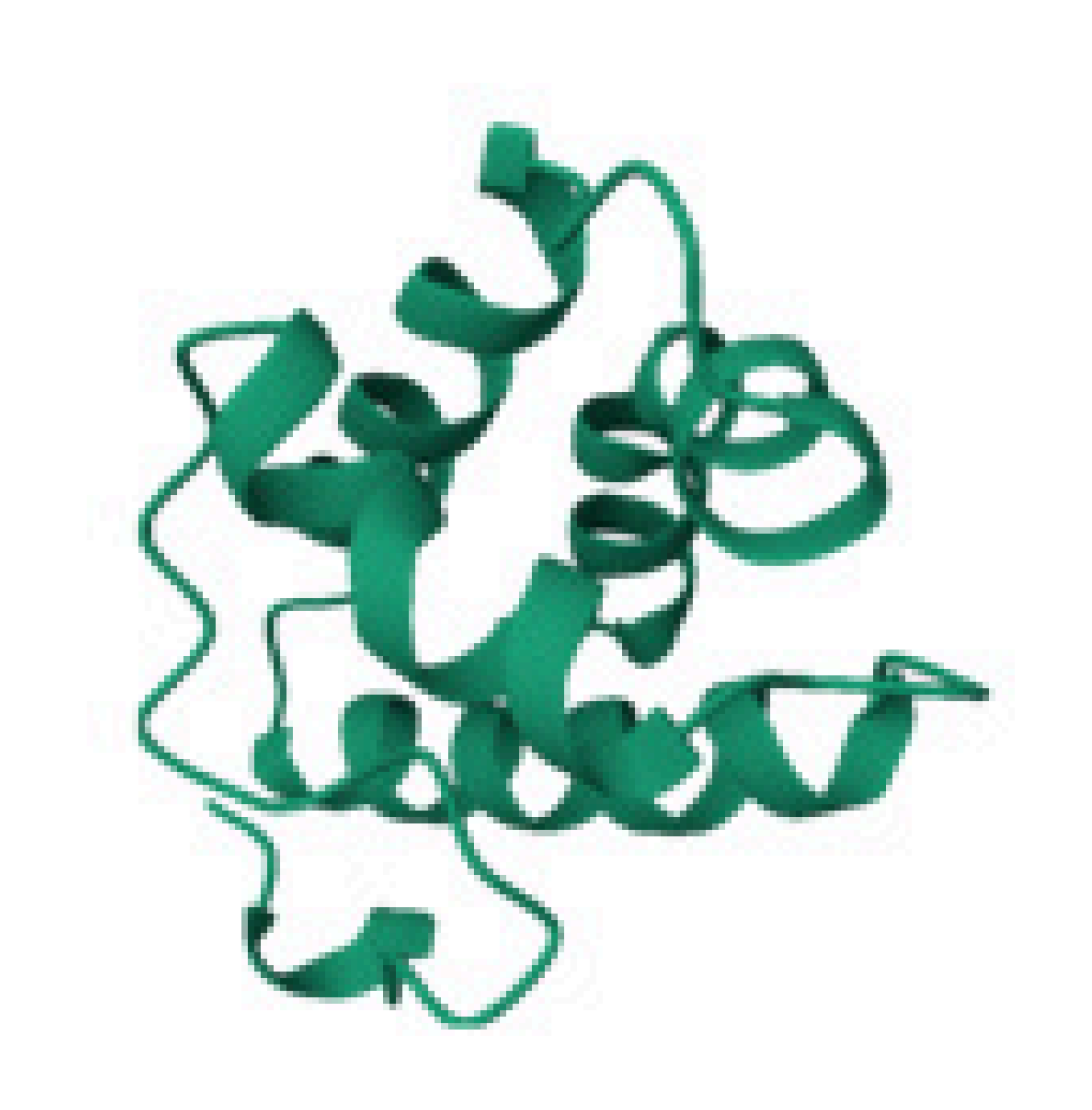	75.2%	Ara h 9.0101 Ara h 9.0201	
Ara h 10	16 kDa oleosin	16 kDa	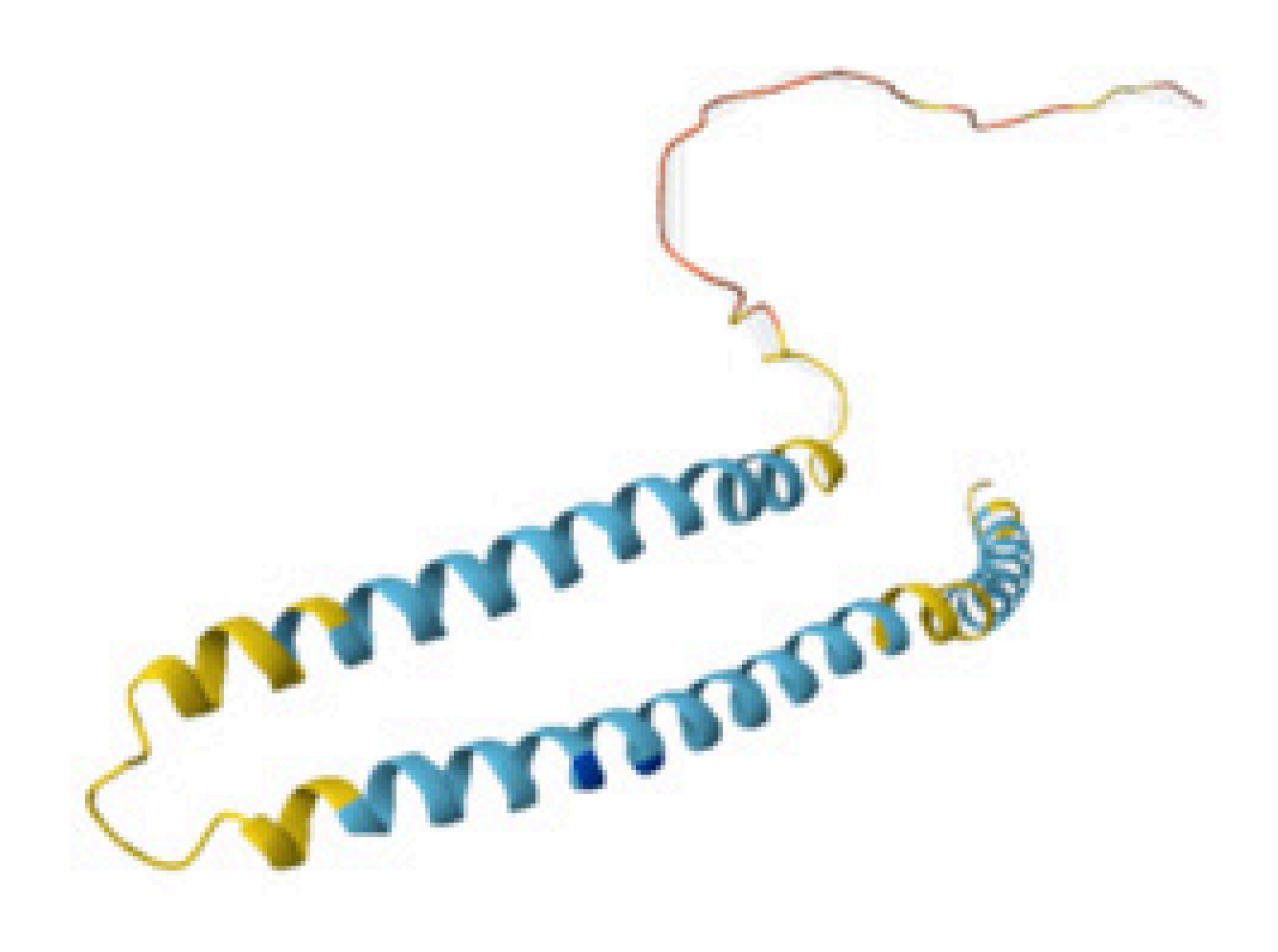		Ara h 10.0101 Ara h 10.0102	
Ara h 11	14 kDa oleosin	14 kDa			Ara h 11.0101 Ara h 11.0102	
Ara h 12	Defensin	8 kDa (reducing), 12 kDa (nonreducing), 5.184 kDa (mass)	** 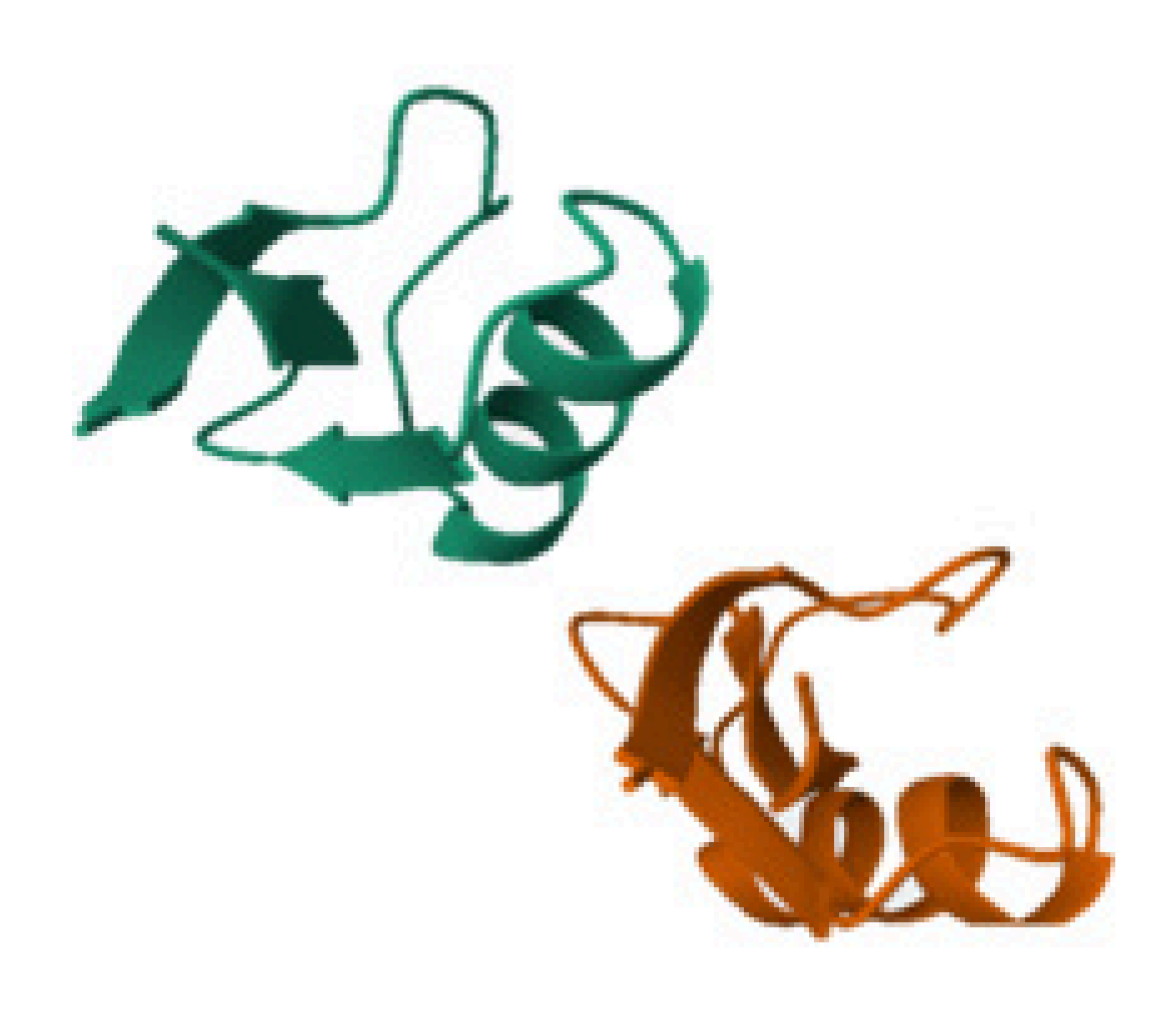 **			
Ara h 13	Defensin	8 kDa (reducing), 11 kDa (nonreducing), 5.472 kDa (mass)	** 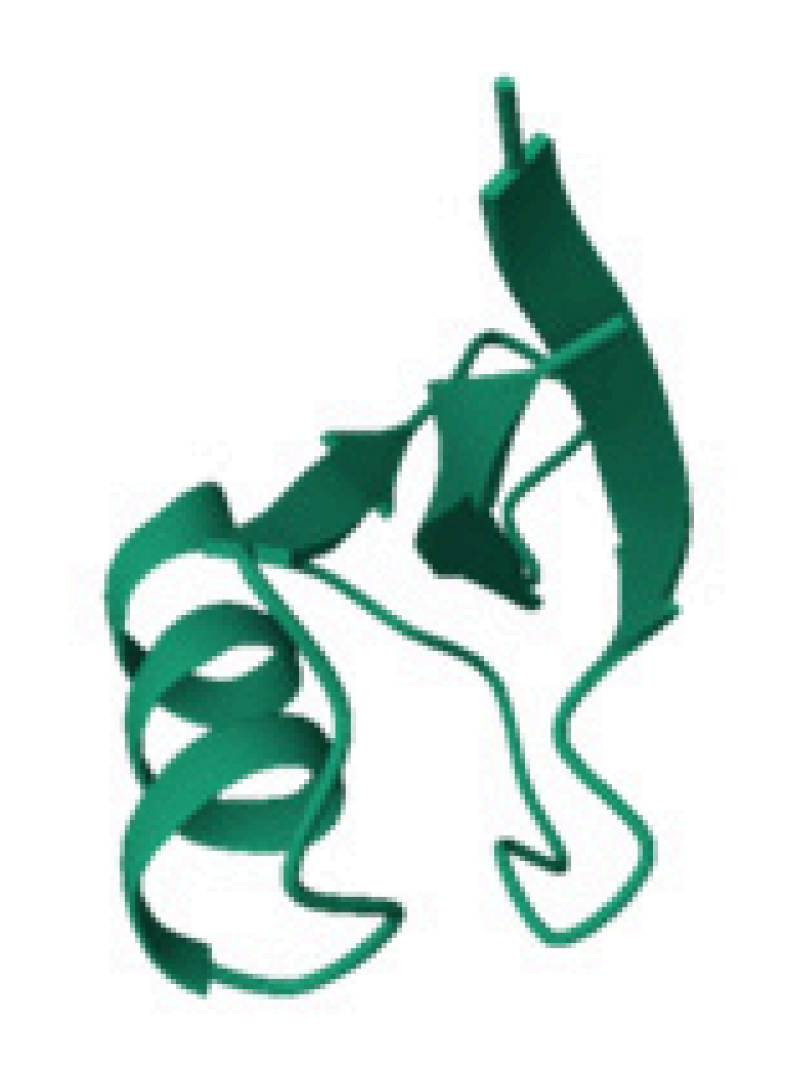 **		Ara h 13.0101 Ara h 13.0102	
Ara h 14	Oleosin	17.5 kDa		45%	Ara h 14.0101 Ara h 14.0102 Ara h 14.0103	
Ara h 15	Oleosin	17 kDa		45%		
Ara h 16	nonspecific Lipid Transfer Protein 2	8.5 kDa by SDS-PAGE reducing		16%		
Ara h 17	nonspecific Lipid Transfer Protein 1	11 kDa by SDS-PAGE reducing		16%		

### 3.2. Soybean Allergens

Eight allergenic proteins have been identified in soybeans, designated as Gly m 1 to Gly m 8. Gly m 1 and Gly m 2 are derived from soybean hulls and are classified as hull proteins [29]. Gly m 3 is a profilin, and Gly m 4 is a pathogenesis-related protein detected at 178 µg/g in soy protein isolate [30]. Gly m 5 and Gly m 6 belong to the 7S and 11S globulin fractions, respectively, which account for approximately 30% and 40% of total soybean protein [31,32]. Gly m 7 is a late embryogenesis abundant (LEA) protein, accounting for 0.01% of total seed protein [33], whereas Gly m 8 is a 2S albumin, accounting for 10% of total protein [34].

Gly m 4, Gly m 5, and Gly m 6 are the major soybean allergens [7] and can induce severe reactions in sensitized individuals [35]. Individuals sensitized to Gly m 4 often experience intense systemic reactions, particularly when they are not sensitized to Gly m 5 or Gly m 6 [35]. Children with soy allergies commonly recognize both Gly m 5 and Gly m 6 [36], with 53% of soy-allergic patients reacting to at least one of these allergens [37]. The IgE-binding epitope sequences of these allergens are summarized in Table 2. Gly m 4 is a 17 kDa water-soluble protein with limited resistance to heat, acids, and proteolytic degradation [36,38]. It consists of 157 amino acids and forms three α-helices and eight β-strands [39]. Six IgE epitopes account for over 80% of the total IgE-binding capacity of Gly m 4 [40]. A single or double amino acid substitution (N43-Q, V41-L/V44-L) reduces IgE binding by half [41]. Gly m 5 consists of three distinct subunits: the α subunit (molecular mass 57–76 kDa, isoelectric point 5.18, 10.4–20.8% of total protein), the α’ subunit (57–83 kDa, 4.90, 8.1–20.7%), and the *β* subunit (42–53 kDa, 5.66–6.00, 4.5–12.9%) [42,43]. Among these three subunits, the α subunit exhibits the most pronounced allergenicity [44]. The Gly m 5 α subunit is also known as Gly m Bd 60K, Gly m Bd 68K, or Gly m 5.0101 and shares 90.14% sequence similarity with the α’ subunit and 76.2% with the *β* subunit [45]. As shown in Table 2, 18 IgE epitopes were identified within Gly m 5.0101, of which 78.1% were located in hydrophilic regions and 68.1% on the protein surface [45]. Residues AA55-80 formed the dominant epitope, as it was recognized by the serum IgE of all study participants [46]. Gly m 6 is a hexameric protein composed of five subunit types (G1–G5), with molecular masses of approximately 56, 54, 54, 64, and 59 kDa, respectively [47,48]. Each subunit comprises a basic polypeptide chain of approximately 20 kDa and an acidic polypeptide chain of approximately 40 kDa, which are linked by a disulfide bond [49]. Acidic polypeptides are more susceptible to enzymatic hydrolysis than basic polypeptides [50]. Several acidic polypeptides significantly contribute to the allergenicity of Gly m 6 [51]. G5 was recognized by serum IgE levels of 90.9% (20 out of 22) in children with soy allergies; G1, G3, and G4 were each recognized by 16 or 17 of these children, and G2 was recognized by only 7 [48].

### 3.3. Sesame Allergens

To date, seven sesame allergens have been identified and designated as Ses i 1–7 [28]. These are summarized in Table 3. Ses i 1 and Ses i 2 are 2S albumins that together account for 15–25% of the total sesame protein; Ses i 3 is a 7S globulin constituting approximately 5%; and Ses i 6 and Ses i 7 are 11S globulins that represent 60–70% of the total sesame protein and play a central role in triggering severe allergic reactions to sesame seeds [53,54,55,56]. Ses i-1 remains stable under acidic conditions, heat treatment, and digestion by trypsin and other proteases owing to its strong aggregation through disulfide cross-linking [57,58,59]. In addition, Ses i 1-specific IgE antibodies have considerable diagnostic value for sesame allergy [60,61,62], as this marker is detectable in 100% of sesame-allergic patients (all 10 individuals tested) [63]. Although Ses i 2 is structurally similar to Ses i 1, it exhibits lower allergenicity [62]. As shown in Table 3, nine distinct epitopes have been identified for Ses i 2. Among these, epitopes AA46-55, AA48-57, and AA76-86 are the key epitopes, as they are recognized by 93.3%, 93.3%, and 93% of sesame-allergic patients, respectively [64]. Ses i 3 has a trimeric structure [65], composed of several polypeptides but lacks disulfide bonds [66]. The primary forces maintaining its polypeptide structure are hydrogen bonds, hydrophobic interactions, and electrostatic interactions [56]; consequently, Ses i-3 is prone to dissociation under alkaline conditions [56]. The key epitopes are located in β-turns and random coils (Table 3) [65]. Ses i 4 and Ses i 5 are oleosins that function as integral membrane proteins [65]. Although they represent only 1–2% of the total seed protein, they constitute approximately 80–90% of the oil-body proteins in the seed [67]. The recombinant allergens Ses i 6 and Ses i 7 were recognized by the serum IgE of 54.2% and 41.7% of sesame-allergic patients, respectively [68]. The allergen structures and IgE-binding epitope sequences of these allergens are summarized in Table 3.

### 3.4. Wheat Allergens

To date, twenty-eight wheat allergens have been identified: Tri a 12, Tri a 14, Tri a 15, Tri a 17-Tri a 21, and Tri a 25-Tri a 45 [28]. Tri a 12, Tri a 14, Tri a 15, Tri a 17, Tri a 18, Tri a 25, Tri a 27-Tri a 35, and Tri a 37-Tri a 45 are soluble in water and salt solutions, whereas Tri a 19-Tri a 21, Tri a 26, and Tri a 36 are insoluble in these solvents [70]. With respect to the exposure route, Tri a 12, Tri a 17-Tri a 20, Tri a 25, Tri a 26, Tri a 36, and Tri a 37 are associated with food allergies, whereas the other allergens typically affect the respiratory system [28]. Wheat allergens are classified into three main categories according to their route of entry into the body: ingestion (through food), inhalation (through air), and skin contact. This classification reflects the fact that wheat allergy can be triggered through multiple exposure routes, namely ingestion, inhalation, and direct skin contact [71]. Tri a 12-Tri a 20, Tri a 25, Tri a 26, and Tri a 36-Tri a 37 are classified as ingestion allergens [28] and are the primary cause of allergic reactions after the consumption of wheat-based foods. Tri a 26 is not only a food allergen but also a cutaneous allergen capable of eliciting contact urticaria. Tri a 14-Tri a 44 are primarily associated with respiratory allergies and are the main causes of baker’s asthma, a commonly recognized occupational respiratory allergy in the baking industry [72]. Individuals with baker’s asthma generally do not experience allergic reactions after the ingestion of wheat-based foods. Consequently, the effect of processing on these allergens is of limited concern, as occupational exposure results from raw wheat flour rather than processed products. Therefore, research on the effect of processing on wheat allergenicity focuses on allergens relevant to oral exposure.

The major allergenic components of wheat include non-specific lipid transfer proteins, α/β-amylase proteins, ω-5 gliadins, γ-gliadins, α/β-gliadins, high-molecular-weight glutenins, low-molecular-weight glutenins, and α-purothionin [73]. Wheat allergy encompasses IgE-mediated wheat allergy, wheat-dependent exercise-induced anaphylaxis (WDEIA), baker’s asthma, and other wheat-related disorders. All wheat allergens can cause IgE-mediated wheat allergy, whereas baker’s asthma is primarily triggered by lipid transfer proteins, non-specific lipid transfer proteins, and α-amylase/trypsin inhibitors. WDEIA is mainly induced by ω-5 gliadins or high-molecular-weight glutenins [73]. Regarding IgE-binding epitopes, Gln-Gln-Gln-Pro-Pro is the shortest primary sequence recognized in gluten, with the N-terminal glutamine residue and two proline residues playing crucial roles in binding [74]. Among the ω-gliadins, the heptapeptides QQIPQQQ, QQFPQQQ, and QQSPQQQ, along with the tetrapeptide QQQP, exhibited the strongest immunoreactivity. QQIPQQQ was identified as the principal immunogenic sequence, with residues Q1, P4, Q5, Q6, and Q7 being the main contributors to IgE binding [75]. Thus, allergen structure and epitopes significantly influence allergenicity, as discussed in the following sections. The allergen structures and IgE-binding epitope sequences of these allergens are summarized in Table 4.

### 3.5. Tree Nut Allergens

The category of tree nuts includes almond (*Prunus dulcis*), hazelnut (*Corylus avellana*), cashew (*Anacardium occidentale*), and walnut (*Juglans regia*). Other tree nuts include pistachio (*Pistacia vera*), Brazil nut (*Bertholletia excelsa*), macadamia (*Macadamia* spp.), pecan (*Carya illinoinensis*), pine nut (*Pinus* spp.), betel nut (*Areca catechu*), and chestnut (*Castanea* spp.) [76]. In the United States, pecan and cashew allergies are the joint fourth most common tree nut allergies, each affecting 0.5% of American adults. They rank just below almond, walnut, and hazelnut allergies, which affect approximately 0.7%, 0.6%, and 0.6% of adults, respectively [77]. Tree nut allergy is therefore largely attributable to the major tree nuts: almond, walnut, hazelnut, pecan, and cashew.

As shown in Table 5, the WHO/IUIS Allergen Nomenclature Subcommittee has recently registered several official tree nut allergens. The main allergens reported in major tree nuts include Pru du 6 in almonds; Cor a 1, Cor a 8, and Cor a 9 in hazelnuts; Jug r 1-Jug r 4 in walnuts; Ana o 1-Ana o 3 in cashews; and Car i 1 and Car i 4 in pecans [78]. Pru du 6, an 11S globulin also known as amandin or almond major protein, is a hexameric protein that constitutes 65% of the total almond protein [79]. It also exhibits high stability to thermal processing, and 83% of almond-allergic patients show a specific IgE response [80]. Pru du 6 is regarded as a marker of almond allergy. The epitopes are listed in Table 5, of which residues AA113-135, AA120-135, AA209-223, and AA505-519 are the major epitopes [81,82]. Cor a 1 is a 17 kDa pathogenesis-related protein belonging to the Bet v 1 family; reactivity to Cor a 1 was observed in 62.7% of 217 hazelnut-sensitized children [83]. Cor a 8, a 9 kDa type I lipid transfer protein, elicited sensitization in 31.3% of 217 hazelnut-allergic children [83] and exhibited high stability to heat processing and enzymatic treatment [84]. Cor a 9, a 40 kDa 11S seed storage globulin, can cause allergic reactions in children in the United States and Europe [85]. Six epitopes of Cor a 9 are listed in Table 5, of which residues AA201-215, AA241-255, and AA281-295 are major epitopes [82]. Among the main walnut allergens, Jug r 1, Jug r 2, Jug r 3, and Jug r 4 are recognized by the serum IgE of 75%, 60%, 80%, and 65% of walnut-allergic patients, respectively [84,86]. Jug r 1 also exhibits high stability during thermal processing and enzymatic treatment [87]. Ten epitopes of Jug r 1 are listed in Table 5, of which residues AA65-73 and AA95-117 are the major epitopes [88]. Five epitopes of Jug r 4 were also listed, of which residues AA233-247, AA253-271, and AA281-295 were the key epitopes [82]. Among the main cashew allergens, Ana o 1, Ana o 2, and Ana o 3 are recognized by 50%, 62%, and 81% of cashew-allergic patients, respectively [89,90,91]. Ana o 2 and Ana o 3 exhibit high stability during thermal processing and are regarded as markers of cashew allergy [92]. As shown in Table 5, eleven, twenty-nine, and thirty-seven epitopes were identified in Ana o 1, Ana o 2, and Ana o 3, respectively. These include major epitopes at residues AA1-15, AA57-71, and AA521-535 in Ana o 1 [89]; residues AA15-29, AA105-119, AA185-199, AA233-247, AA241-255, AA257-271, and AA425-439 in Ana o 2 [93]; and residues AA33-44, AA54-65, AA57-68, AA72-83, and AA102-113 in Ana o 3 [91]. Among the main pecan allergens, Car i 1 and Car i 4 are recognized by 79% and 59% of pecan-allergic patients, respectively [82,88]. Twenty-five epitopes of Car i 1 are listed in Table 5, of which residues AA43-54, AA46-57, AA67-78, AA106-117, and AA109-120 are major epitopes [88]. Nineteen epitopes were identified in Car i 4, of which residues AA88-99, AA118-129, AA121-132, AA124-135, AA127-138, AA202-213, AA205-216, AA208-219, and AA238-249 were the major epitopes [82].

## 4. Relationships Between Allergen Characteristics, Clinical Relevance, and Food Processing

This review systematically compares the molecular features of five major allergen families from plant-derived foods, revealing how their structural properties determine their thermal stability, digestibility, sensitization potential, and clinical severity. These families, 2S albumins, 7S/11S globulins, PR-10 proteins, profilins, and non-specific lipid transfer proteins (nsLTPs), exhibit structural differences that directly shape their allergenic behavior (Table 1, Table 2, Table 3, Table 4 and Table 5).

2S albumins are low-molecular-weight (7–17 kDa) seed storage proteins, structurally defined by 4–5 intramolecular disulfide bonds [22,24], which confer a highly compact, rigid three-dimensional fold. This architecture provides exceptional heat stability and proteolytic resistance. Ara h 2 and Ara h 6 from peanut are archetypal examples: they retain intact conformations after heating and pepsin treatment, thereby passing through the gastrointestinal tract in their native form to engage the intestinal immune system and provoke robust Th2-skewed responses. Likewise, Ses i 1 from sesame (100% recognition), Jug r 1 from walnut (75%), and Ana o 3 from cashew (81%) are also 2S albumins [69,78] with high sensitization frequencies. These observations suggest that disulfide bond density may serve as a key structural parameter for predicting the sensitization potency of 2S albumins.

7S (vicilin) and 11S (legumin) globulins are the most abundant seed storage proteins, assembling into trimeric and hexameric oligomers, respectively. 7S globulins are more heavily glycosylated, whereas 11S globulins consist of acidic and basic subunits linked by disulfide bonds. Compared to 2S albumins, globulins display moderate thermal stability; heat treatment dissociates their oligomers, yet linear epitopes within the subunits often remain intact. Together with Ara h 2, peanut Ara h 1 (7S) and Ara h 3 (11S) account for approximately 95% of serum IgE reactivity among peanut-allergic patients [15]. Soybean Gly m 5 (7S) and Gly m 6 (11S) are major targets in pediatric soy allergy [36]. Notably, the basic subunits of 11S globulins (e.g., walnut Jug r 4 and hazelnut Cor a 9) retain partially intact conformational epitopes after heat-induced aggregation owing to their hydrophobic cores and can still induce strong IgE cross-linking.

PR-10 proteins (e.g., soybean Gly m 4 and hazelnut Cor a 1) are Bet v 1 homologs composed of three α-helices and eight β-strands that enclose an internal cavity. Because they lack disulfide bonds, their structural stability relies predominantly on hydrophobic interactions and hydrogen bonds, rendering them susceptible to heat, acid, and proteolytic degradation [7]. However, this fragility does not equate to a low clinical risk. Gly m 4 can elicit severe systemic reactions, particularly in patients not co-sensitized to Gly m 5 or Gly m 6 [35], underscoring that degradation sensitivity and disease severity are not simply inversely correlated. Rather, the allergenicity of PR-10 proteins depends more on their cross-reactivity with Bet v 1 (pollen–food syndrome) than on their intrinsic stability.

Profilins (e.g., peanut Ara h 5 and soybean Gly m 3) are 12–15 kDa actin-binding proteins consisting of seven antiparallel β-strands surrounding a central α-helix. They lack disulfide bonds, are highly thermolabile, and are readily destroyed by heating or digestion. Clinical recognition rates are generally low; for example, peanut Ara h 5 was recognized by 9.1% of peanut-allergic patients [14], and symptoms are mostly confined to oral allergy syndrome. Profilins are highly sequence-conserved, making them pan-allergens with extensive cross-reactivity across different plant species. However, their clinical relevance is limited, and they are rarely used as primary sensitizers.

Non-specific lipid transfer proteins (e.g., wheat Tri a 14, peanut Ara h 9, and walnut Jug r 3) are small (~9 kDa) proteins with four α-helices cross-linked by four disulfide bonds, forming a central hydrophobic cavity for lipid binding. This highly stable architecture confers pronounced heat tolerance and resistance to pepsin digestion [70,94]. nsLTPs are abundant in the plant epidermis and are commonly found in fruits and nuts. Wheat Tri a 14 is recognized by 60% of bakers with asthma [95]; because it is present in flour dust, respiratory exposure can cause sensitization even without prior digestion.

From a processing perspective, structural stability determines the differential efficacy of various food processing methods. Thermal treatments (e.g., boiling and roasting) effectively destroy the epitopes of PR-10 proteins and profilins but have a limited impact on disulfide-rich 2S albumins, nsLTPs, and 11S globulins. High-pressure processing can induce the dissociation of globulin oligomers, potentially exposing neoepitopes. Fermentation and enzymatic hydrolysis cleave peptide bonds to disrupt linear epitopes; however, the compact architecture of 2S albumins restricts enzyme accessibility. The Maillard reaction can modify epitope lysine residues, reducing IgE binding in some cases, but may also generate advanced glycation end products or neoantigens [96]. Therefore, tailored hypoallergenization strategies must be developed for different allergen families rather than relying on a uniform approach.

In summary, structural features (e.g., disulfide bond count, oligomeric state, hydrophobic core, and surface charge distribution) constitute the molecular basis for the thermal stability, digestibility, and sensitization potential of allergens. Future research should focus on elucidating the dynamic structural changes in allergens under processing conditions and the mechanisms of conformational epitope disruption, thereby providing theoretical guidance for the development of precision hypoallergenic technologies.

## 5. Computational Analysis of Epitope Conservation and Cross-Species Structural Comparison

Systematic epitope conservation analyses have revealed evolutionary patterns of IgE-binding sites across major plant allergen families. Within the cupin superfamily (7S/11S globulins), cross-species alignment of linear epitopes from peanut (Ara h 3), soybean (Gly m 6), hazelnut (Cor a 9), walnut (Jug r 4), and cashew Ana o 2 identified multiple conserved antigenic regions distributed on both the subunit surface and oligomeric interfaces, explaining why heat-induced dissociation can unmask hidden epitopes and alter allergenicity [97]. In the prolamin superfamily, 2S albumins (e.g., Ara h 2, Ses i 1, and Jug r 1) maintain similar conformational epitopes due to their highly conserved disulfide frameworks, while epitope mapping of non-specific lipid transfer proteins (nsLTPs) has uncovered conserved B-cell epitopic motifs shared among chickpea, mung bean, peanut, and lentil, suggesting evolutionary conservation within this family [98]. For PR-10 proteins and profilins, although thermolabile, epitope analyses indicate that up to 60% of IgE binding is mediated by species-specific epitopes, whereas cross-reactive epitopes determine the breadth of clinical symptoms [99]. Recently, the A-RISC (Allergen Reactivity Index for Structural Conservation) has enabled quantitative risk assessment of cross-reactivity, revealing high homology (A-RISC > 0.75) for PR-10 proteins, moderate similarity (0.45–0.57) for vicilins and glycinins, and lower scores for nsLTPs and 2S albumins, yet with conserved motifs in immunologically relevant regions [100]. These findings underscore the central role of epitope conservation in predicting cross-reactivity and guiding allergen-mitigation strategies.

Cross-species structural comparisons using three-dimensional homology modeling and surface accessibility calculations have uncovered cross-reactivity determinants that are not apparent from sequence similarity alone. Jenkins et al. [101] classified 129 plant allergens into Pfam families and found that more than 65% belong to only four families, indicating that conserved three-dimensional folds, rather than primary sequence, are key drivers of allergenicity. Homology modeling of Bet v 1-related proteins demonstrated that despite only ~56% sequence identity, their surface structural similarity can reach 75%, sufficient to support cross-recognition by anti-Bet v 1 antibodies [101]. For nsLTPs, structures within the Rosaceae family are highly similar, whereas significant amino acid replacements occur in corresponding regions of nsLTPs from distantly related families, explaining the extensive cross-reactivity among Rosaceae fruits and the more limited cross-reactivity across distant taxa [98]. Comparative crystallography of profilins from diverse plants revealed virtually identical backbone conformations but distinct surface residue exposures that define species-specific epitopes, thereby influencing individual clinical phenotypes [99]. Structural comparisons have also shown that globulin epitopes are not confined to the surface; some are buried in the native oligomeric state but may become exposed during processing—a finding with critical implications for designing effective processing-based hypoallergenization strategies. Collectively, these data emphasize that structure-based rather than sequence-based assessments are essential when evaluating the allergenic potential of novel or engineered food proteins.

In summary, computational analysis of epitope conservation and cross-species structural comparison provides a valuable complement to experimental epitope mapping. By revealing evolutionarily conserved allergenic motifs and predicting cross-reactivity risks, these in silico approaches can guide both clinical diagnosis and the rational design of hypoallergenic food products. Future research should integrate high-resolution structural data with patient-specific IgE epitope profiles to enable personalized risk assessment and targeted allergen mitigation strategies.

## 6. Limitations and Future Directions

Although this review provides a comprehensive overview of the key characteristics of plant allergens, it has several limitations. First, current clinical sensitization data are derived mainly from Europe and North America, and data on population differences, age-related differences (children versus adults), and regional characteristics of plant food allergies in Asia, Africa, and other regions are lacking. Second, for most of the minor allergens listed in the tables, high-resolution crystal structures and information on dynamic structural changes under processing conditions are lacking. Moreover, the conformational epitopes of many allergens have not been identified because most studies have focused only on linear epitopes. Third, standardized reference materials are absent for many plant food allergens, complicating cross-study comparisons and method harmonization. In addition, the clinical validation of many identified epitopes across diverse patient populations remains limited.

To address these gaps, future research should pursue the following directions. First, large-scale cohort studies should be conducted in different regions to clarify population-specific sensitization characteristics and to update epidemiological data. Second, the molecular mechanisms by which processing (e.g., heating, high-pressure treatment, and fermentation) alters allergen structure and disrupts epitopes should be analyzed in depth. Third, the cross-reactivity mechanisms of multiple co-occurring allergens and the clinical risks associated with mixed plant-based food allergies should be evaluated. Fourth, unified detection standards for multiple plant allergens should be established, and standardized hypoallergenic plant food formulations should be developed. Fifth, multi-allergen epitope detection chips should be developed, component-resolved diagnosis should be optimized, and epitope-based, specific immunotherapy vaccines should be designed.

## 7. Conclusions

In summary, incorporating plant-based foods into the daily diet provides substantial nutritional benefits. However, because major plant allergenic foods such as soybeans, peanuts, wheat, sesame, and tree nuts contain various allergenic proteins and epitopes, they frequently trigger allergic reactions, which severely limits their use and may endanger human health. To date, only a limited number of major allergens and their epitopes from certain plant sources, such as tree nuts, have been identified. Therefore, comprehensive investigations and characterizations of allergens in all plant allergenic foods are crucial for future research.

## Figures and Tables

**Table 2:** The characteristics of soybean allergens [7,27,28,52].

Allergen	Biochemical Name	MW/kDa (SDS-PAGE)	Structure	Recognition by Sesame-Allergic Patients	Isomer	Epitopes
Gly m 1	Hydrophobic protein from soybean	7	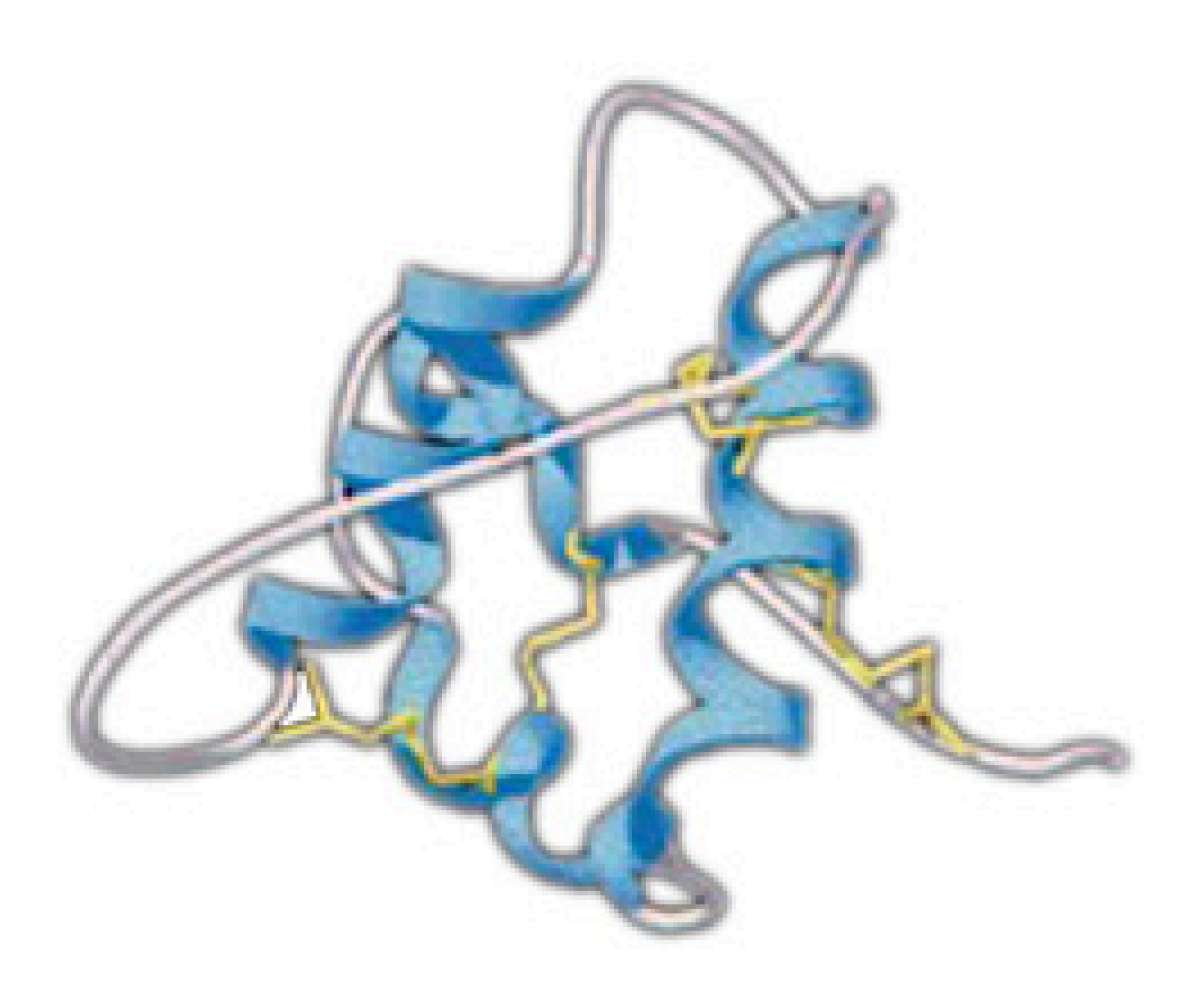	90%	Gly m 1.0101 Gly m 1.0102	
Gly m 2	Defensin	8		100%		
Gly m 3	Profilin	14	** 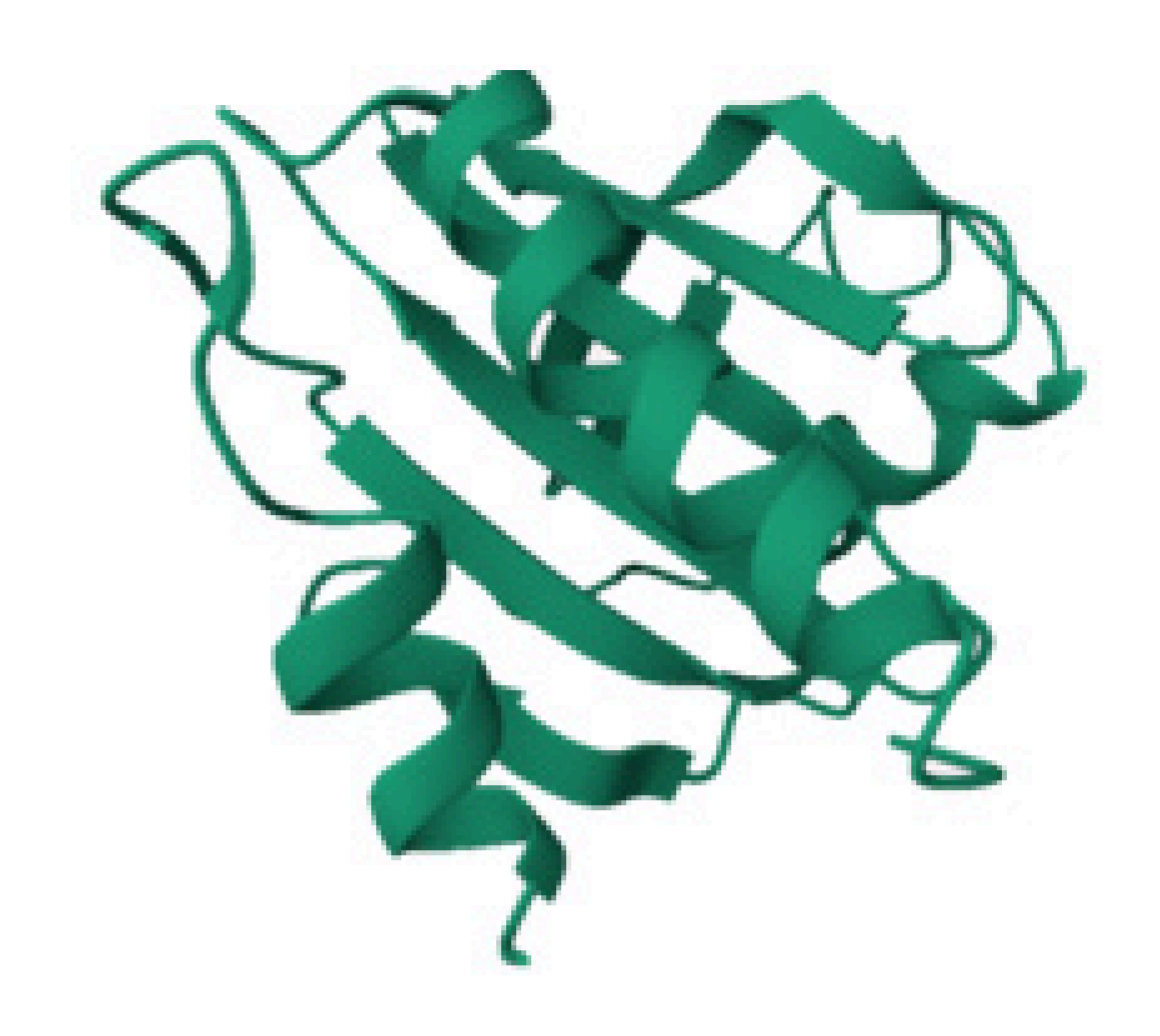 **	67%	Gly m 3.0101 Gly m 3.0102	
Gly m 4	Pathogenesis-related protein, PR-10, Bet v 1 family member	17	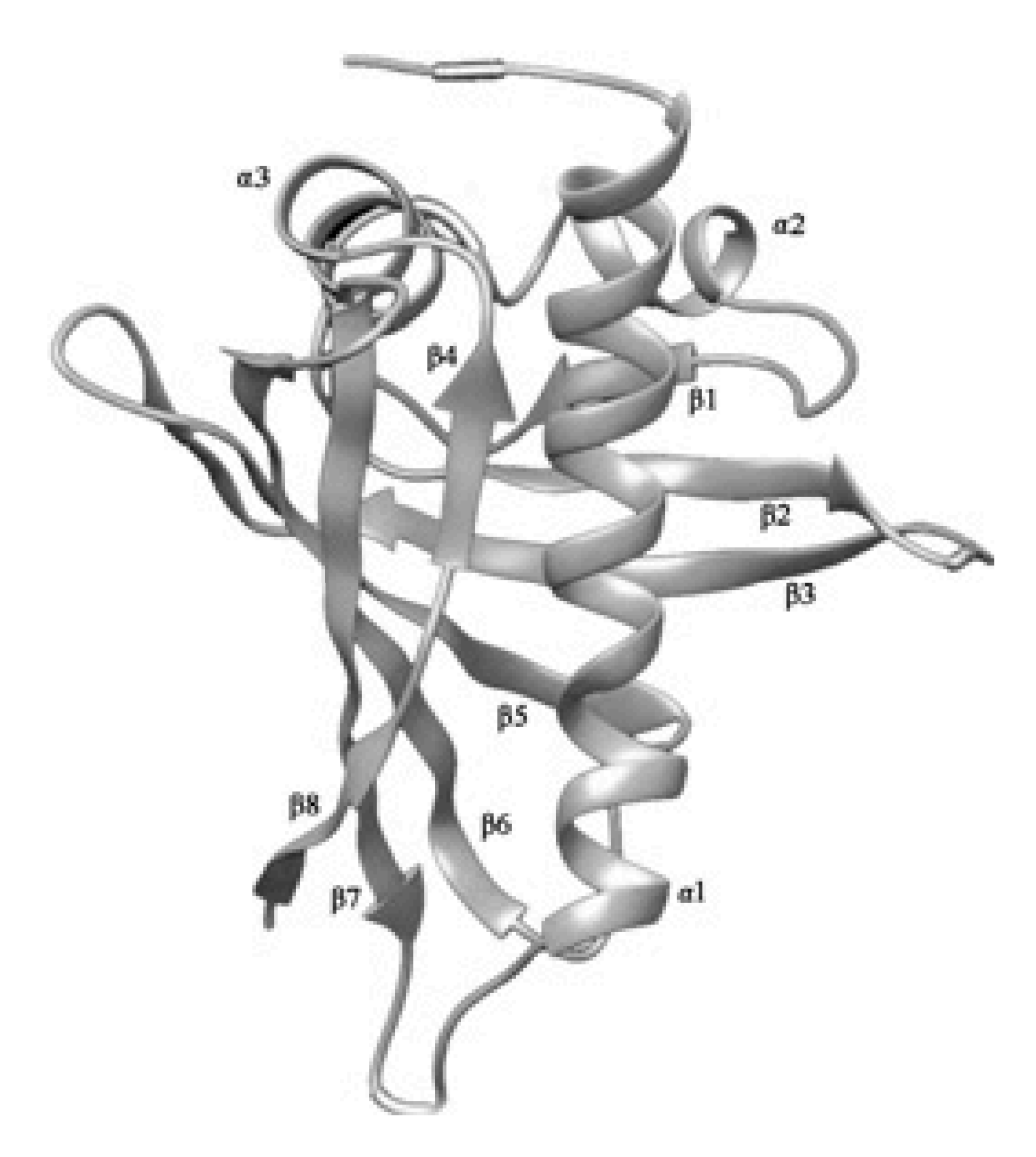			AA3-7, AA24-28, AA44-54, AA41-47, AA74-77 and AA78-83
Gly m 5	Beta-conglycinin (vicilin, 7S globulin)		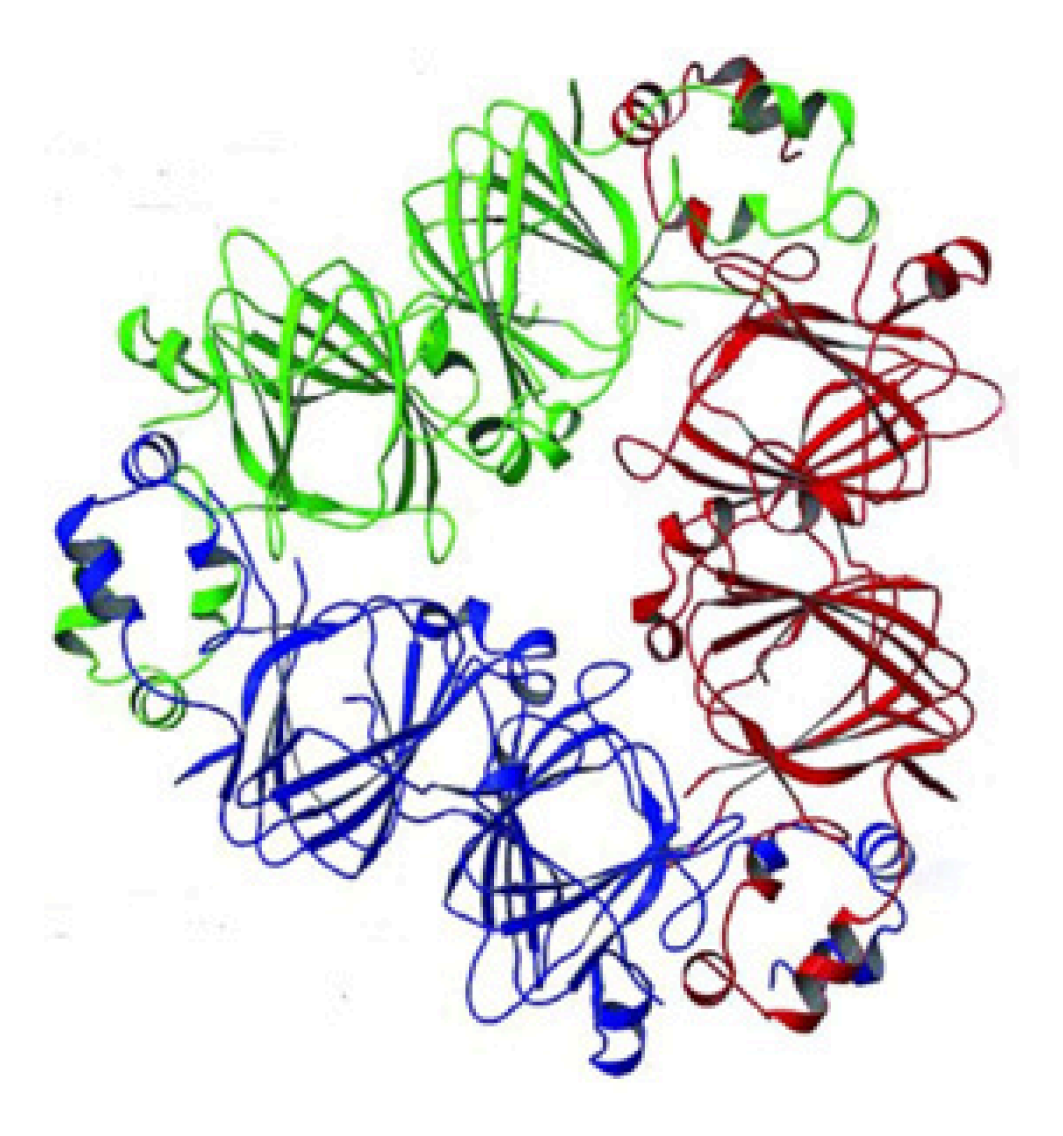		Gly m 5.0101 Gly m 5.0201 Gly m 5.0301 Gly m 5.0302	AA29-40, AA85-95, AA209-221, AA524-534, AA380-396, AA380-408, AA404-448, AA5-33, AA90-115, AA55-80, AA110-135, AA130-158, AA200-229, AA230-260, AA320-345, AA338-368, AA350-375 and AA510-540
Gly m 6	Glycinin (legumin, 11S globulin)		** 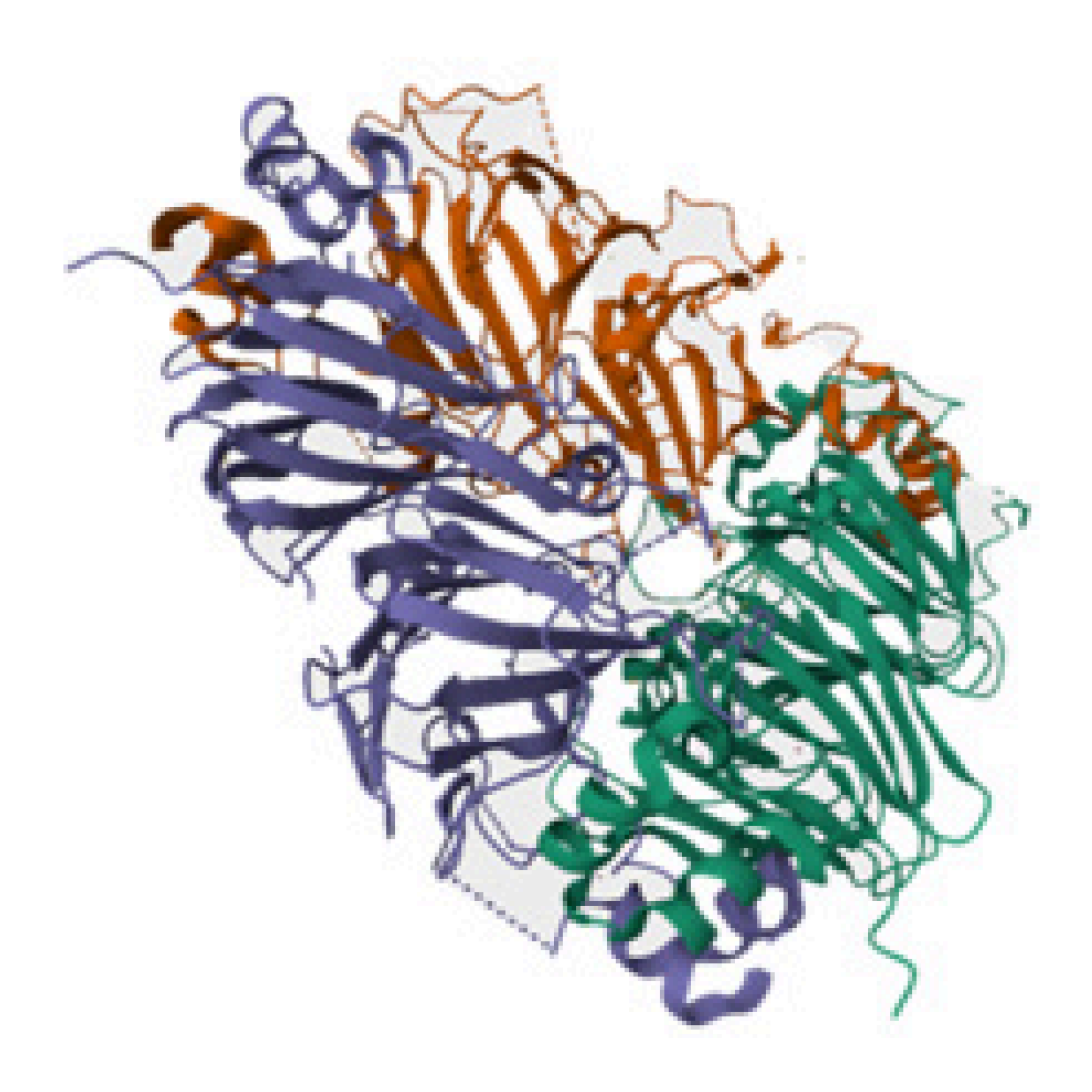 **		Gly m 6.0101 Gly m 6.0201 Gly m 6.0301 Gly m 6.0401 Gly m 6.0501	AA217-235, AA219-233, AA253-265, AA3-17, AA83-97, AA90-104, AA177-191, AA189-203, AA255-269, AA337-351, AA345-359, AA358-372, AA367-381 and AA451-465
Gly m 7	Seed biotinylated protein; late embryogenesis abundant protein	76.2		78%		
Gly m 8	2S albumin	28	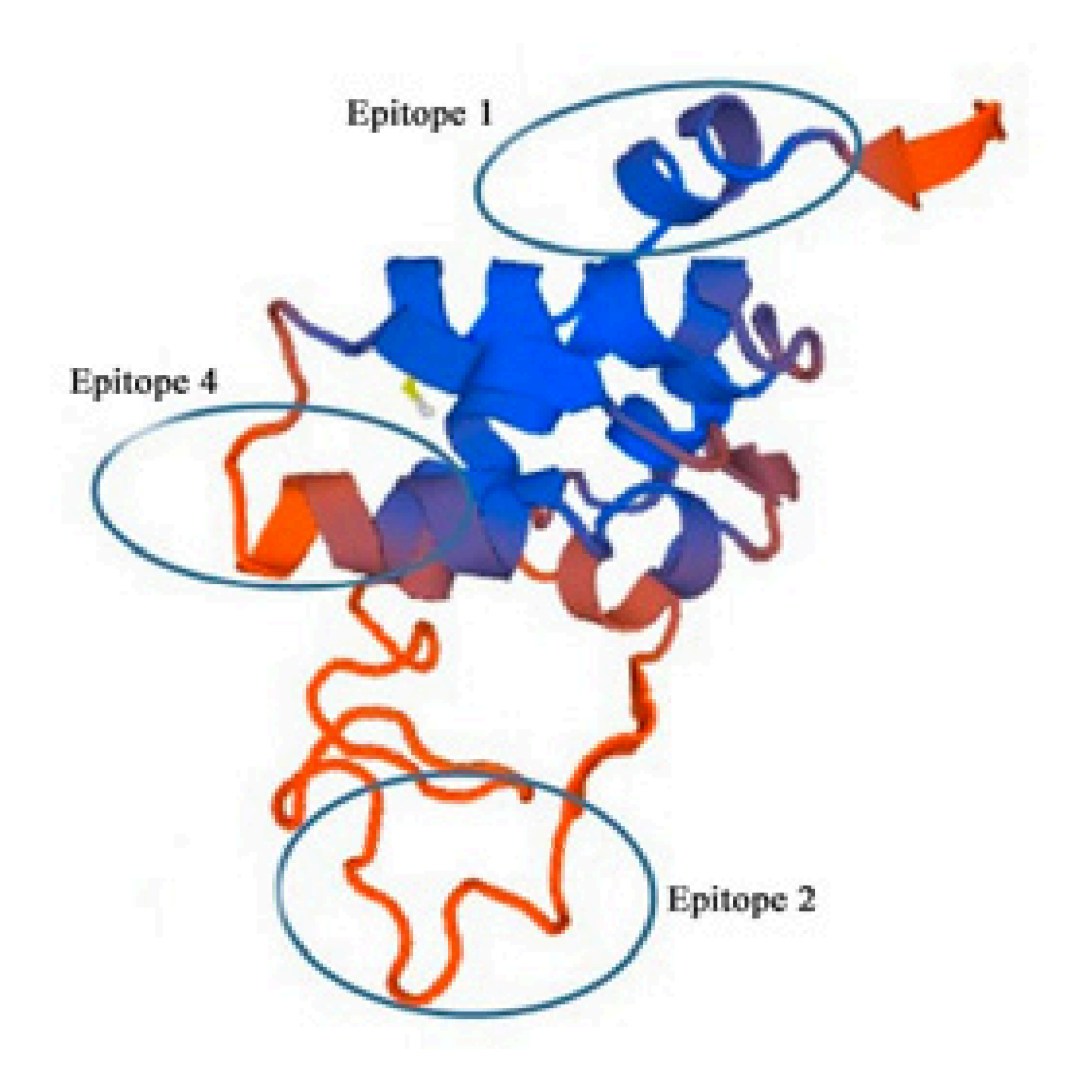			

**Table 3:** The characteristics of sesame allergens [27,28,69].

Allergen	Biochemical Name	MW/kDa (SDS-PAGE)	Structure	Recognition by Sesame-Allergic Patients	Isomer	Epitopes
Ses i 1	2S albumin	9	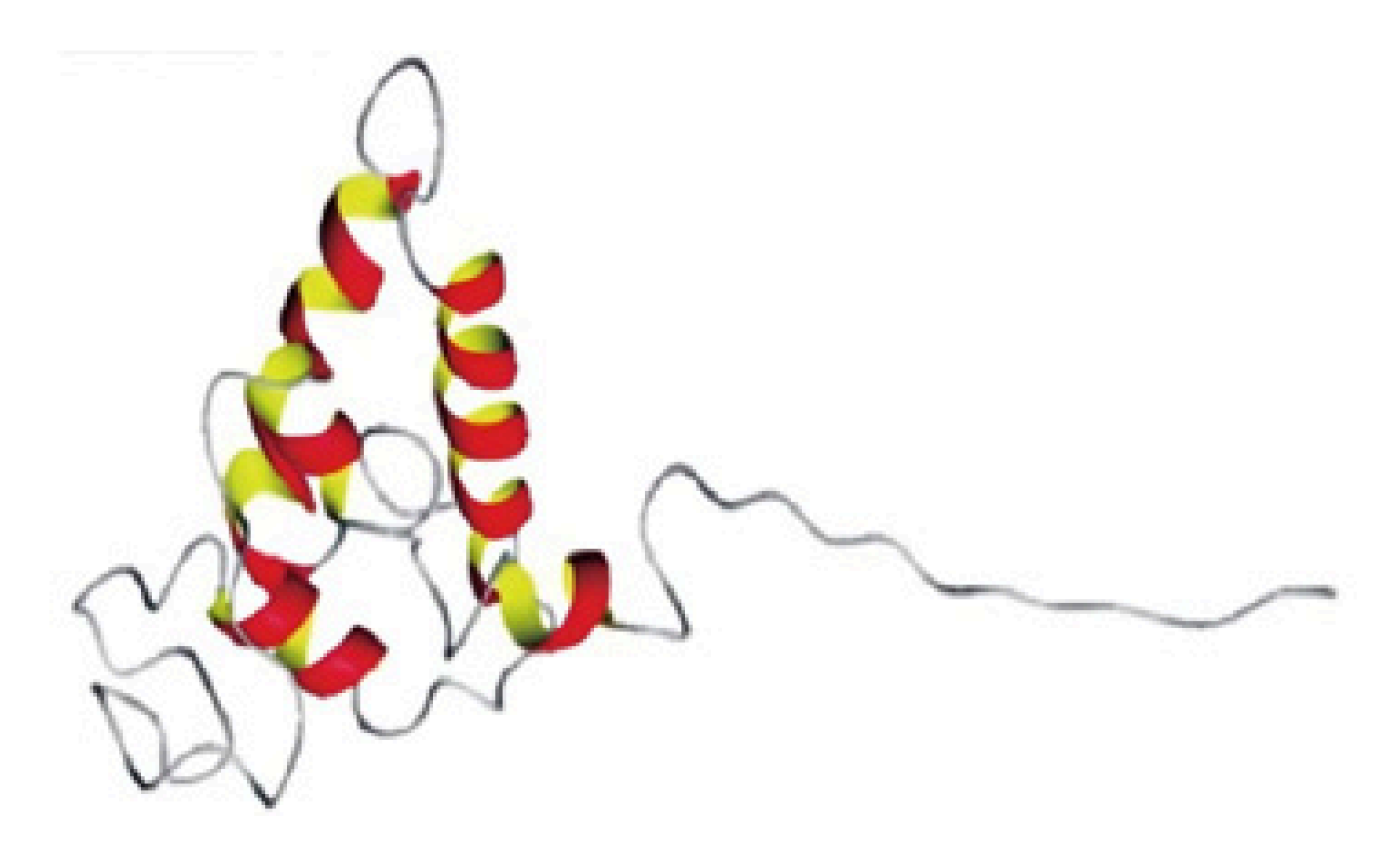	100%	Ses i 1.0101	
Ses i 2	2S albumin	7	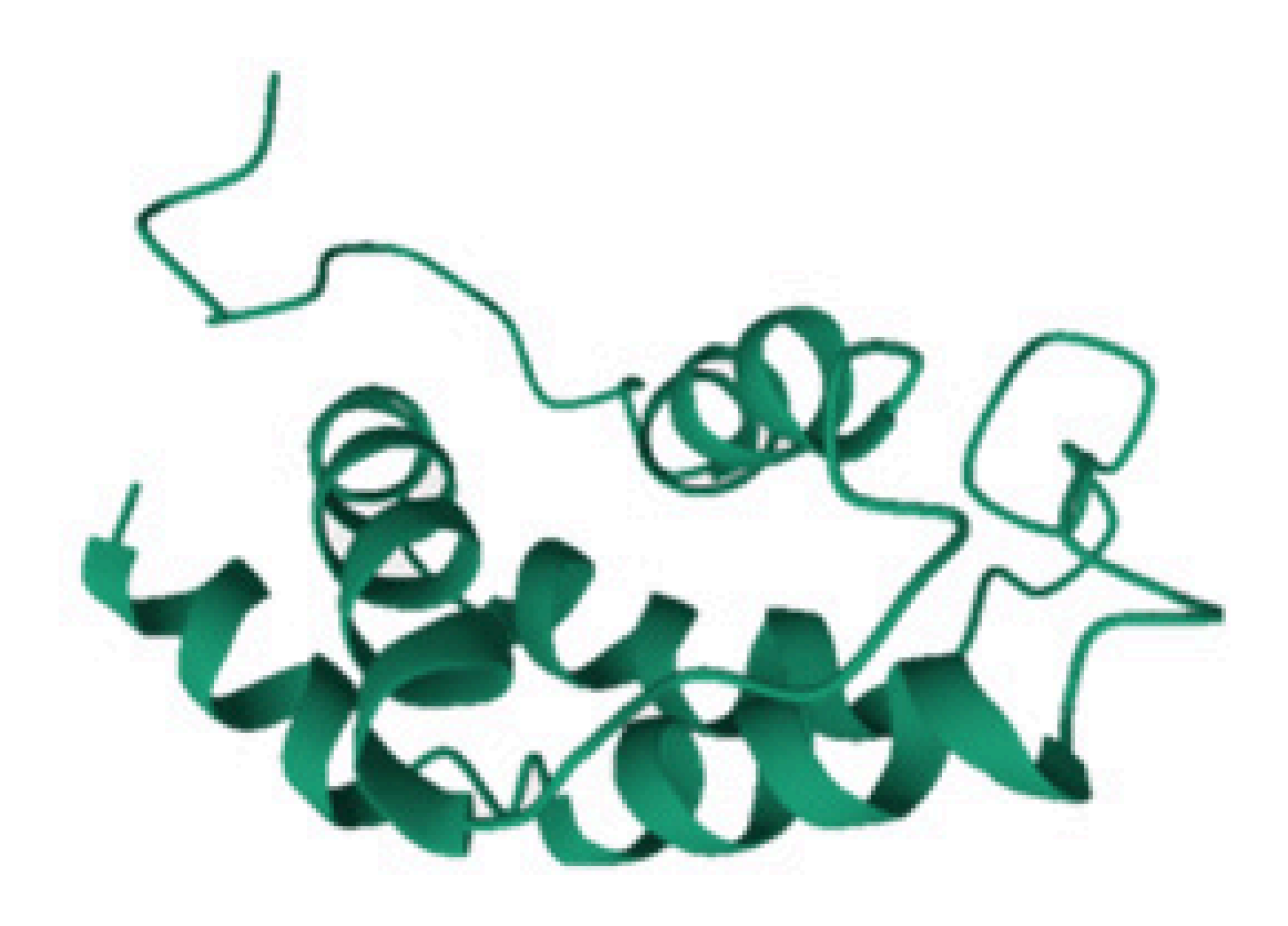	66.7%	Ses i 2.0101	AA46-55, AA48-57, AA54-63, AA74-83, AA76-86, AA78-87, AA80-89, AA82-91 and AA84-94
Ses i 3	7S vicilin-like globulin	45	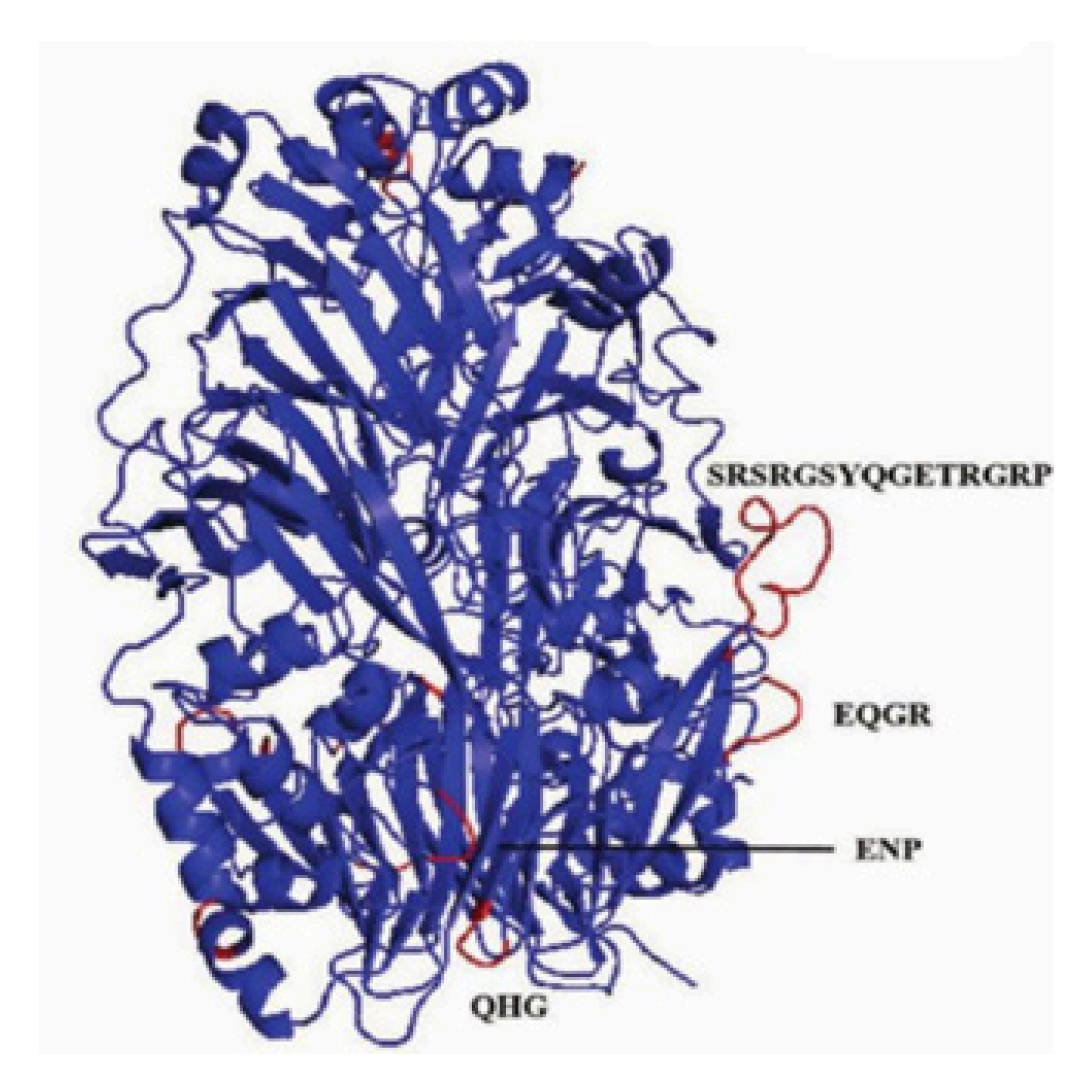	60%	Ses i 3.0101	AA23-28, AA60-68, AA81-85, AA99-101, AA115-134, AA142-145, AA162-175, AA178-180, AA188-191, AA210-212, AA270-273, AA328-330, AA378-380, AA390-392, AA401-404, AA16-418, AA469-483, AA525-529, AA563-567 and AA574-582
Ses i 4	Oleosin	17		100%	Ses i 4.0101	
Ses i 5	Oleosin	15		100%	Ses i 5.0101	
Ses i 6	11S globulin	52		52%	Ses i 6.0101	
Ses i 7	11S globulin	57		40%	Ses i 7.0101	

**Table 4:** The characteristics of wheat allergens [27,28,73].

Allergen	Biochemical Name	MW (SDS-PAGE)	Structure	Recognition by Sesame-Allergic Patients	Isomer	Epitopes
Tri a 12	Profilin	14 kDa	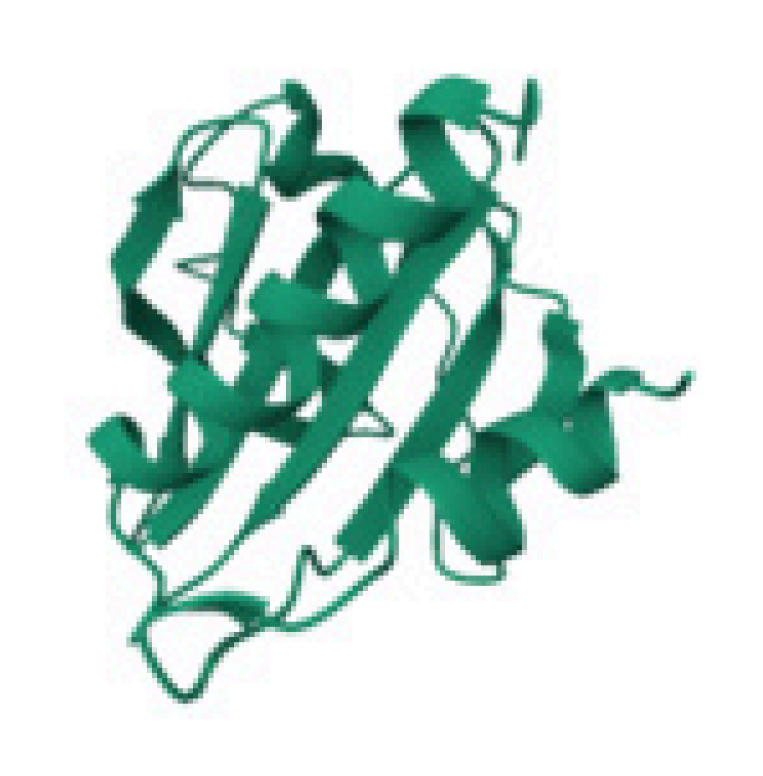		Tri a 12.0101 Tri a 12.0102 Tri a 12.0103 Tri a 12.0104	
Tri a 14	Non-specific lipid transfer protein 1 (nsLTP-1)	9 kDa	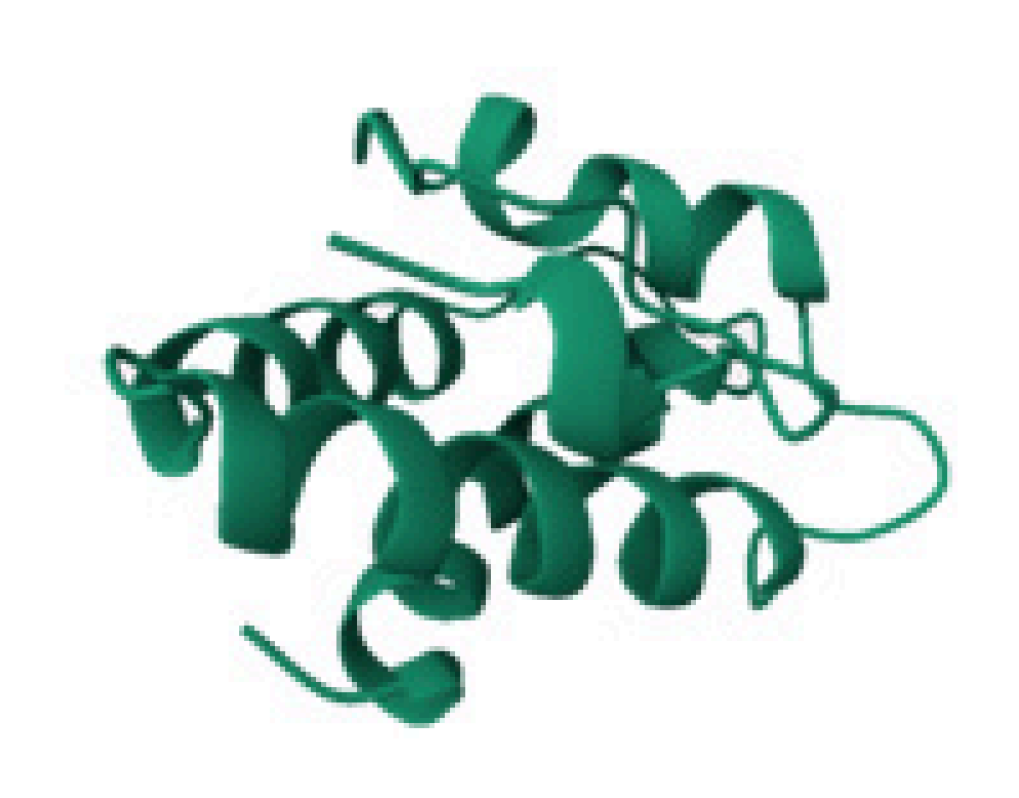	60%	Tri a 14.0101 Tri a 14.0201	AA11-20, AA24-33, AA31-46, AA37-46, AA53-62, AA71-80 and AA81-90
Tri a 15	Monomeric alpha-amylase inhibitor 0.28	12		10%	Tri a 15.0101	
Tri a 17	beta-amylase	17 kDa	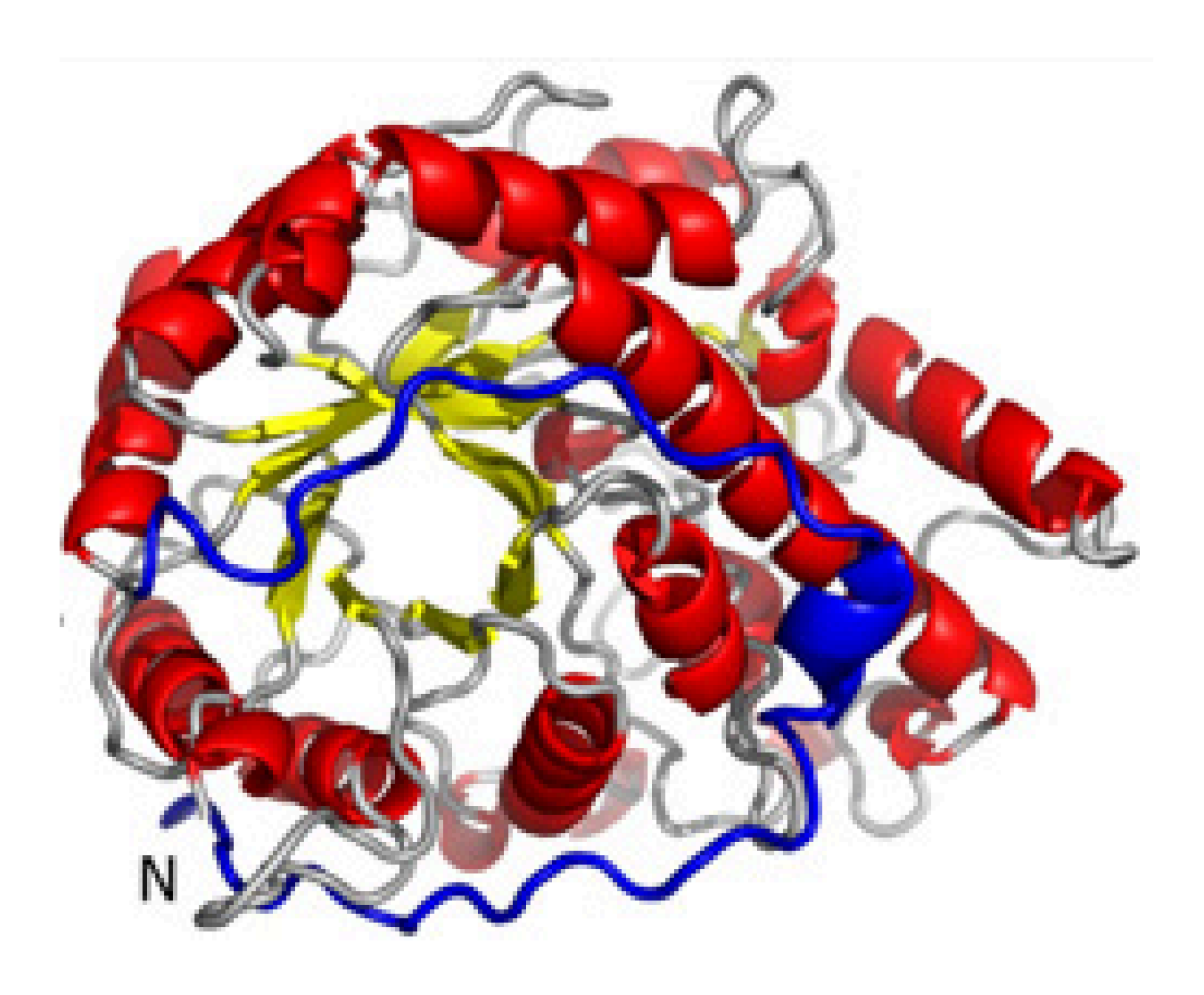	41%	Tri a 17.0101	
Tri a 18	Agglutinin isolectin 1	17	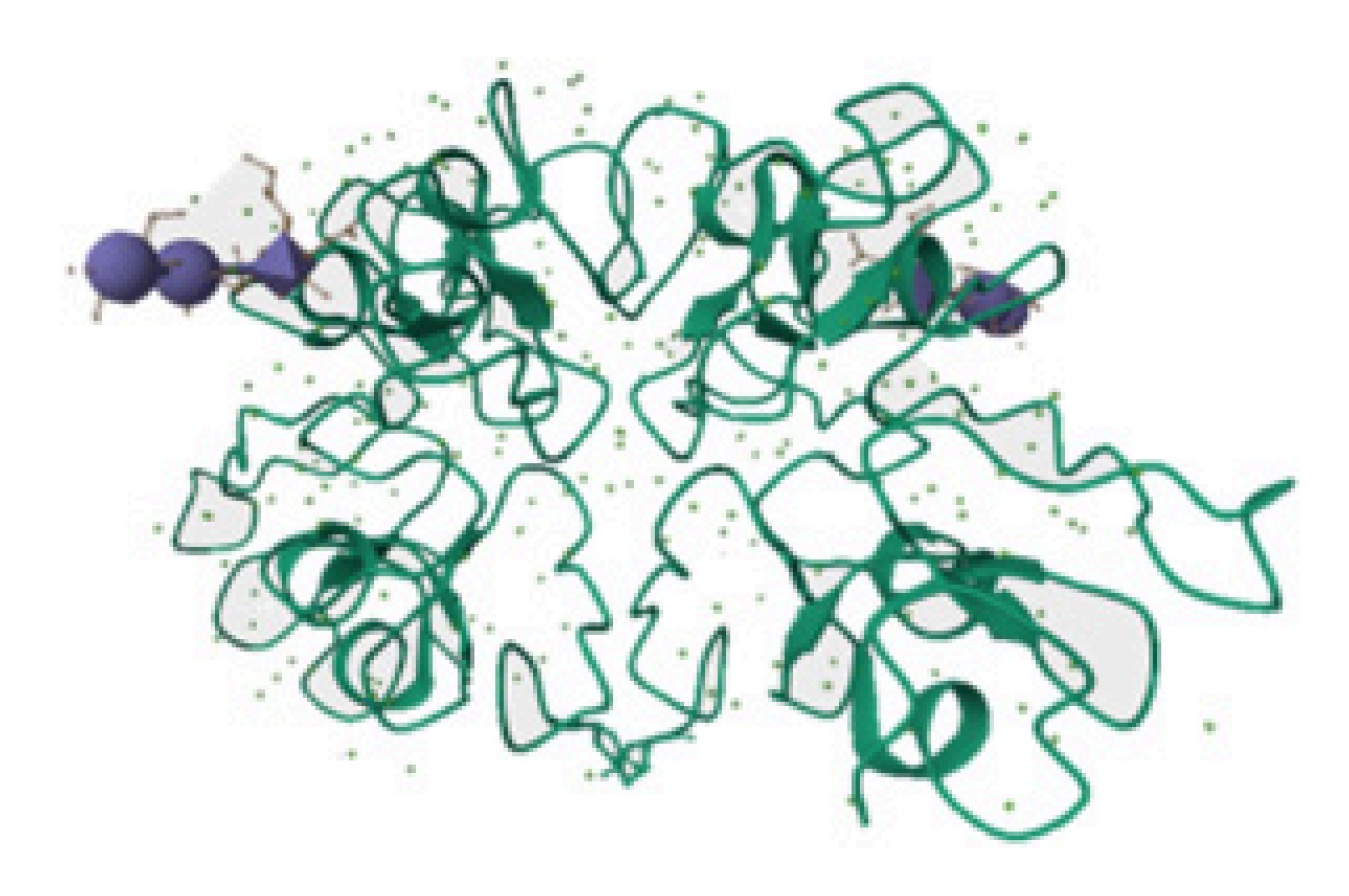		Tri a 18.0101	
Tri a 19	Omega-5 gliadin	65 kDa	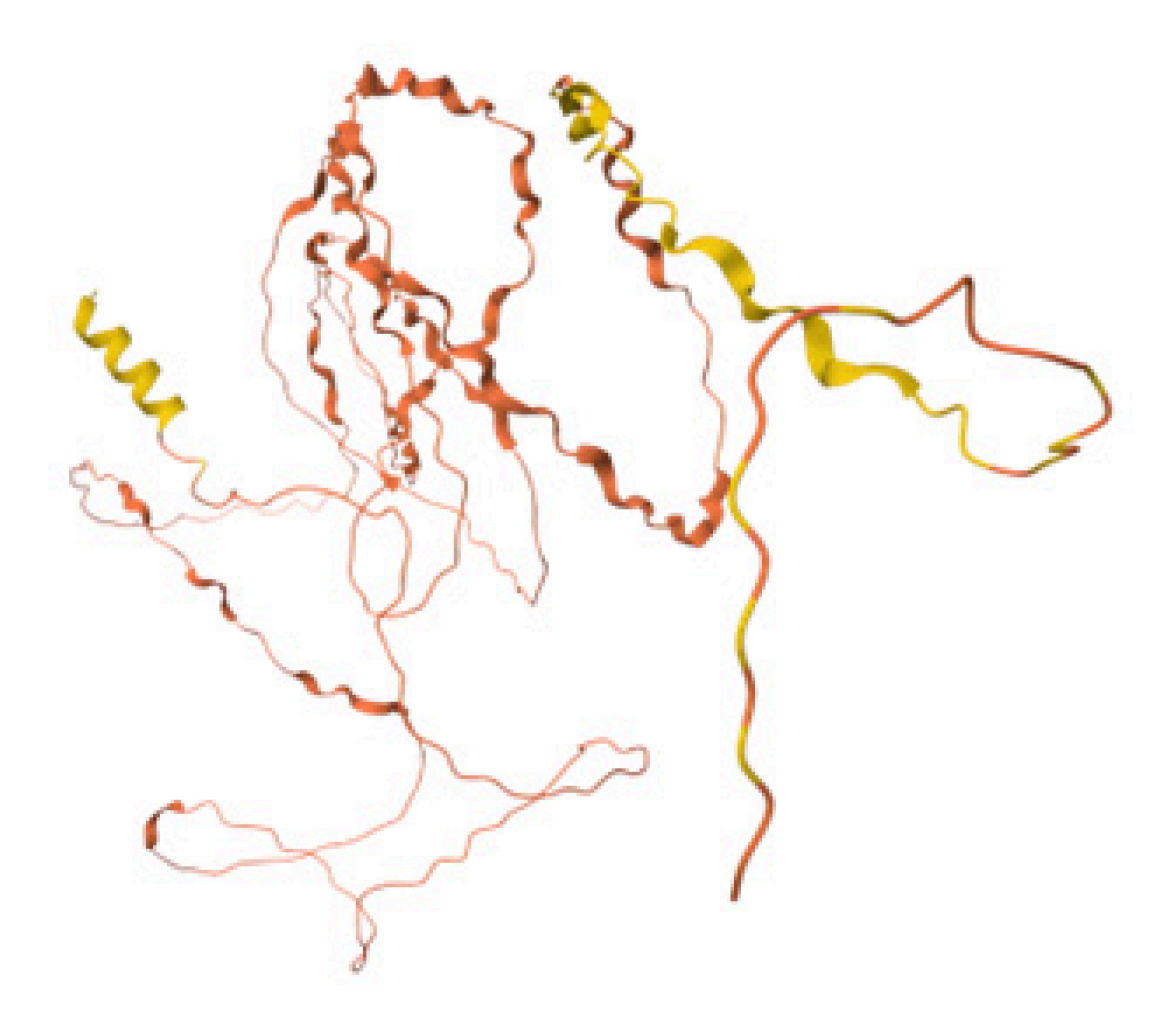	100%	Tri a 19.0101	AA18-27, AA26-35, AA30-39, AA42-51, AA46-55, AA56-64, AA75-84, AA100-109, AA111-120, AA116-125, AA120-129, AA148-157, AA152-161, AA156-165, AA159-168, AA163-172, AA166-175, AA170-179, AA174-183, AA184-193, AA186-195, AA198-207, AA208-217, AA211-220, AA230-239, AA251-260, AA268-277, AA274-283 and AA310-319
Tri a 20	Gamma gliadin	35–38 kDa	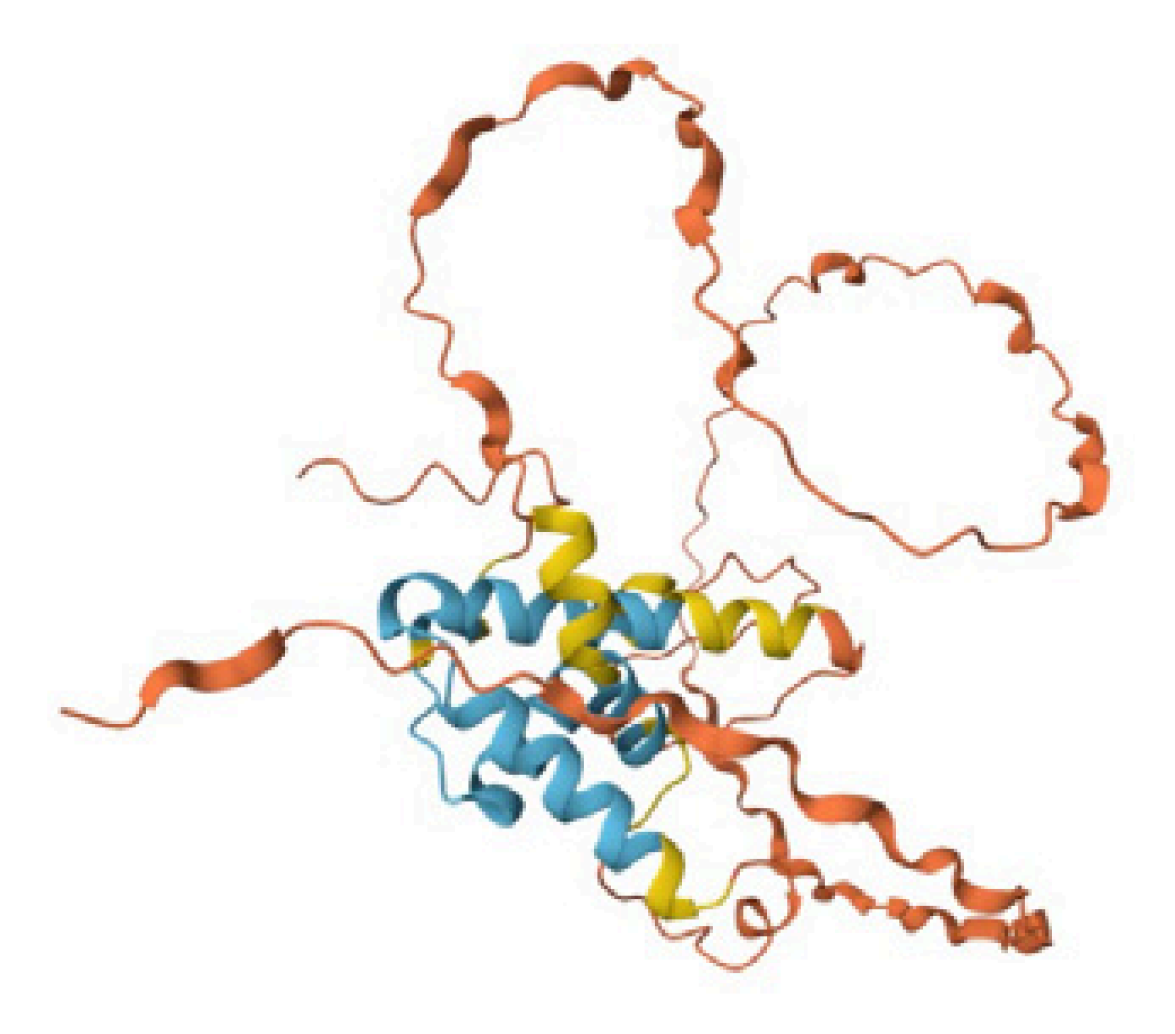		Tri a 20.0101 Tri a 20.0201 Tri a 20.0301	AA13-22, AA15-24, AA93-102, AA133-142, AA141-150, AA143-152 and AA202-211
Tri a 21	Alpha/beta gliadin	28–35	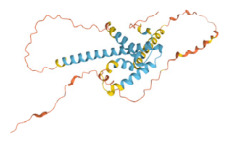		Tri a 21.0101 Tri a 21.0201 Tri a 21.0301 Tri a 21.0401 Tri a 21.0501	AA19-28, AA21-30, AA25-34, AA27-36, AA29-38, AA123-132, AA166-175, AA167-176, AA193-202, AA223-232 and AA279-288
Tri a 25	Thioredoxin	13	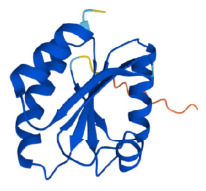		Tri a 25.0101	
Tri a 26	High-molecular-weight glutenin	88 kDa	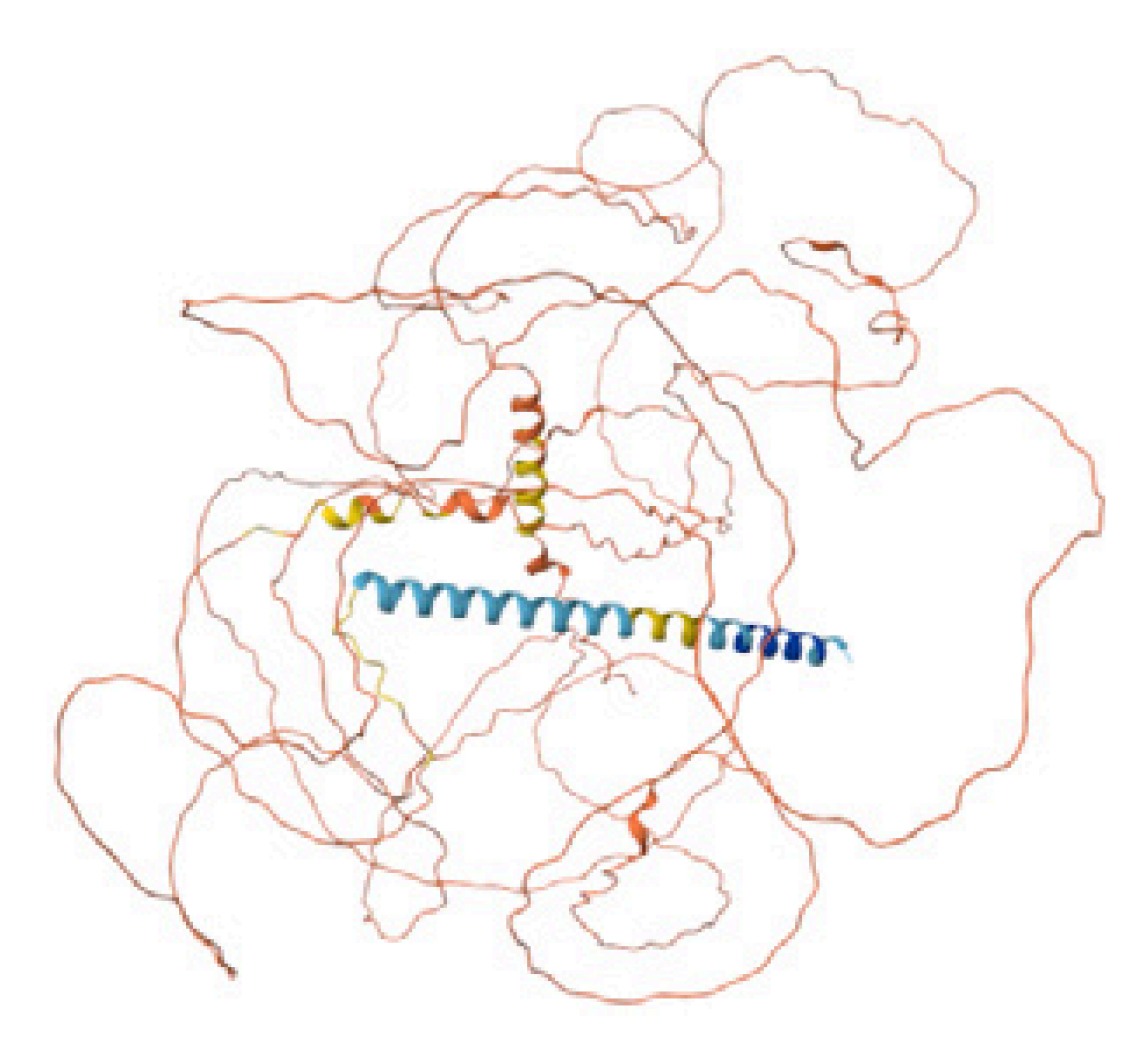		Tri a 26.0101 Tri a 26.0201	AA146-150, AA146-152 and AA280-286
Tri a 27	Thiol reductase homologue	27 kDa		15.8%	Tri a 27.0101	
Tri a 28	Dimeric alpha-amylase inhibitor 0.19	13 kDa		25%	Tri a 28.0101	
Tri a 29	Tetrameric alpha-amylase inhibitor CM1/CM2	13 kDa			Tri a 29.0101 Tri a 29.0201	
Tri a 30	Tetrameric alpha-amylase inhibitor CM3	16 kDa	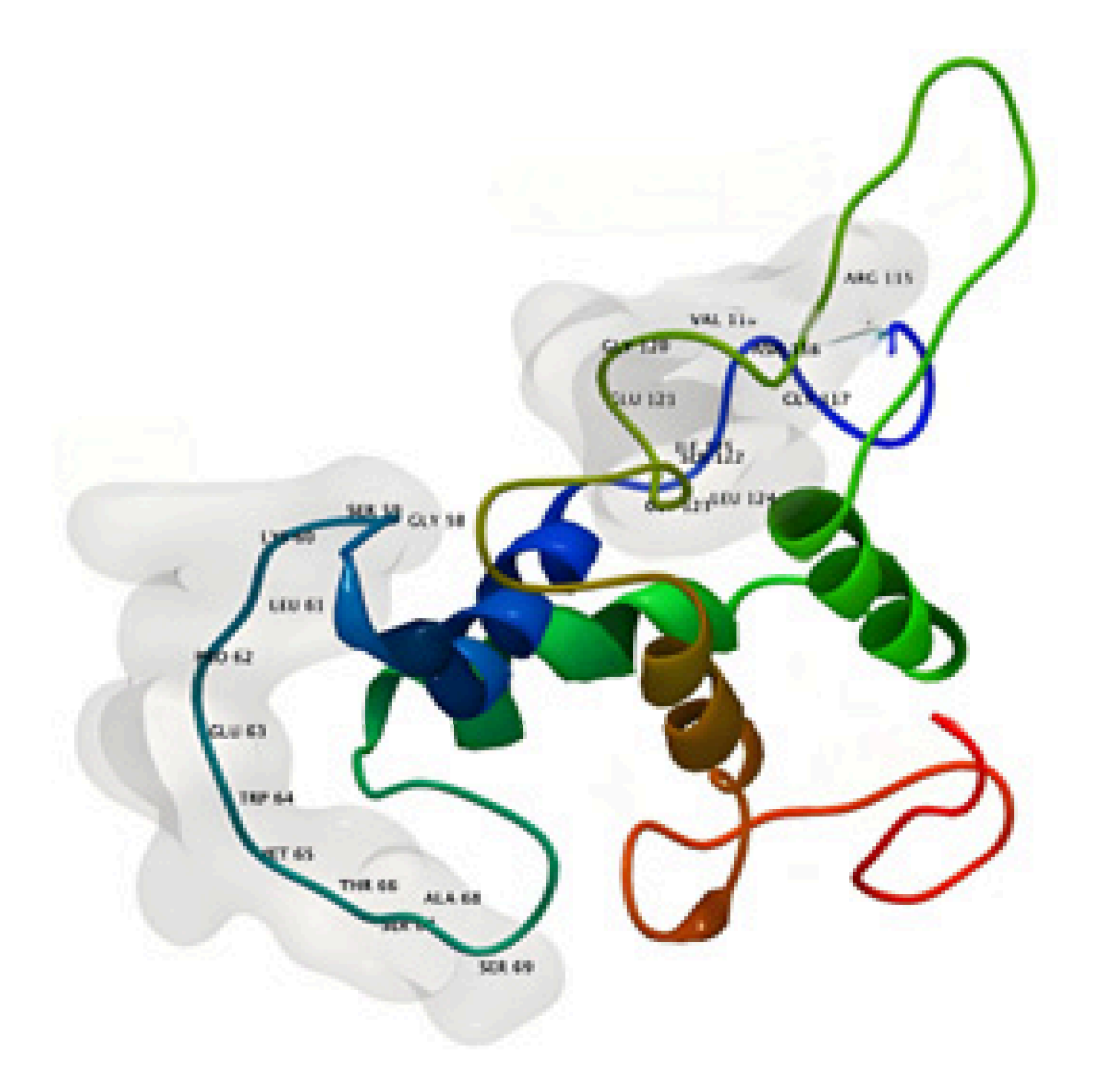		Tri a 30.0101	AA58-69 and AA115-125
Tri a 31	Triosephosphate isomerase	26	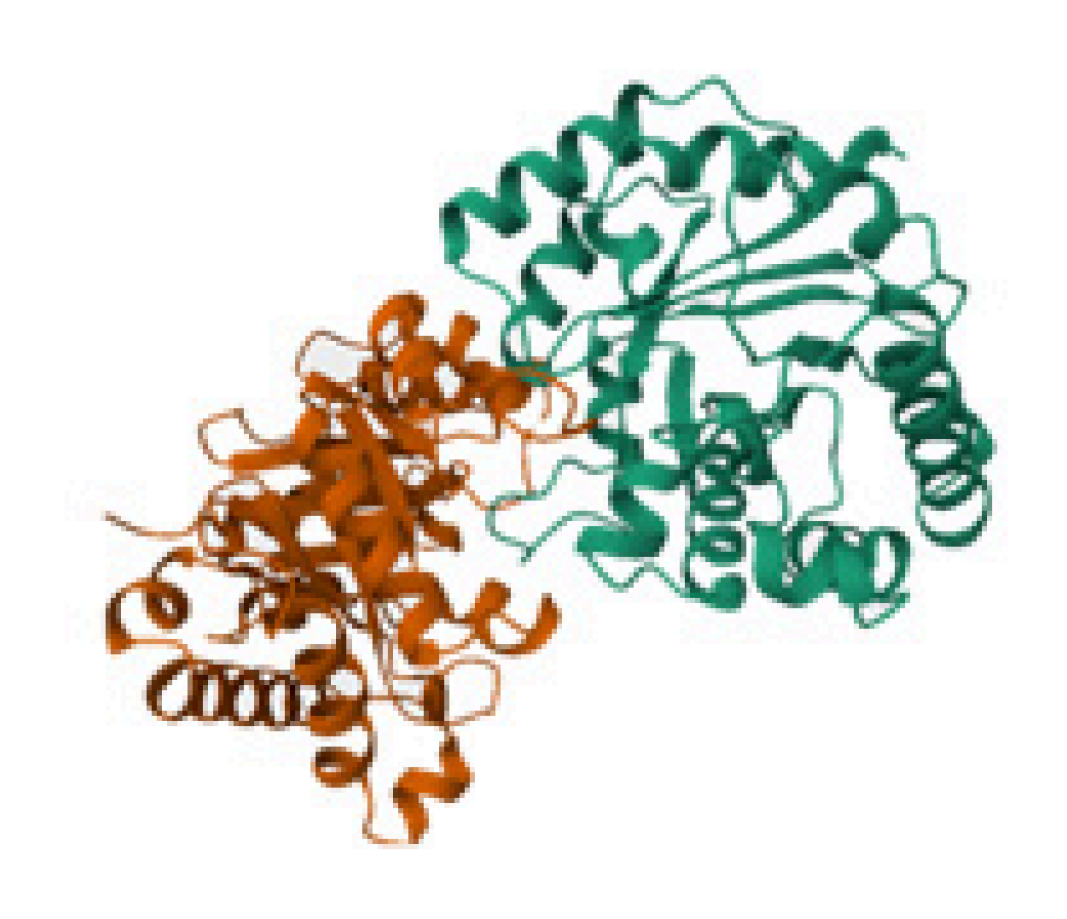		Tri a 31.0101	
Tri a 32	1-cys-peroxiredoxin	24			Tri a 32.0101	
Tri a 33	Serpin	40	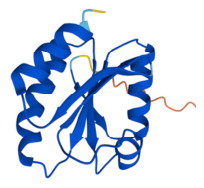		Tri a 33.0101	AA46-68, AA76-92, AA271-293 and AA364-384
Tri a 34	Glyceraldehyde-3-phosphate-dehydrogenase	40–42		5%	Tri a 34.0101	
Tri a 35	Dehydrin	12		5.3%	Tri a 35.0101	
Tri a 36	Low-molecular-weight glutenin	40 kDa	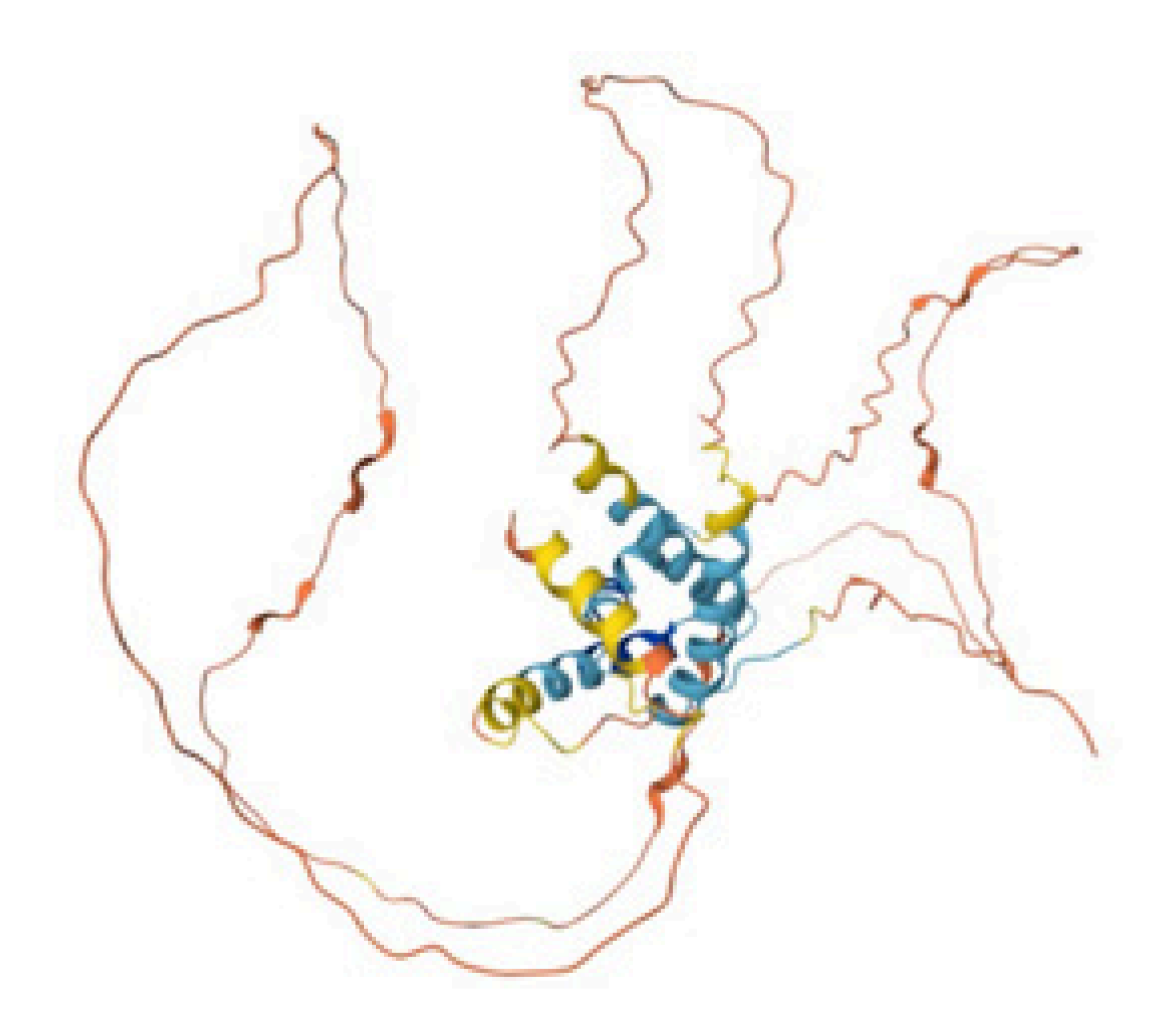		Tri a 36.0101 Tri a 36.0201 Tri a 36.0301	AA37-46, AA39-48, AA47-56, AA211-220, AA231-240, AA263-272, AA269-278, AA29-35, AA65-70, AA77-85, AA224-230, AA246-250, AA254-261, AA265-277 and AA322-337
Tri a 37	Alpha purothionin	12 kDa	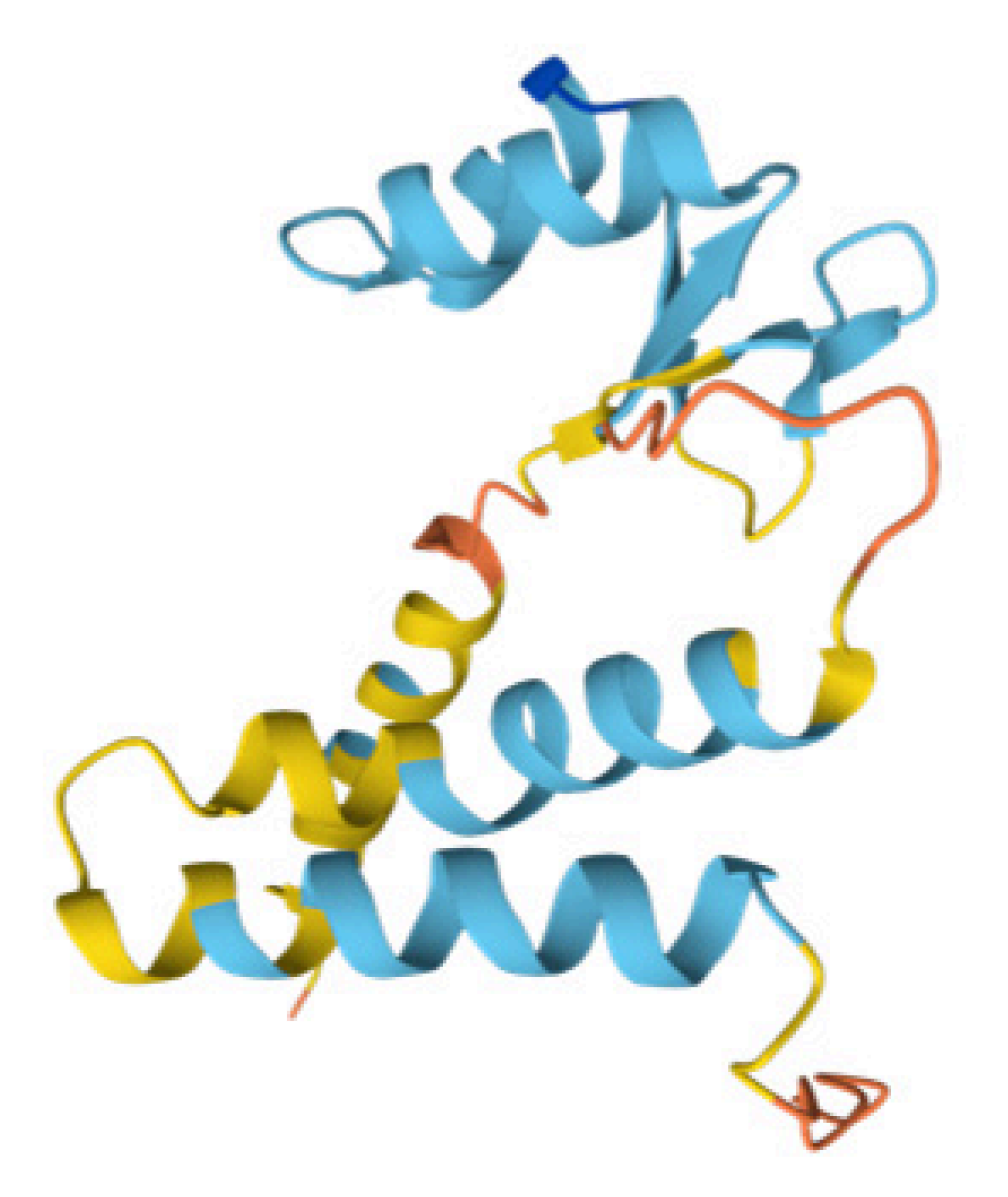		Tri a 37.0101	AA1-30, AA41-70 and AA61-90
Tri a 39	Serine protease inhibitor-like protein	9		18%	Tri a 39.0101	
Tri a 40	Tetrameric alpha-amylase inhibitor CM17	15.96 kDa		8.5%	Tri a 40.0101	
Tri a 41	Mitochondrial ubiquitin ligase activator of NFKB 1 (fragment)			5%	Tri a 41.0101	
Tri a 42	Tapetum determinant 1 (TPD1) fragment			13%	Tri a 42.0101	
Tri a 43	Transcriptional corepressor SCAI	12 kDa		13%	Tri a 43.0101	
Tri a 44	Endosperm transfer cell specific PR60 (nsLTP-like protein)	10 kDa		4%	Tri a 44.0101	
Tri a 45	Transcription elongation factor 1 (EIF1)	10 kDa		13%	Tri a 45.0101	

**Table 5:** The characteristics of tree nut allergens [27,28,78].

Tree Nut Type	Allergens	Biochemical Name	MW (SDS-PAGE)	Structure	Recognition Rate of Allergic Patients	Variants	Epitopes
Almond	Pru du 1	PR-10, Bet v 1-related protein	18 kDa	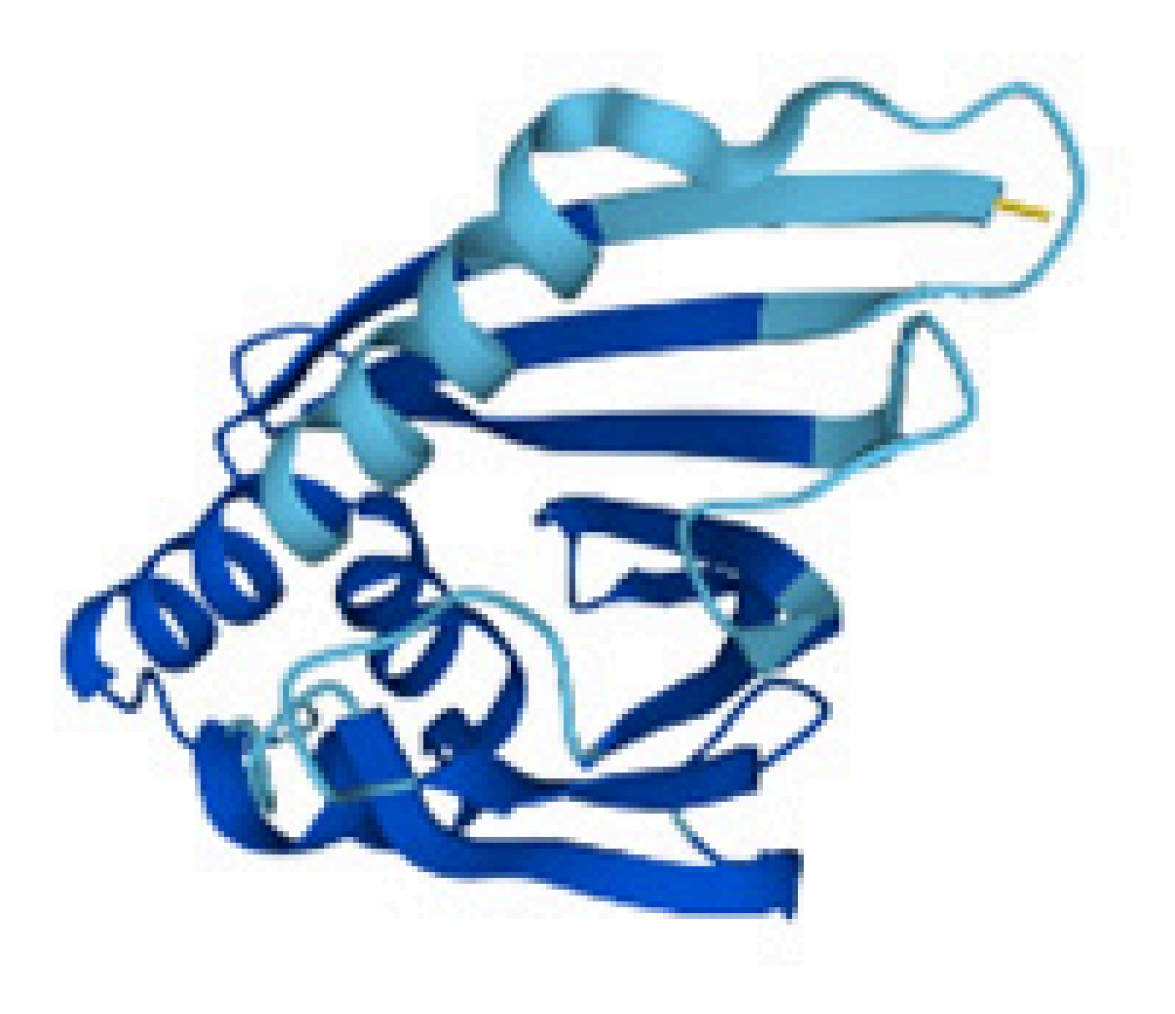	82%		
	Pru du 3	Non-specific lipid transfer protein 1 (nsLTP1)	9 kDa	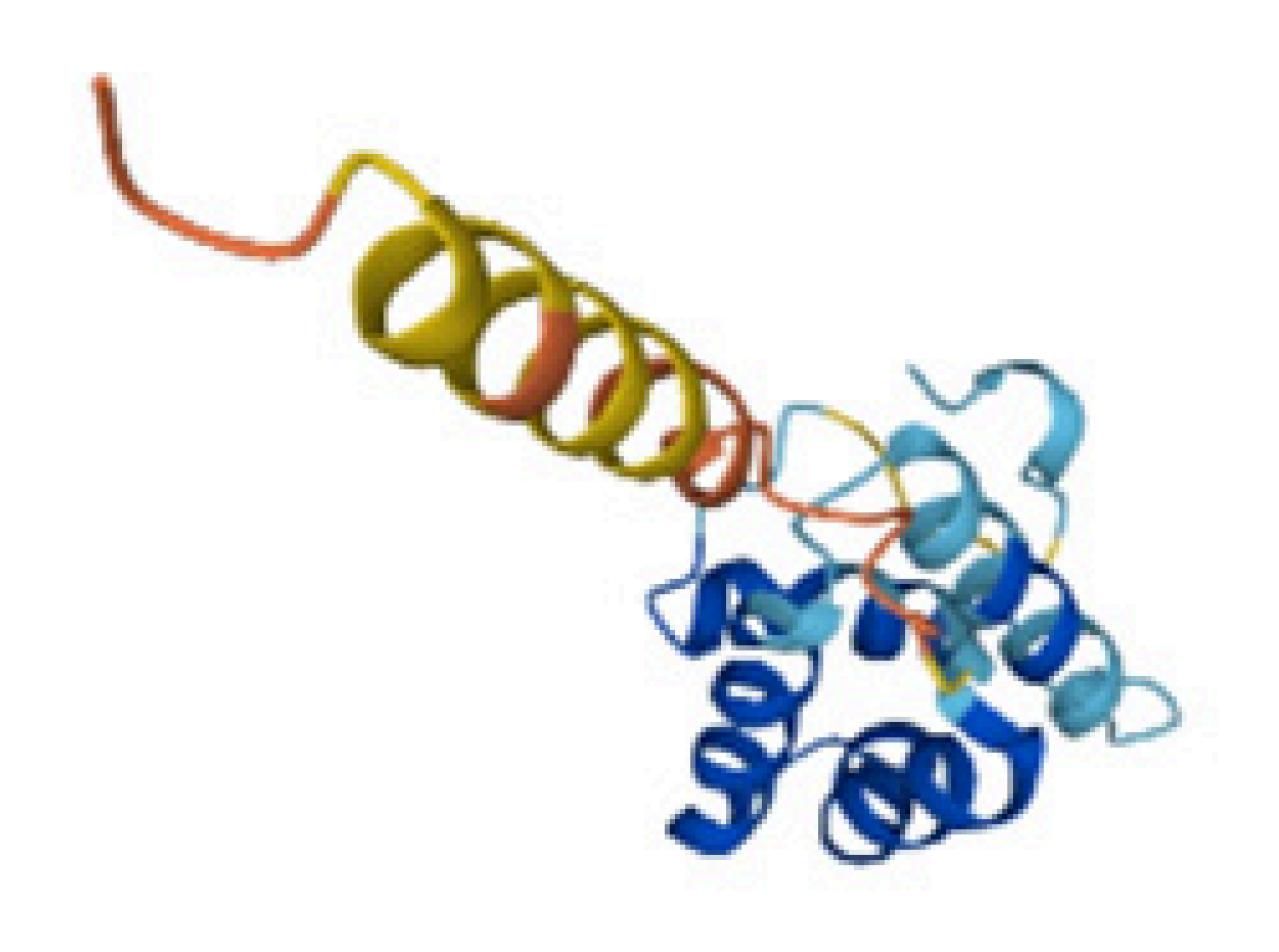	-		
	Pru du 4	Profilin	14 kDa	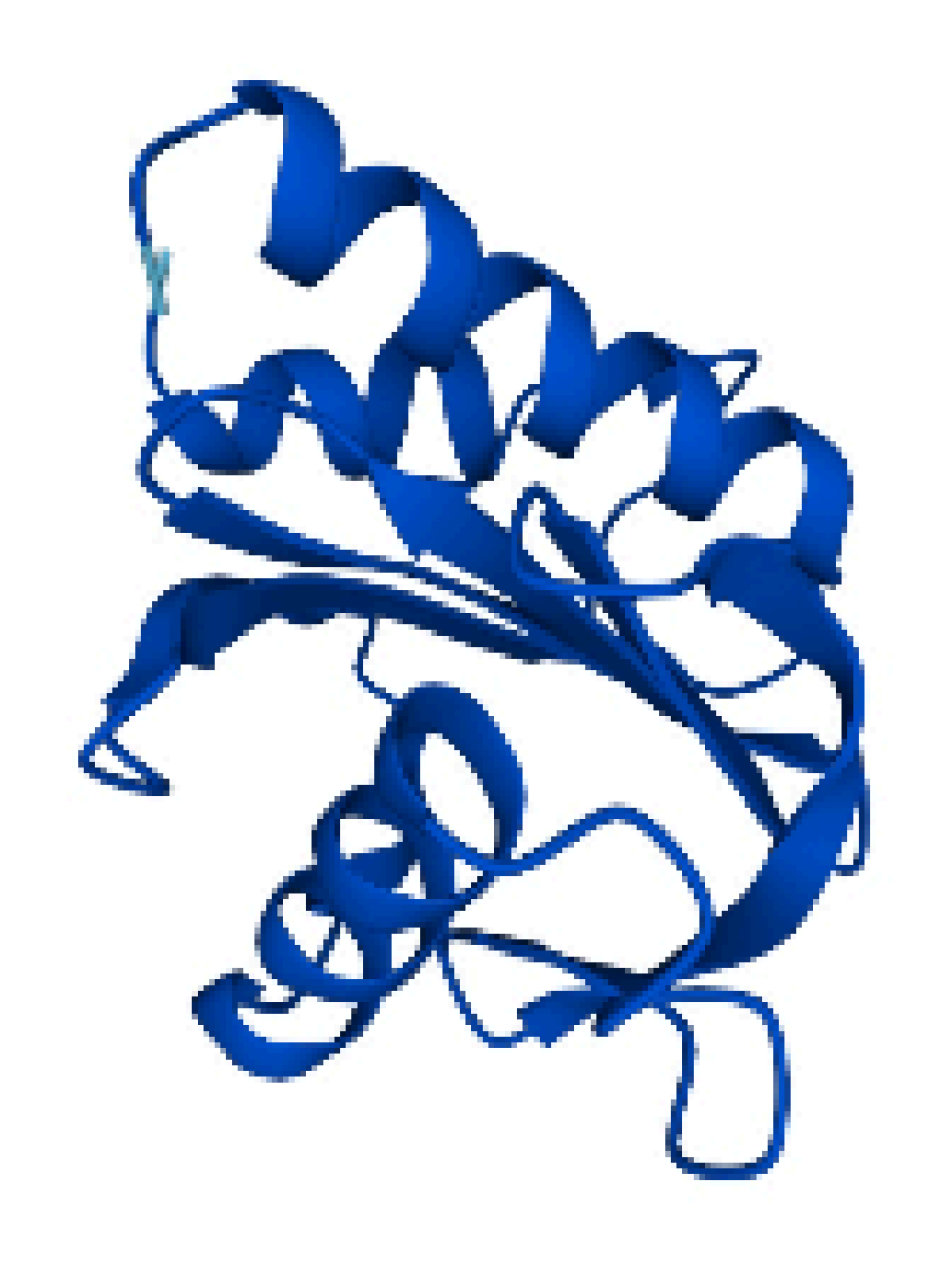	44%		
	Pru du 5	60s acidic ribosomal protein P2	10 kDa	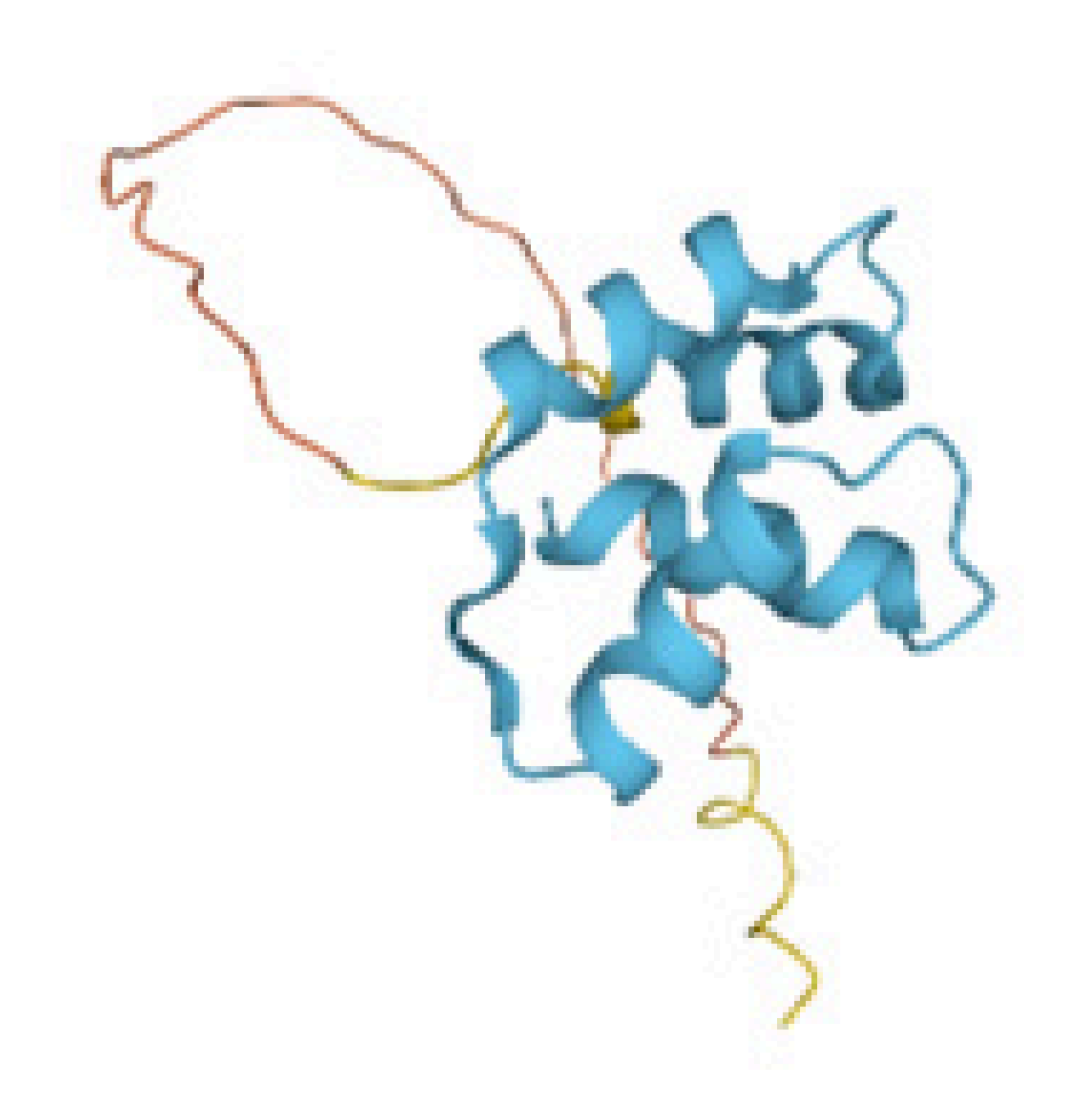	50%		
	Pru du 6	Amandin, 11S globulin, legumin-like protein	360 kDa (hexamer)	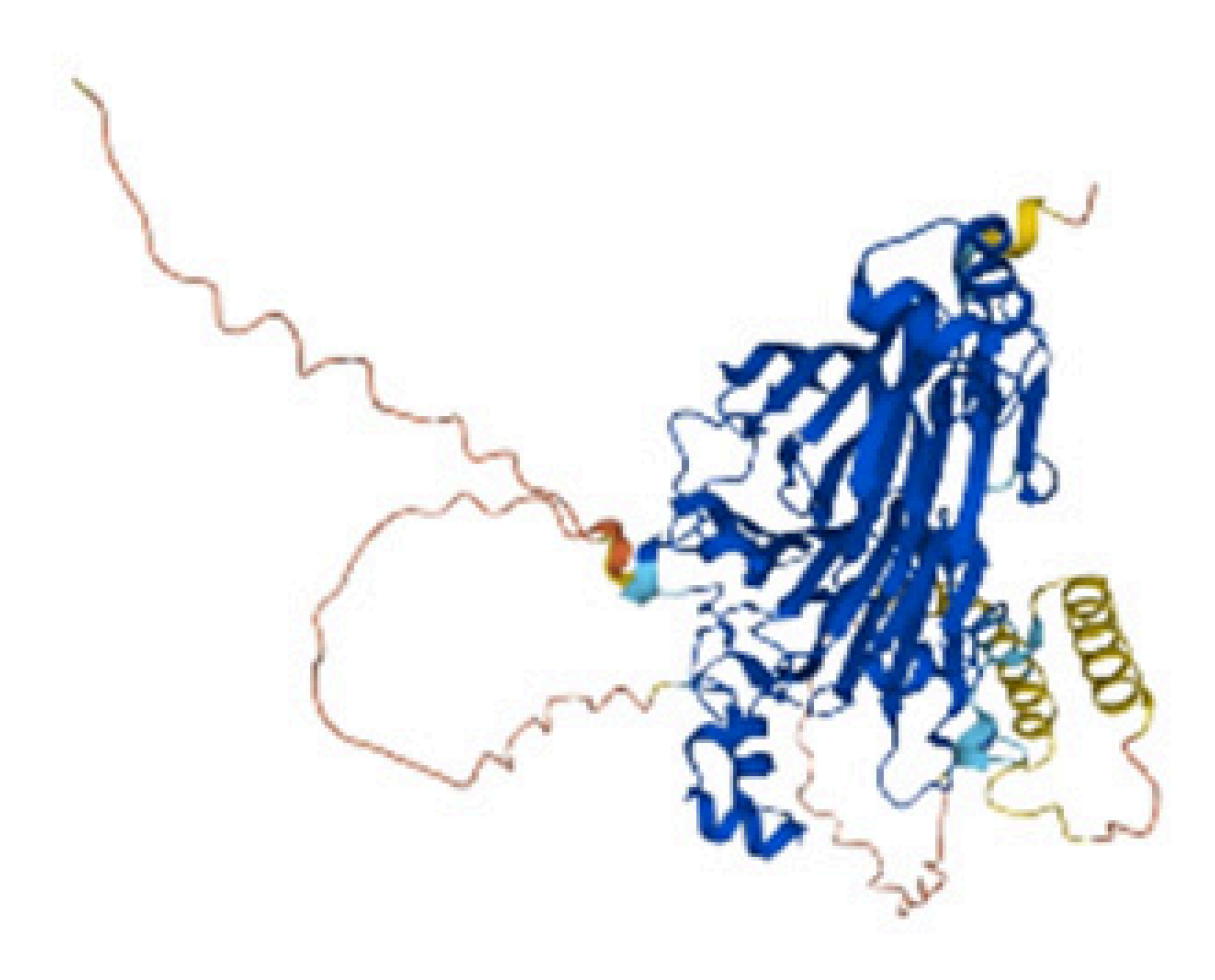	83%	Pru du 6.0101 Pru du 6.0201	AA21-45, AA320-328, AA460-465, AA118-132, AA145-159, AA281-295, AA120-135, AA505-519, AA113-135, AA209-223 and AA465-479
	Pru du 8	Alpha-hairpinin	31 kDa	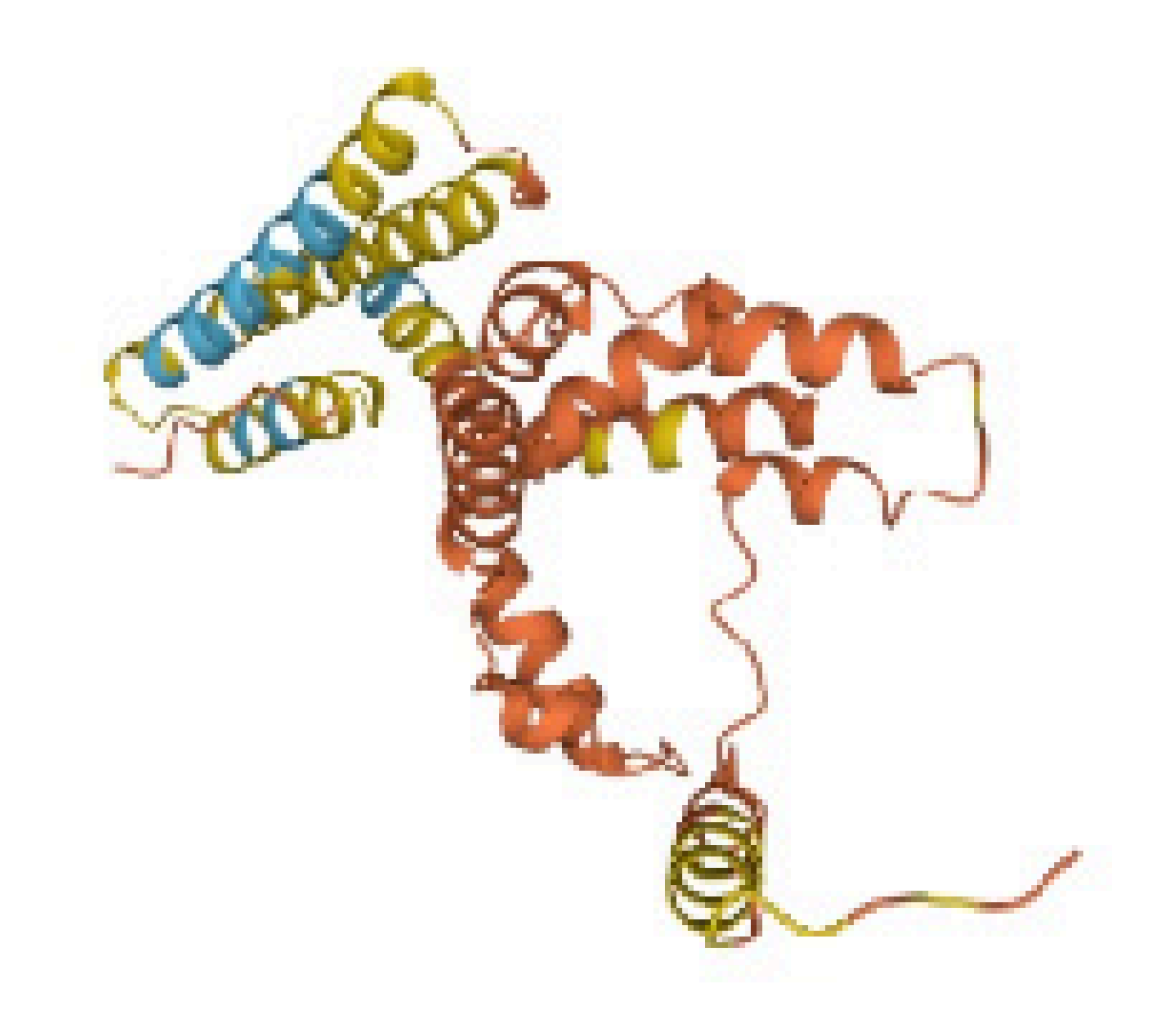	41%		
	Pru du 10	Mandelonitrile lyase 2; Hydroxynitrile lyase	60 kDa	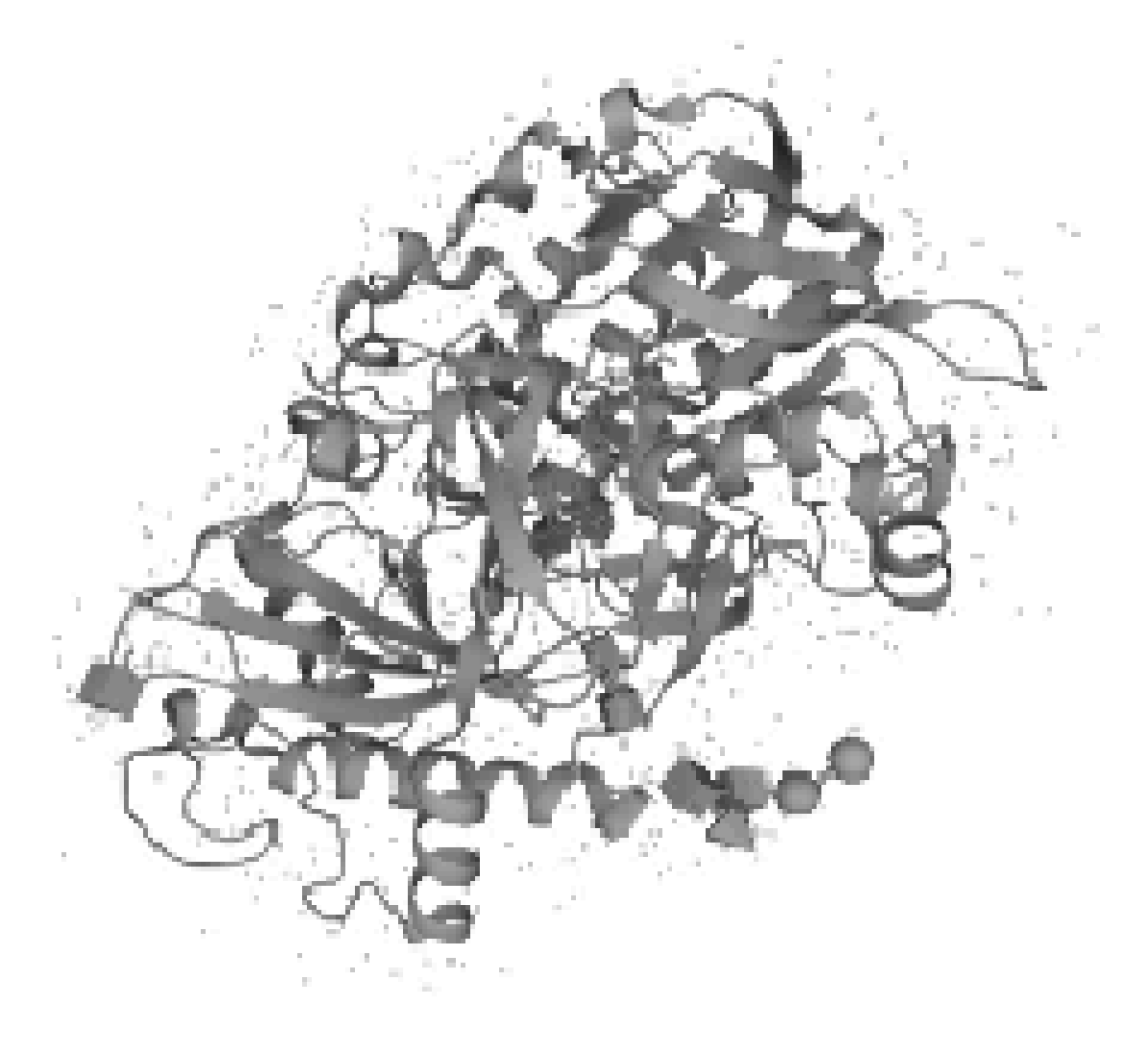	67%		
Hazelnut	Cor a 1	Pathogenesis-related protein, PR-10, Bet v 1 family member	17 kDa	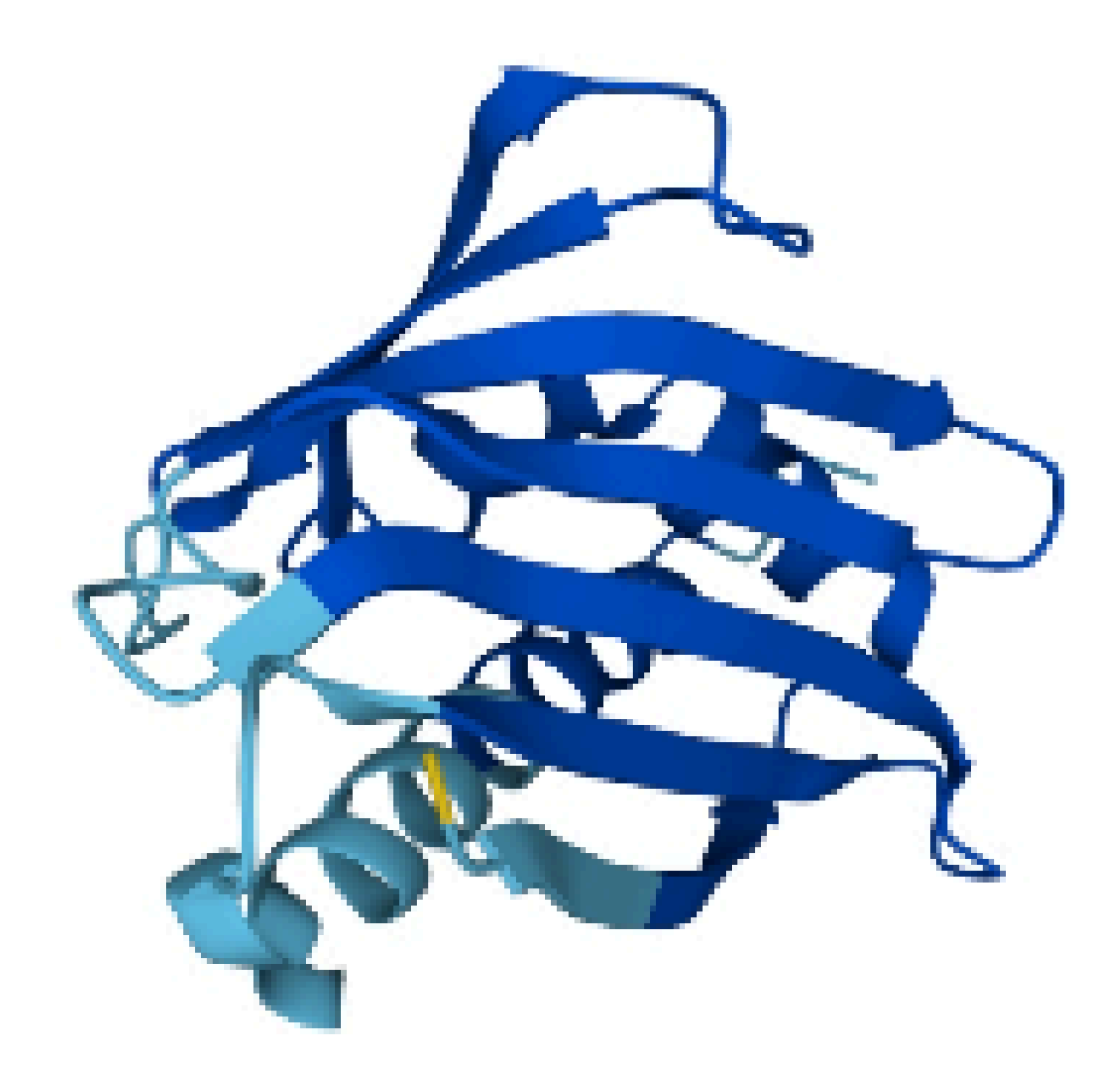	62.7%	Cor a 1.0101~ Cor a 1.0104 Cor a 1.0201 Cor a 1.0301 Cor a 1.0302 Cor a 1.0401~ Cor a 1.0404 Cor a 1.0501 Cor a 1.0601 Cor a 1.0701 Cor a 1.0801	
	Cor a 2	Profilin	14 kDa	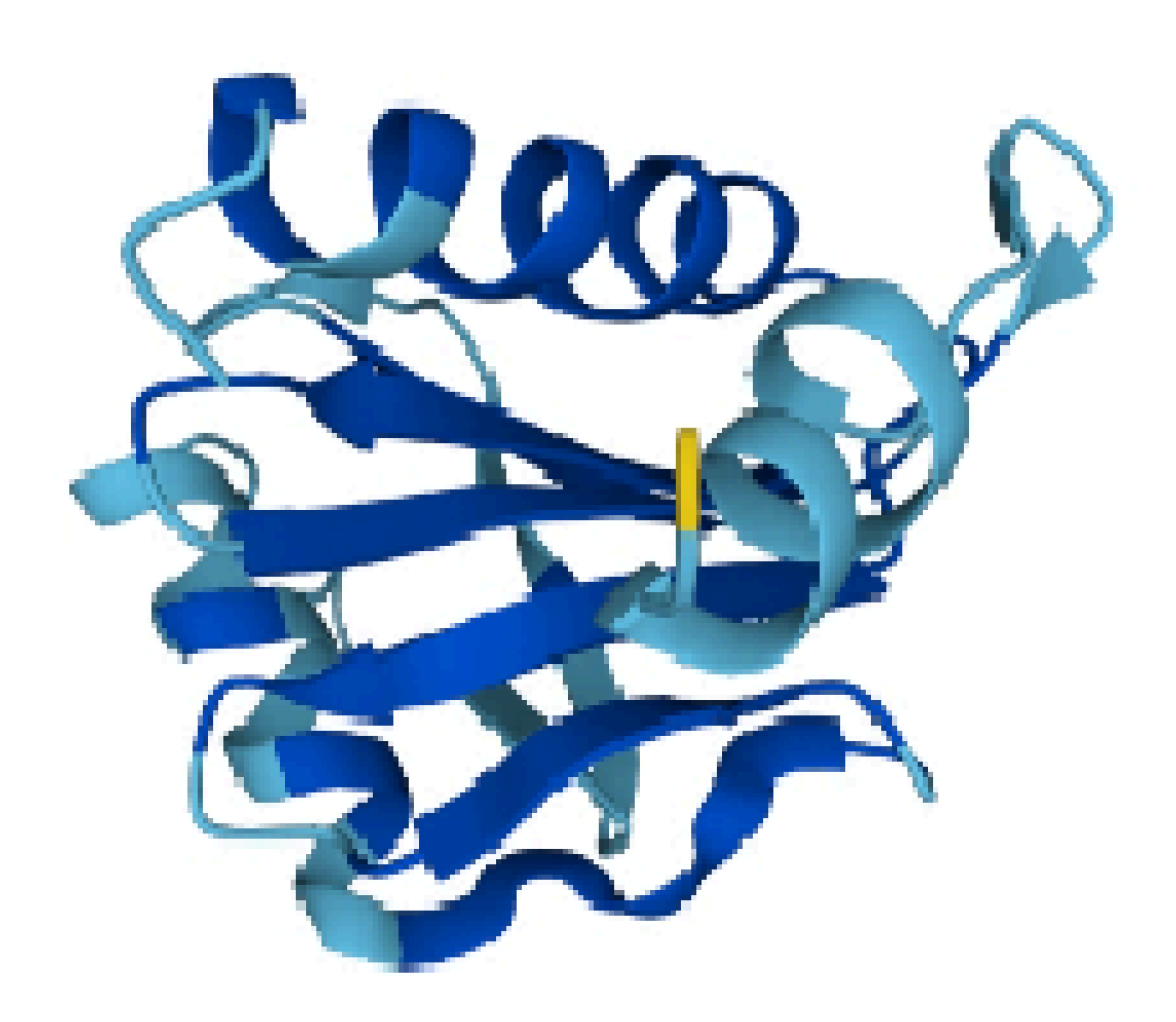	42%	Cor a 2.0101 Cor a 2.0102	
	Cor a 6	Isoflavone reductase homologue	35 kDa	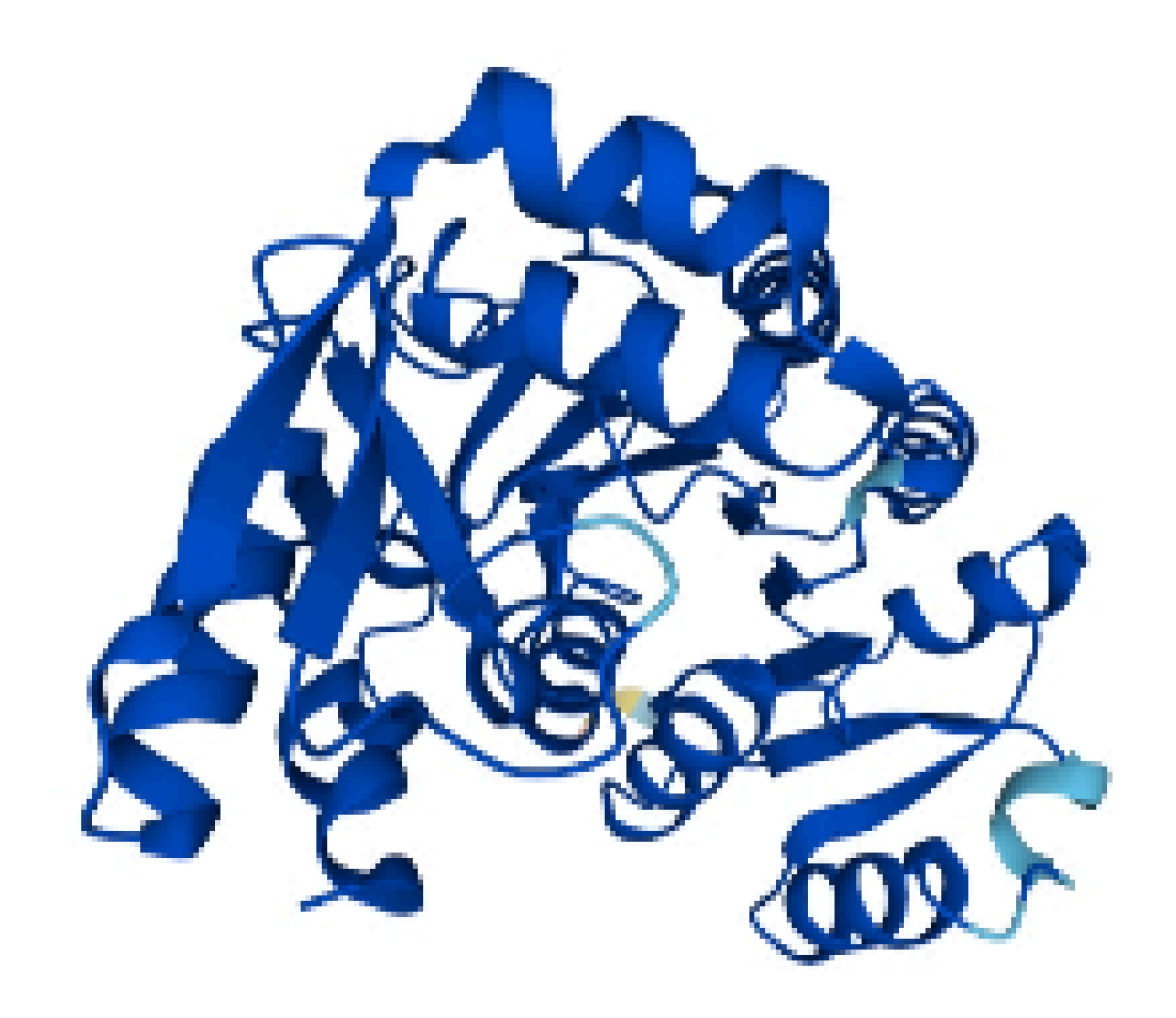	-		
	Cor a 8	Non-specific lipid transfer protein type 1	9 kDa	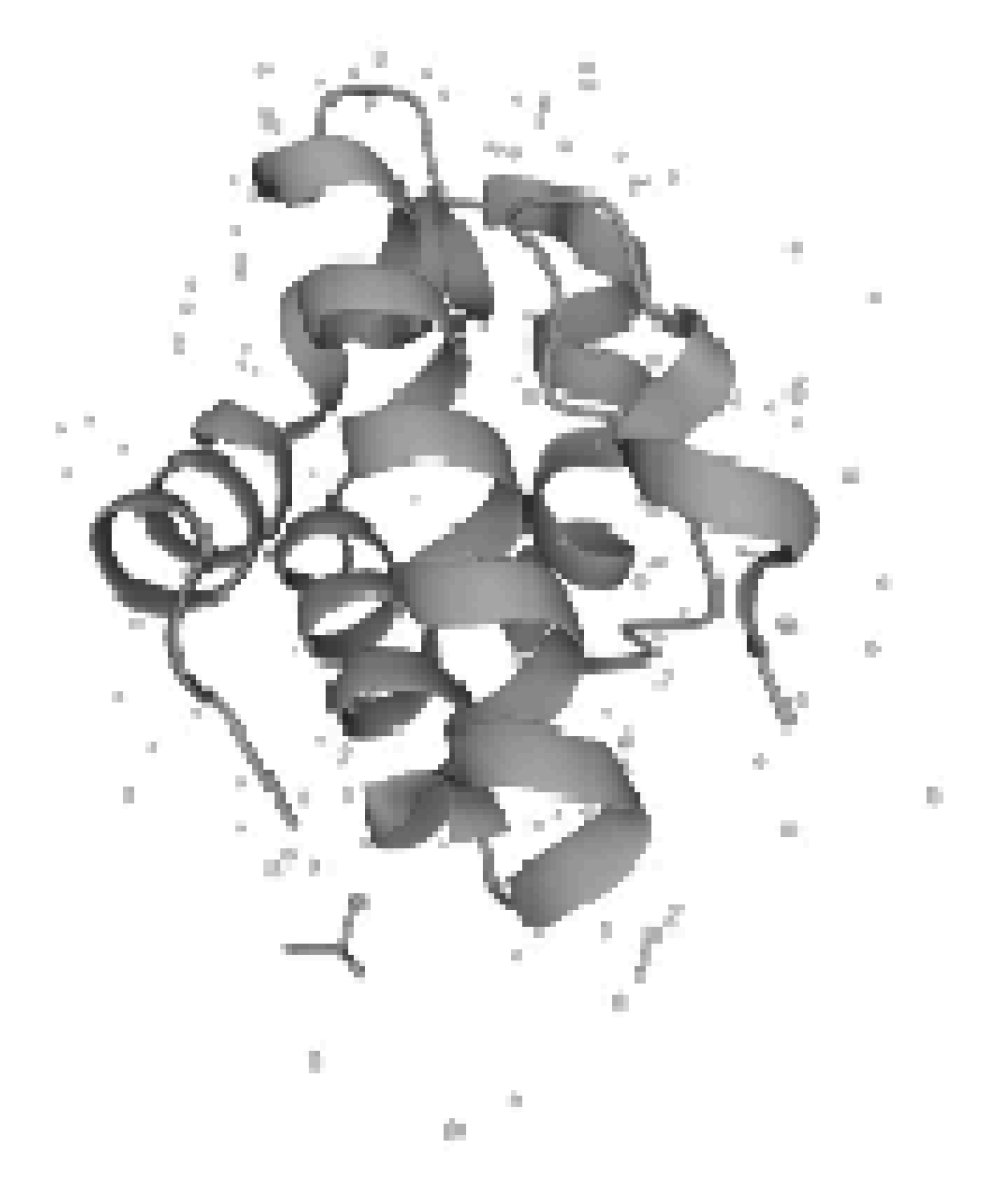	31.3%		
	Cor a 9	11S seed storage globulin (legumin-like)	40 kDa	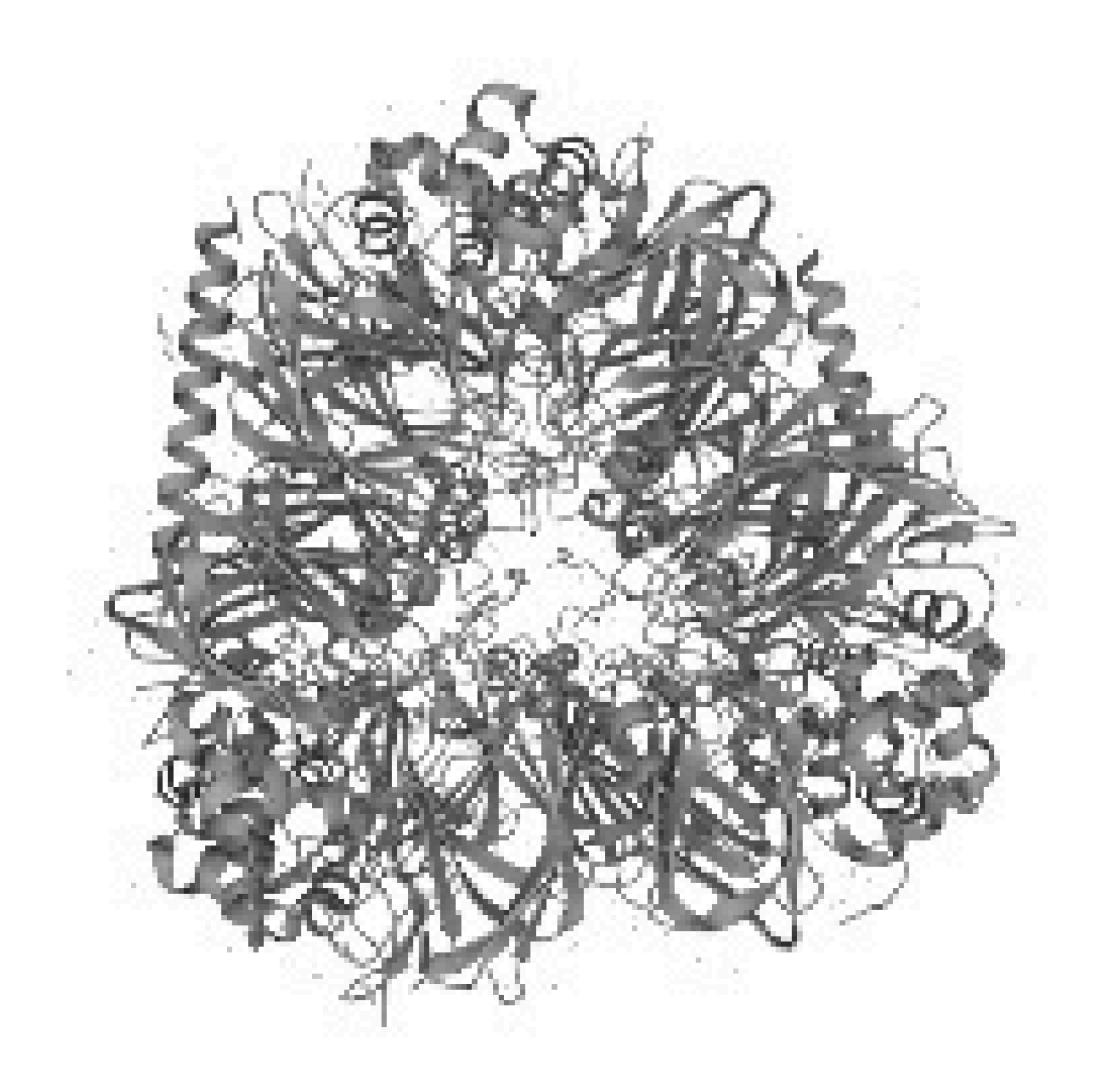	86%		AA250-264, AA1-15, AA185-199, AA201-215, AA241-255 and AA281-295
	Cor a 10	Luminal binding protein	70 kDa	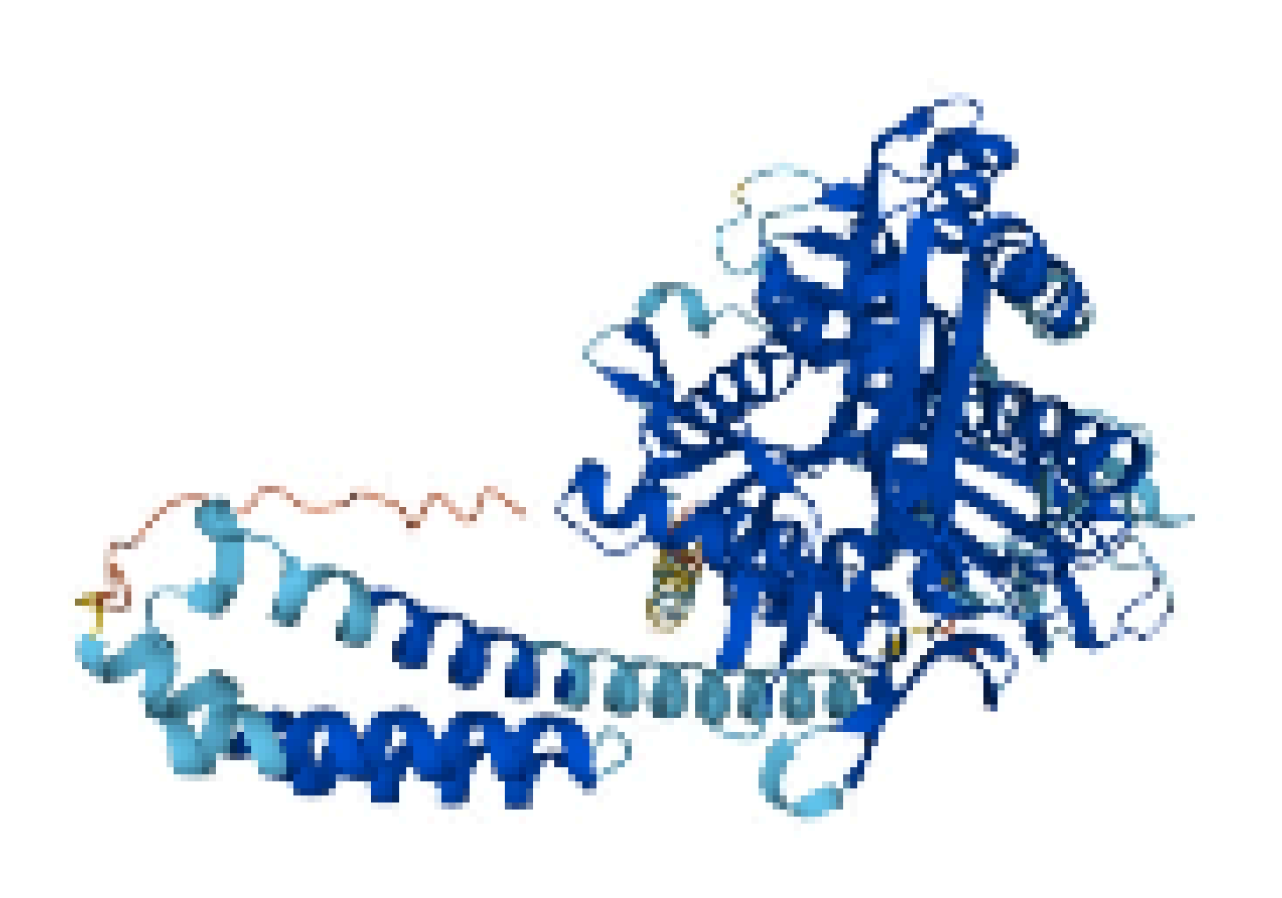			
	Cor a 11	7S seed storage globulin (vicilin-like)	48 kDa	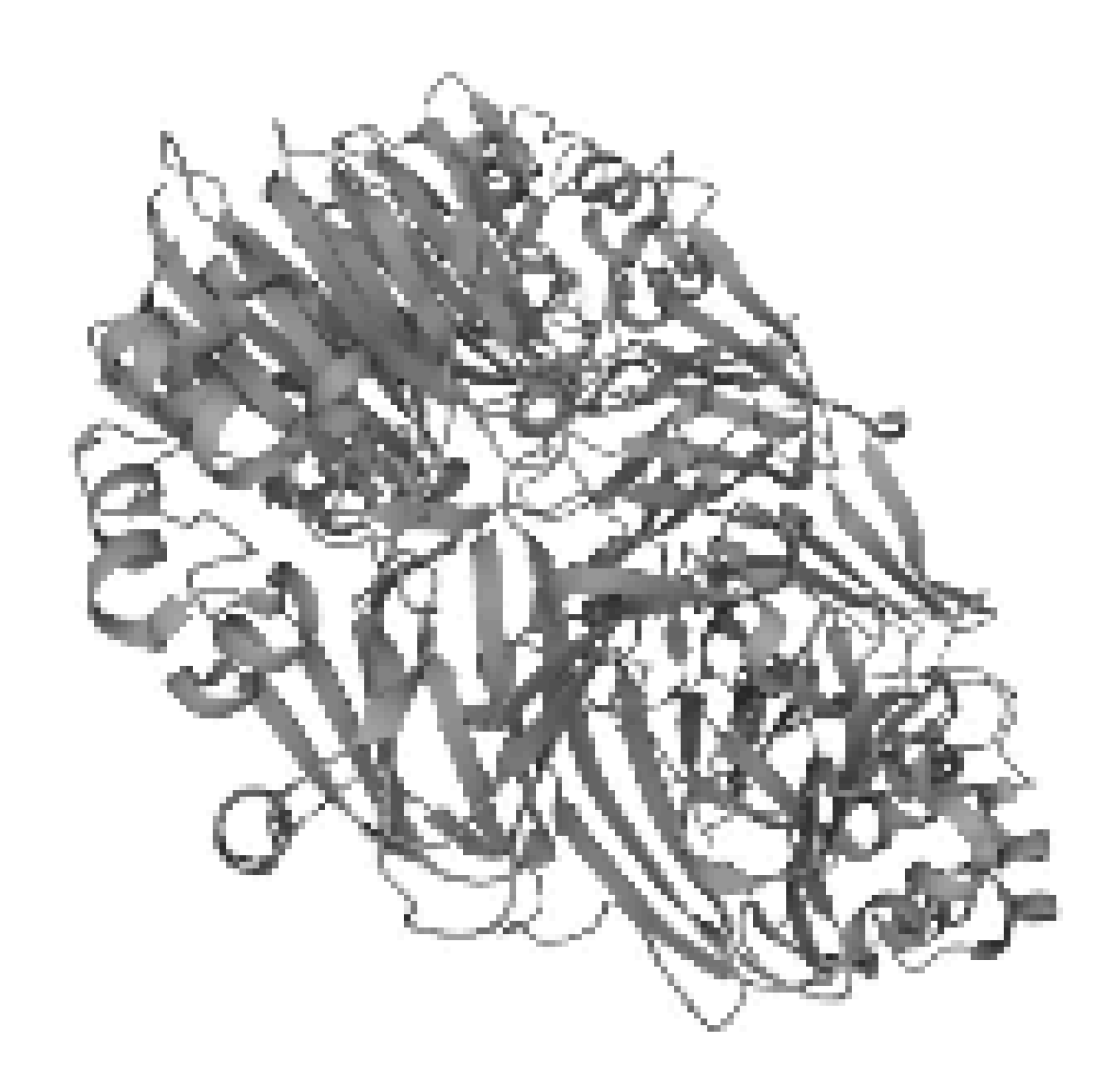	47%	Cor a 11.0101 Cor a 11.0102	
	Cor a 12	17 kDa oleosin	17 kDa	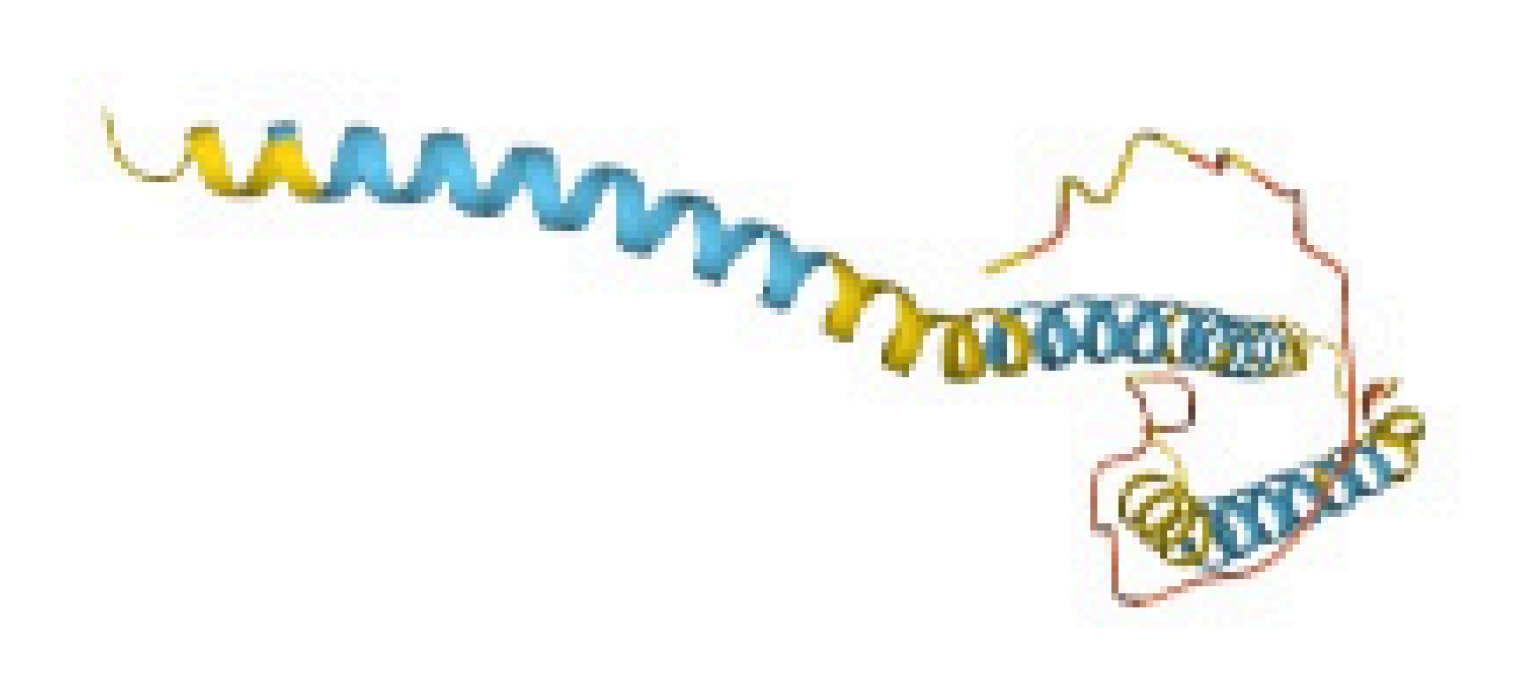	63%		
	Cor a 13	14–16 kDa oleosin	14–16 kDa	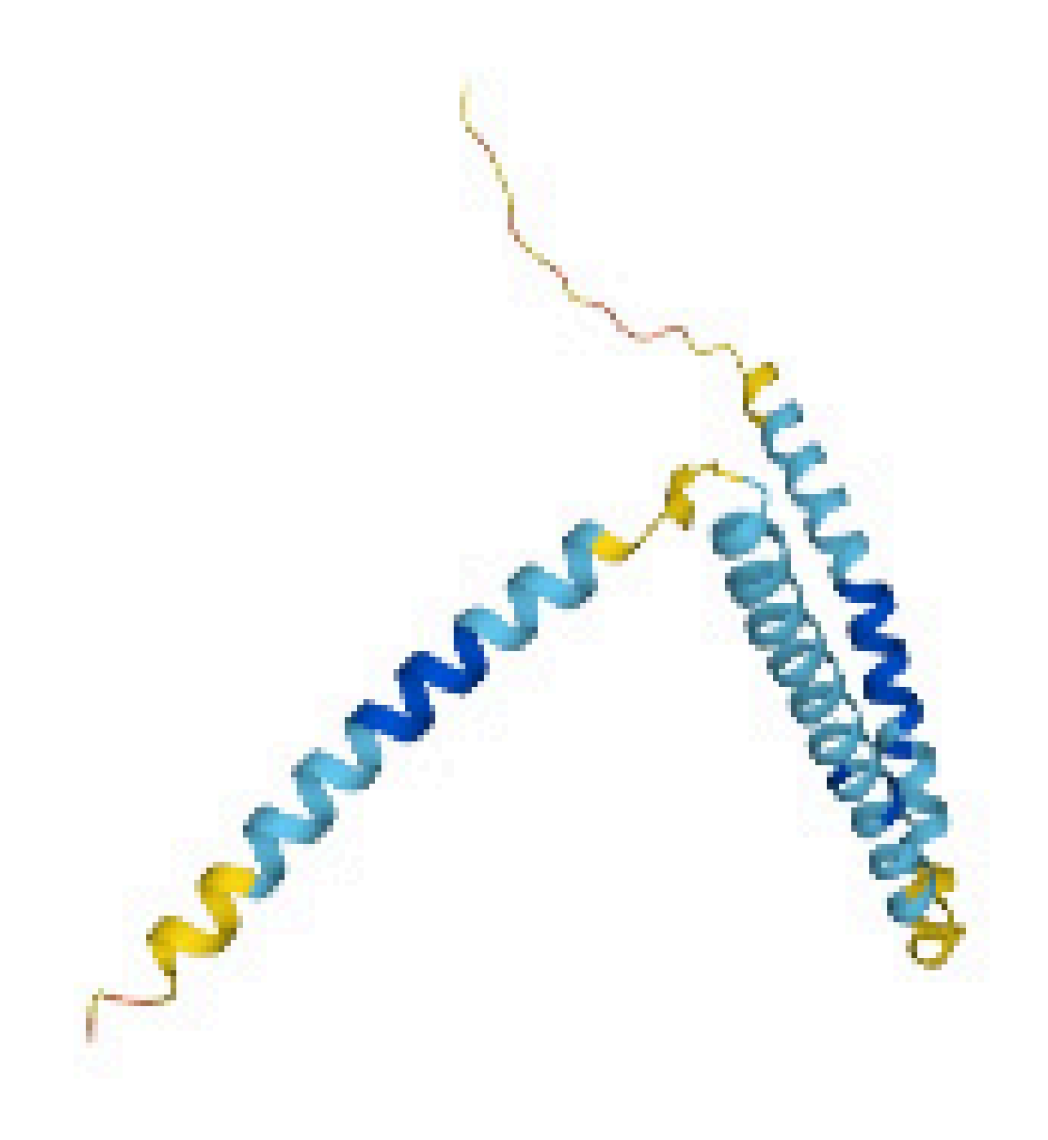	63%		
	Cor a 14	2S albumin	10 kDa reducing	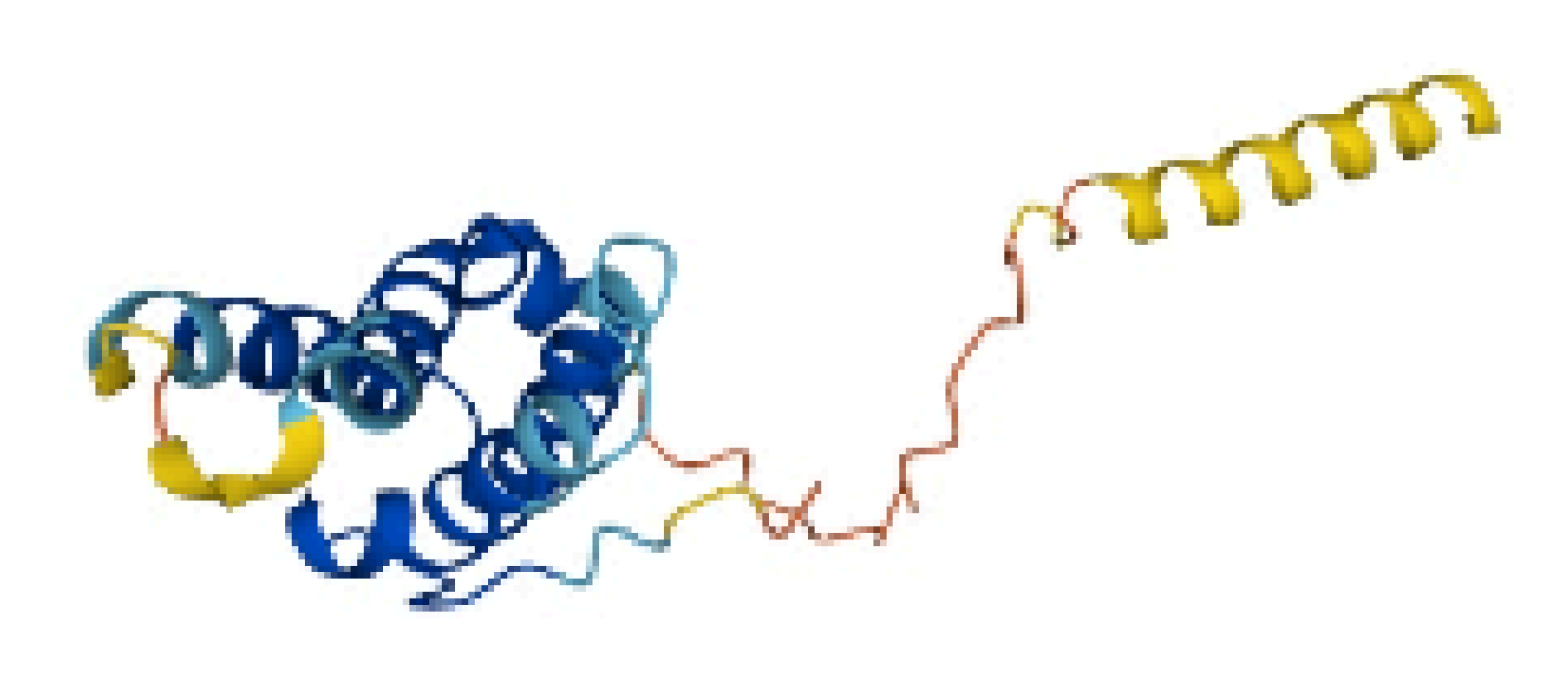	33%		
	Cor a 15	Oleosin	17 kDa	-	65%		
	Cor a 16	7S globulin seed storage protein (vicilin) containing N-terminal alpha-hairpinin (vicilin_N) peptides	6–8 kDa and 47.5 kDa	-	53%		
Walnut	Jug r 1	2S albumin seed storage protein	15–16 kDa	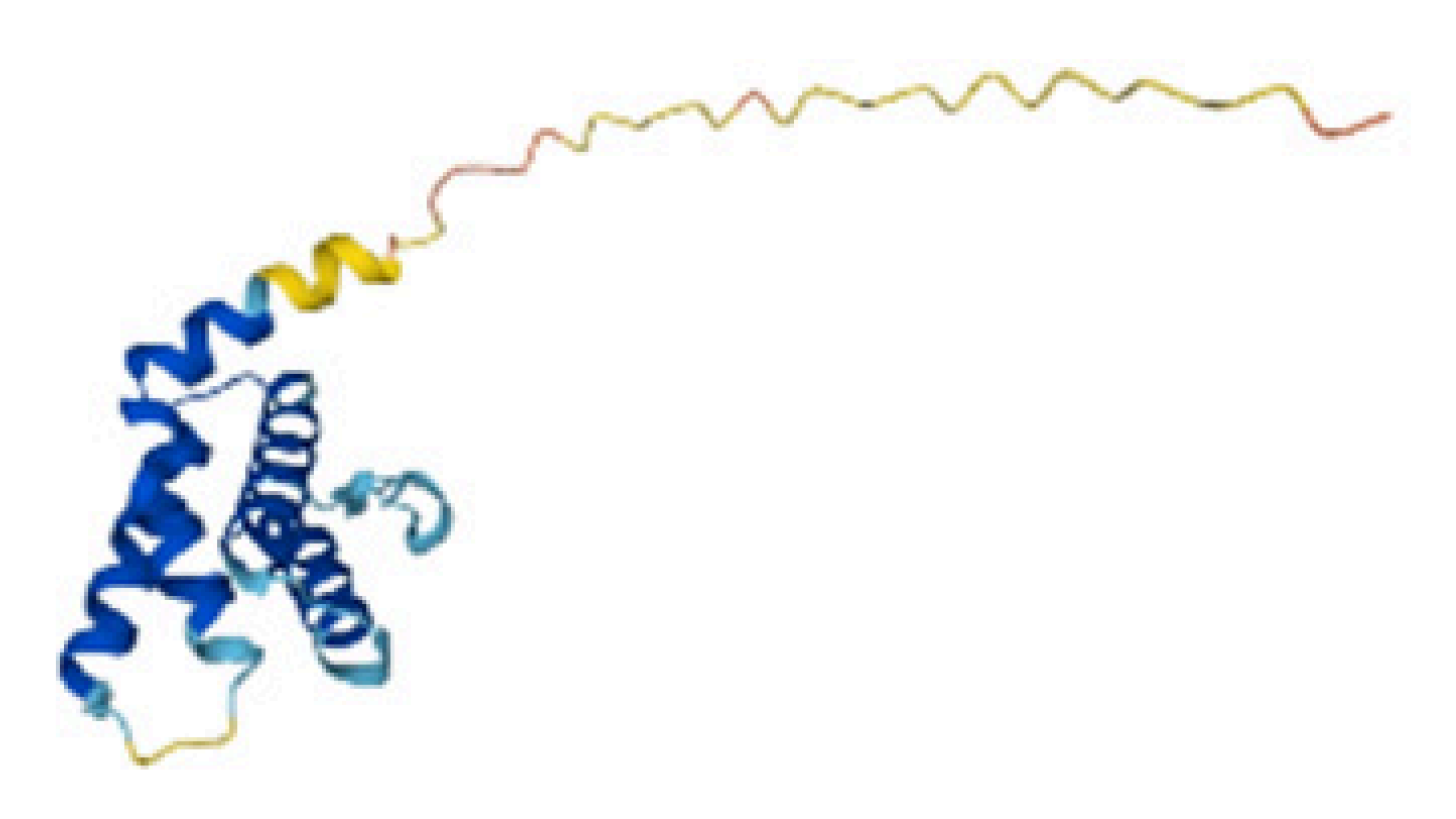	75%		AA6-11, AA42-50, AA72-80, AA102-113, AA16-30, AA125-139, AA29-34, AA125-136, AA65-73 and AA95-117
	Jug r 2	Vicilin (7S globulin) seed storage protein	44 kDa	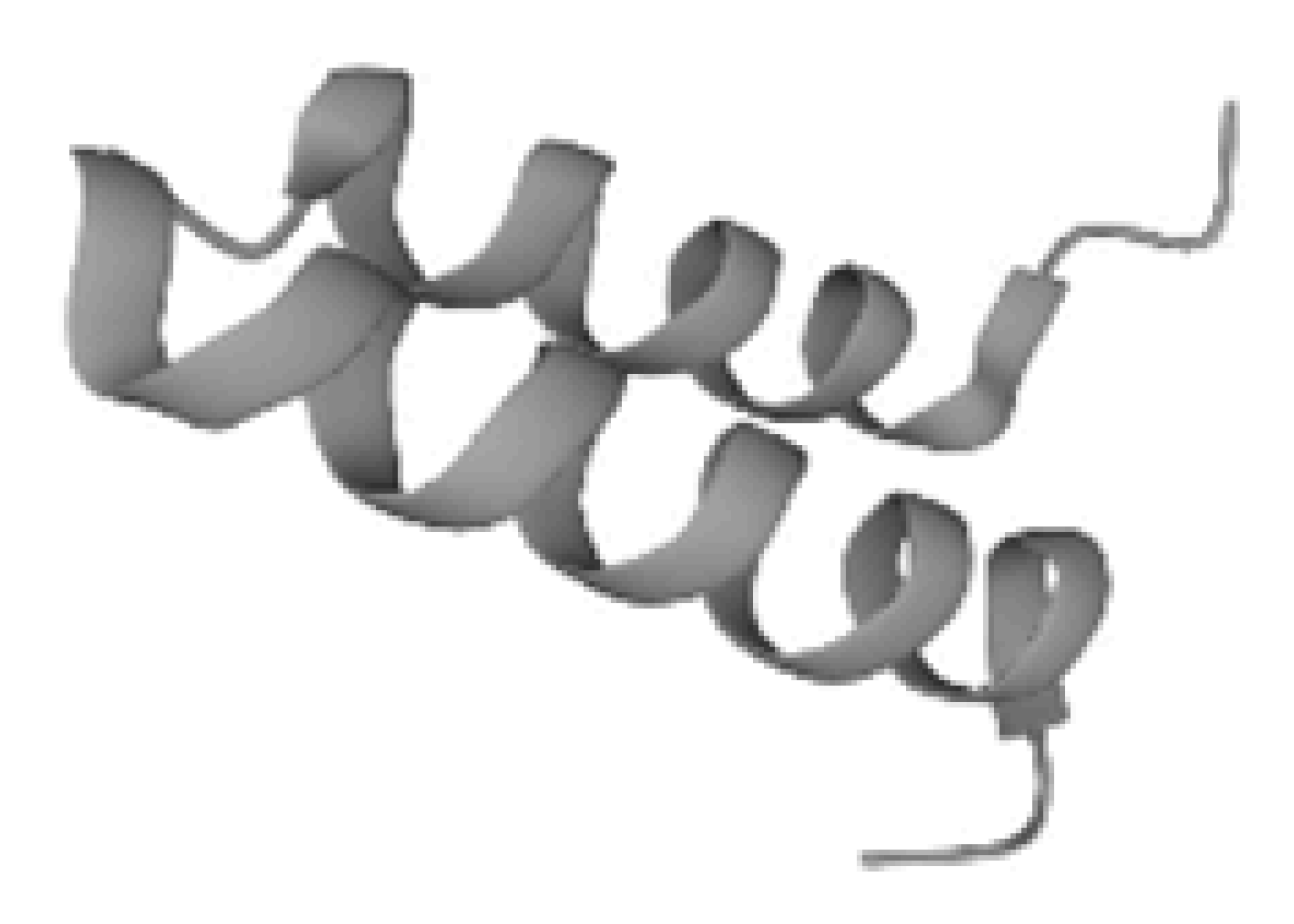	60%	Jug r 2.0101 Jug r 2.0102	
	Jug r 3	Non-specific lipid transfer protein type 1 (nsLTP1)	9 kDa	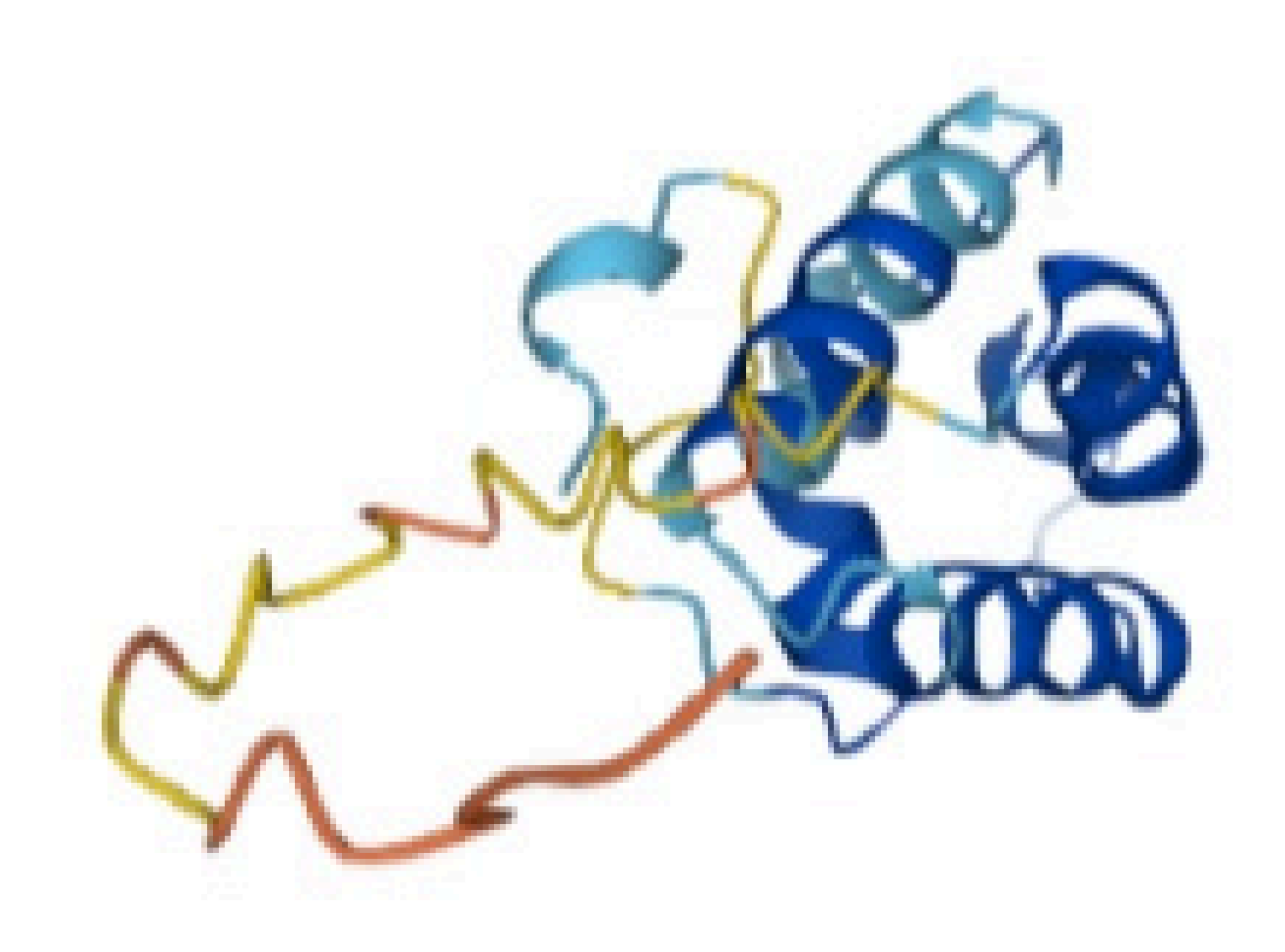	80%		
	Jug r 4	11S globulin seed storage protein	58.1 kDa	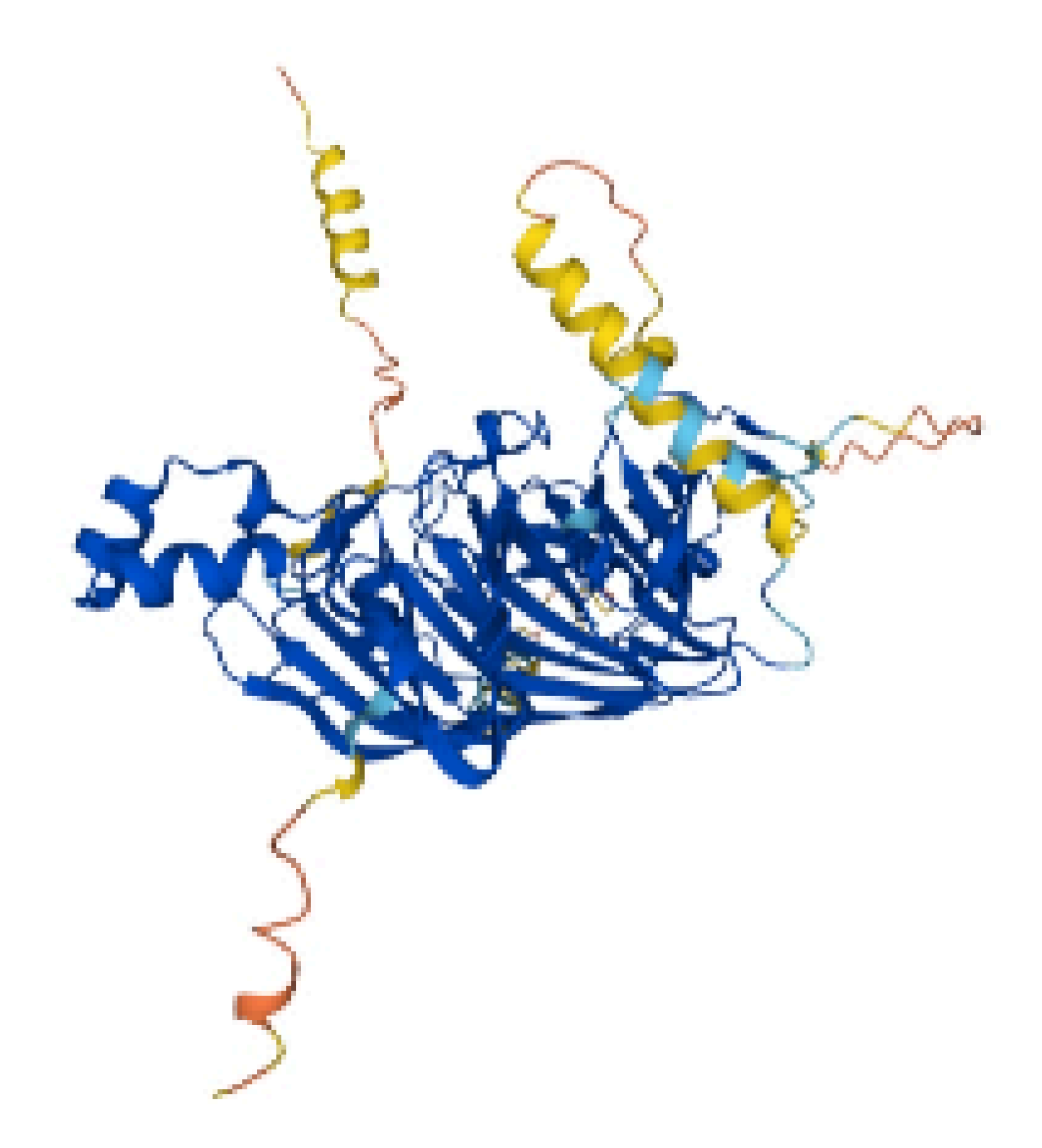	65%		AA1-15, AA233-247, AA253-271, AA281-295 and AA425-439
	Jug r 5	PR-10	20 kDa	No structure information			
	Jug r 6	7S globulin seed storage protein, vicilin-like protein	47 kDa	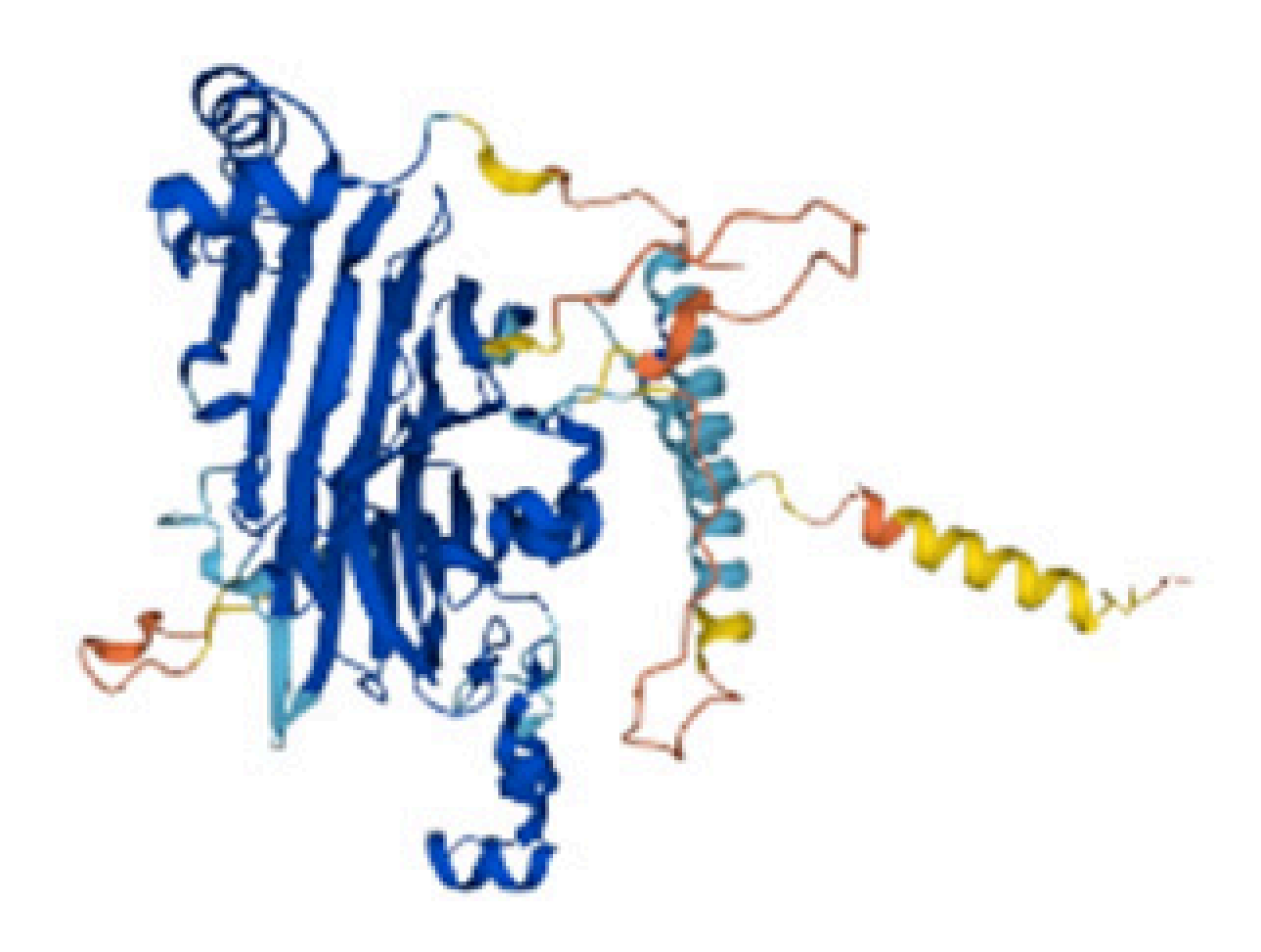	26%		
	Jug r 7	Profilin	13 kDa	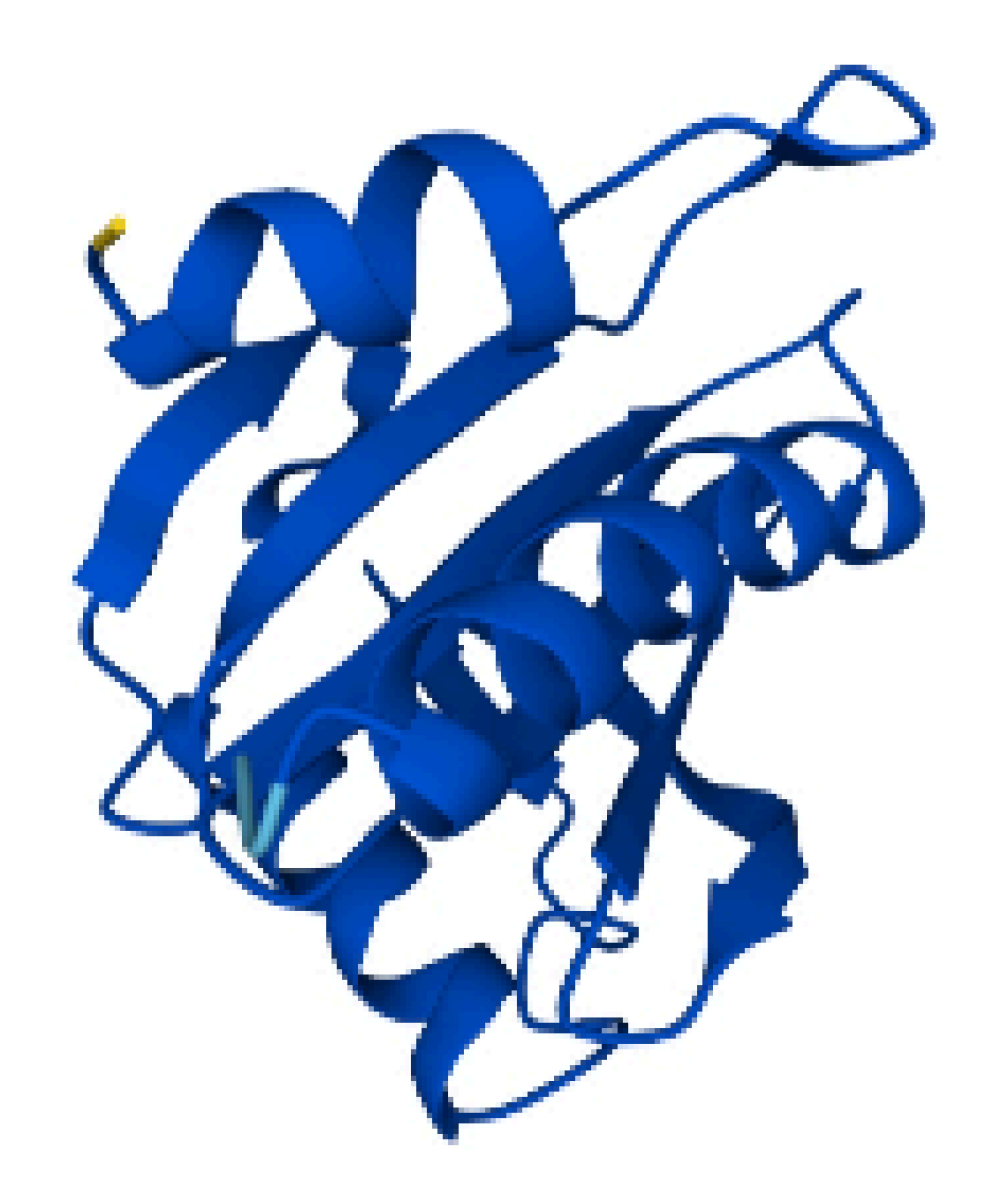	22%		
	Jug r 8	Non-specific lipid transfer protein type 2(ns-LTP-2)	9 kDa	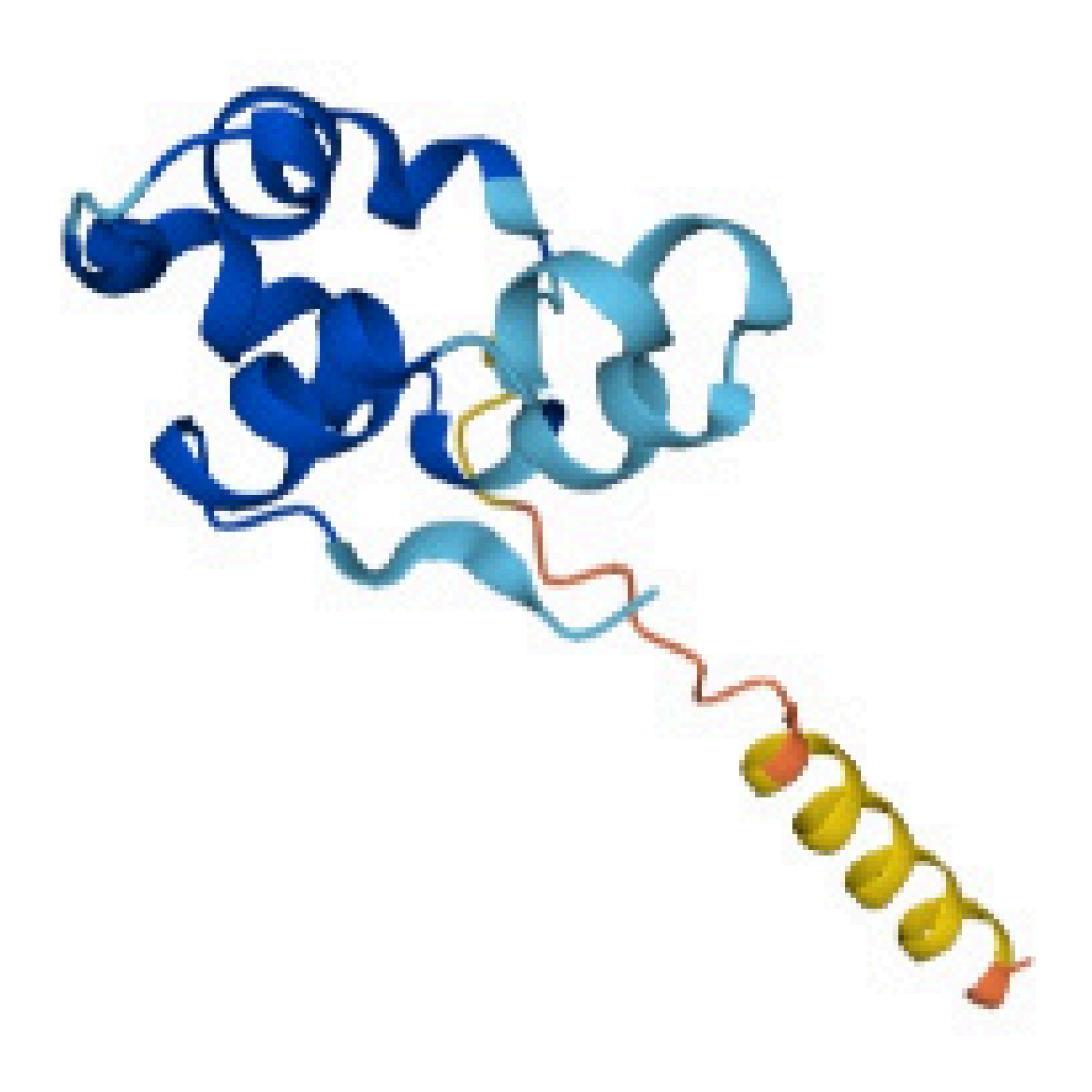	40%	Jug r 8.0101 Jug r 8.0201	
	Jug r 9	Phospholipase D alpha 1	92 kDa		24%		
Cashew	Ana o 1	Vicilin-like protein	50 kDa	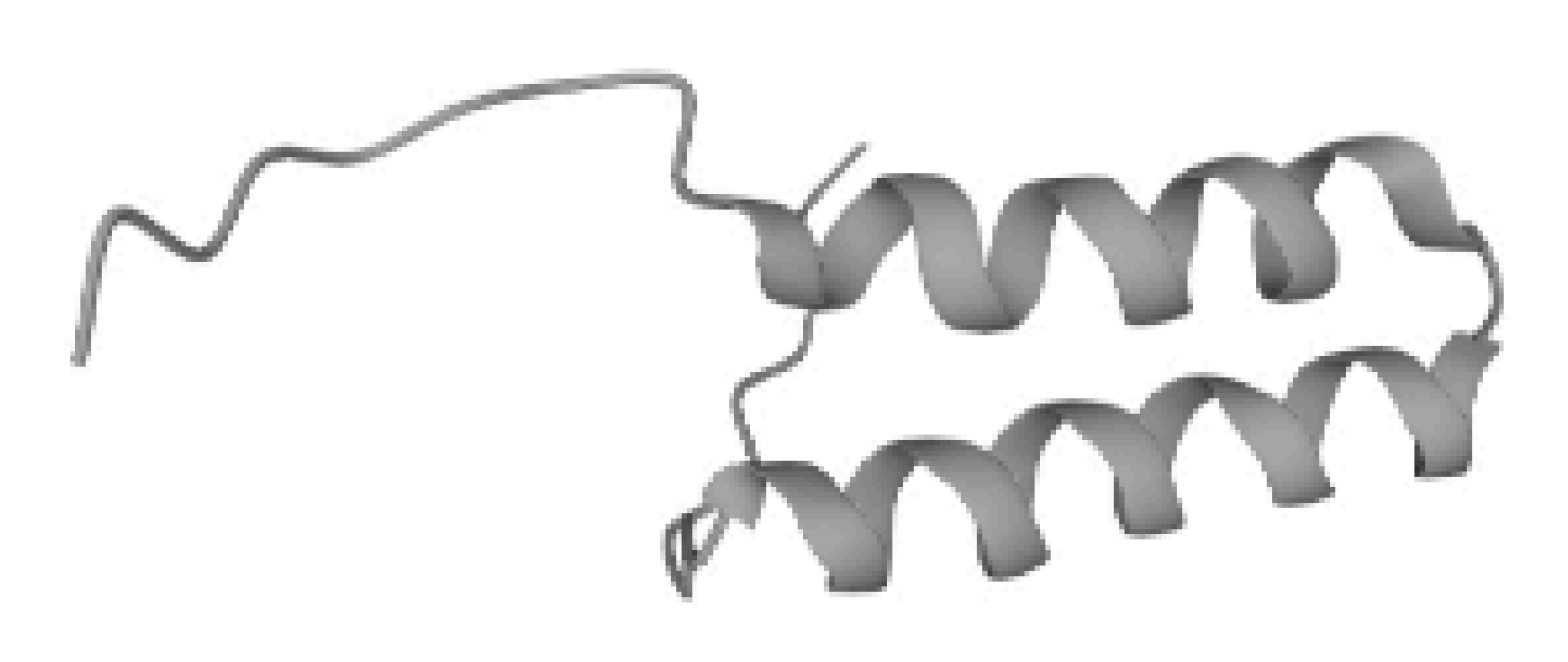	50%	Ana o 1.0101 Ana o 1.0102	AA1-15, AA41-55, AA49-53, AA57-71, AA65-79, AA145-159, AA337-351, AA393-407, AA409-423, AA433-447 and AA521-535
	Ana o 2	Legumin-like protein	55 kDa	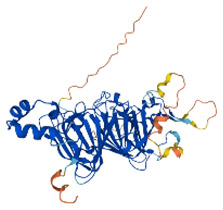	62%		AA257-271, AA1-15, AA9-23, AA15-29, AA17-31, AA89-103, AA105-119, AA113-127, AA121-153, AA137-151, AA169-183, AA185-199, AA217-231, AA233-247, AA241-255, AA257-271, AA313-327, AA329-343, AA337-351, AA277-291, AA385-399, AA417-431, AA425-439, AA15-29, AA105-119, AA185-199, AA233-255, AA257-271 and AA425-439
	Ana o 3	2S albumin	14 kDa	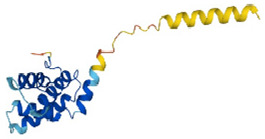	81%		AA33-44, AA39-50, AA42-53, AA45-56, AA48-59, AA54-65, AA57-68, AA66-77, AA69-80, AA72-83, AA75-86, AA78-89, AA99-110, AA102-113, AA105-116, AA123-134, AA39-54, AA10-24, AA13-27, AA16-30, AA40-54, AA49-63, AA55-69, AA76-90, AA85-99, AA88-102, AA94-108, AA112-126, AA121-135, AA35-44, AA57-68, AA72-83, AA102-113, AA19-51, AA52-68, AA69-84 and AA94-111
Pecan	Car i 1	2S albumin seed storage protein	16 kDa	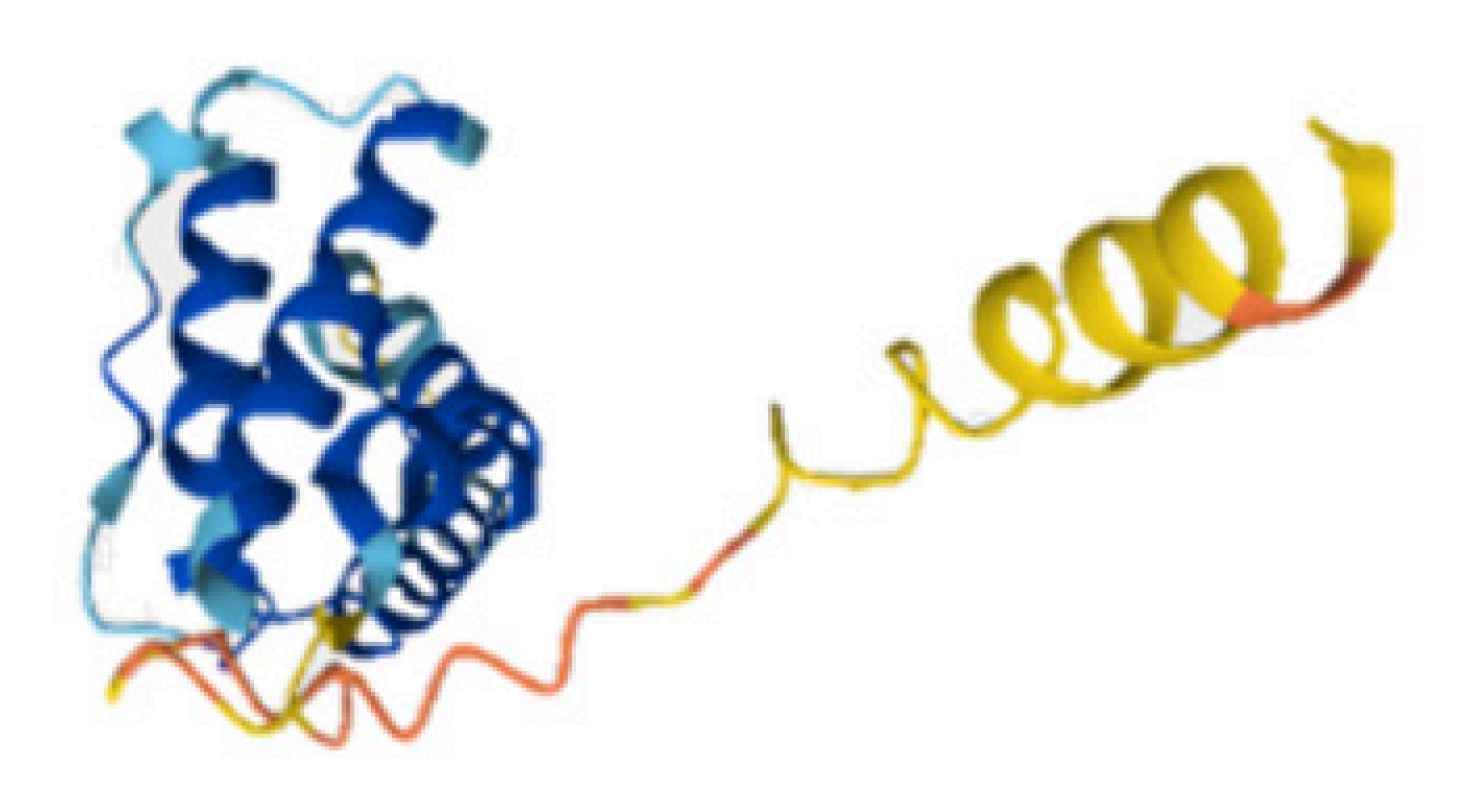	79%		AA68-82, AA4-15, AA7-18, AA25-36, AA28-39, AA31-42, AA34-45, AA37-48, AA43-54, AA46-57, AA49-60, AA52-63, AA55-66, AA58-69, AA67-78, AA70-81, AA73-84, AA76-87, AA97-108, AA100-111, AA103-114, AA106-117, AA109-120, AA130-141 and AA133-143
	Car i 2	Vicilin-like seed storage protein; 7S globulin	55 kDa	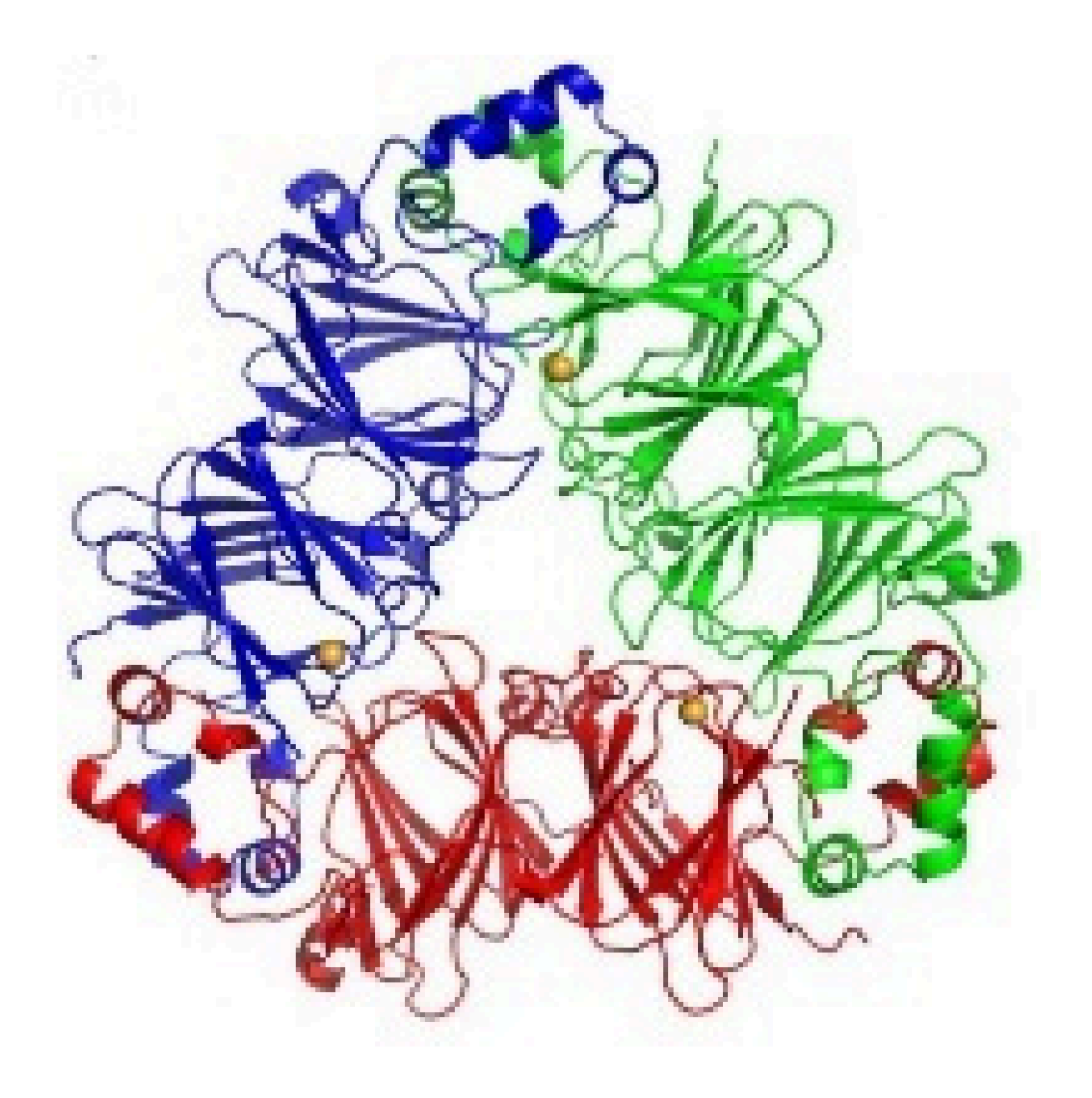	24%		AA271-280, AA336-345, AA522-541, AA610-637, AA684-693 and AA739-753
	Car i 4	Legumin seed storage protein; 11S globulin	Subunit of hexameric protein: 55.4 kDa	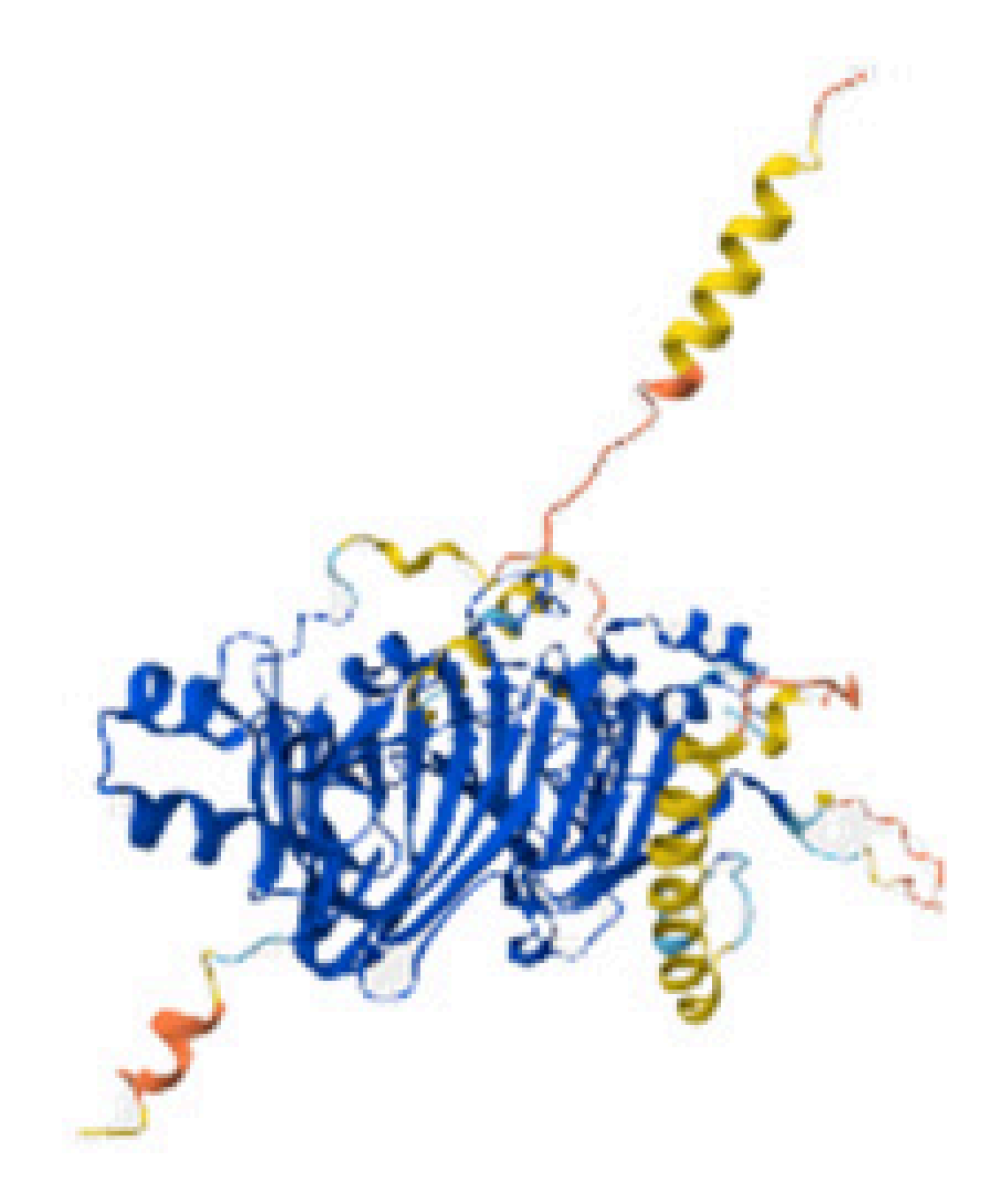	59%		AA28-39, AA88-99, AA118-129, AA121-132, AA124-135, AA127-138, AA130-141, AA133-144, AA202-213, AA205-216, AA208-219, AA232-243, AA235-246, AA238-249, AA304-315, AA382-393, AA421-432 and AA424-435
Pistachio	Pis v 1	2S albumin	7 kDa	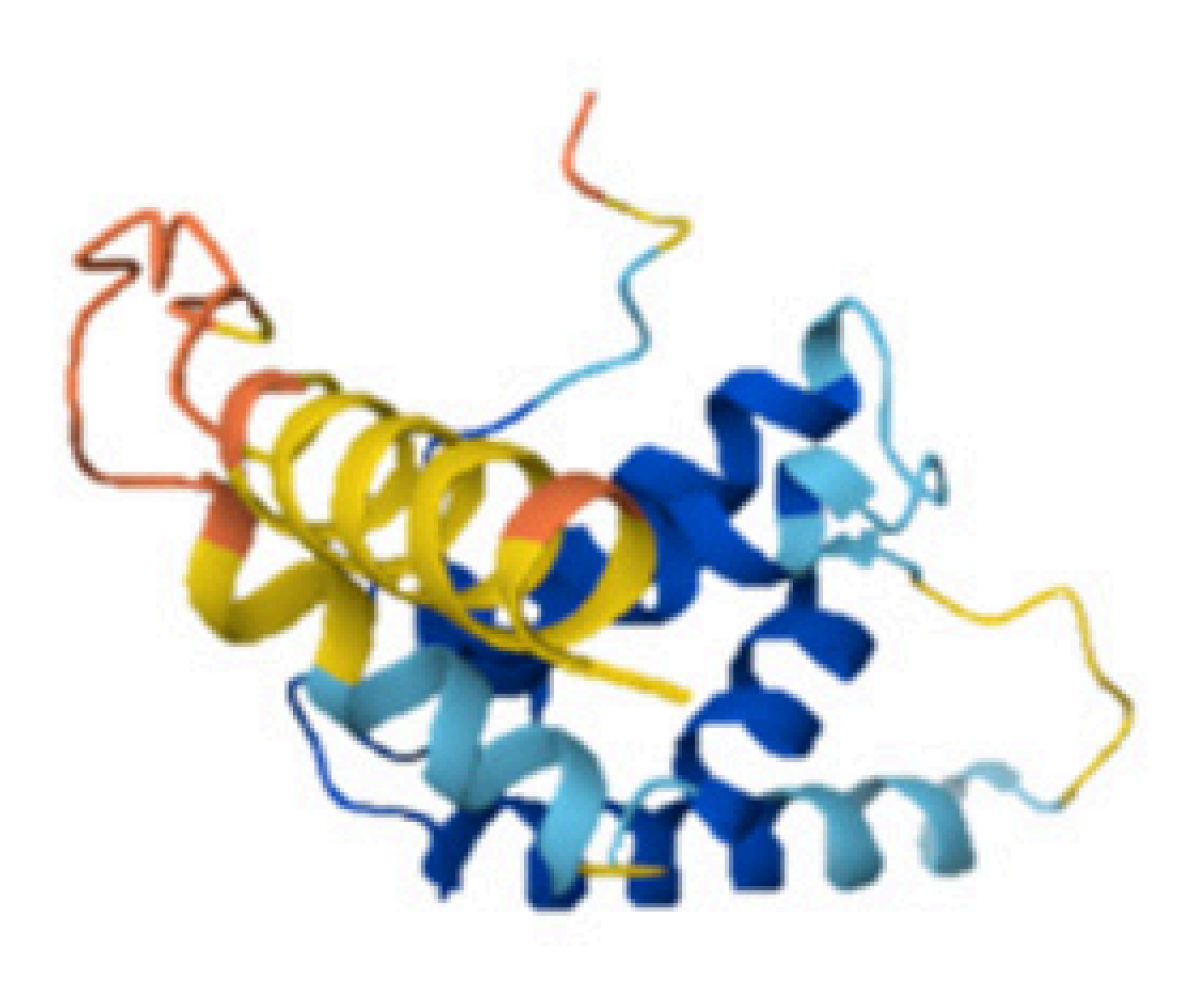	68%		AA17-37, AA41-58, AA65-88 and AA98-112
	Pis v 2	11S globulin subunit	32 kDa	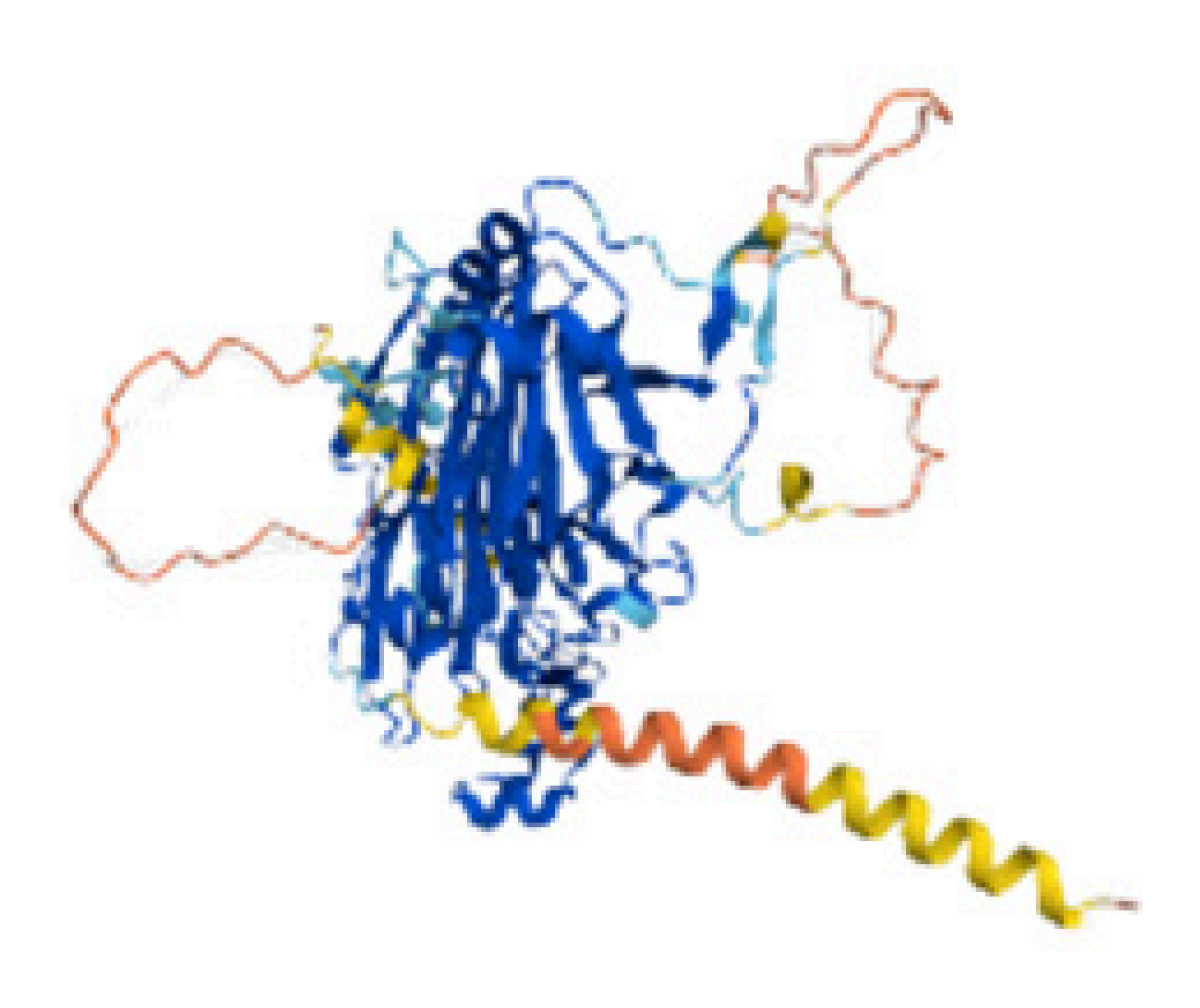	50%	Pis v 2.0101 Pis v 2.0201	
	Pis v 3	vicillin	55 kDa	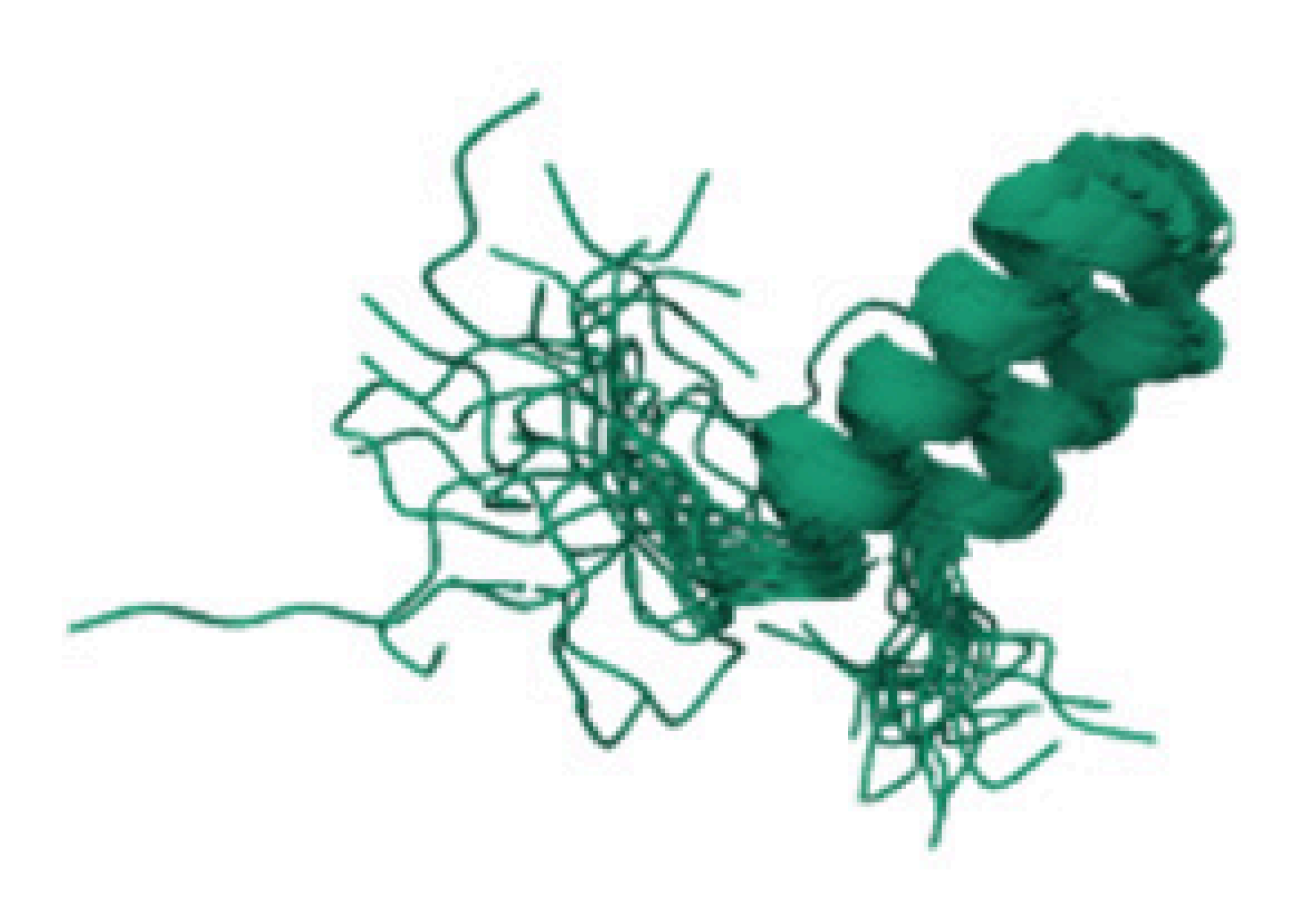	37%		AA258-276 and AA494-512
	Pis v 4	manganese superoxide dismutase	25.7 kDa	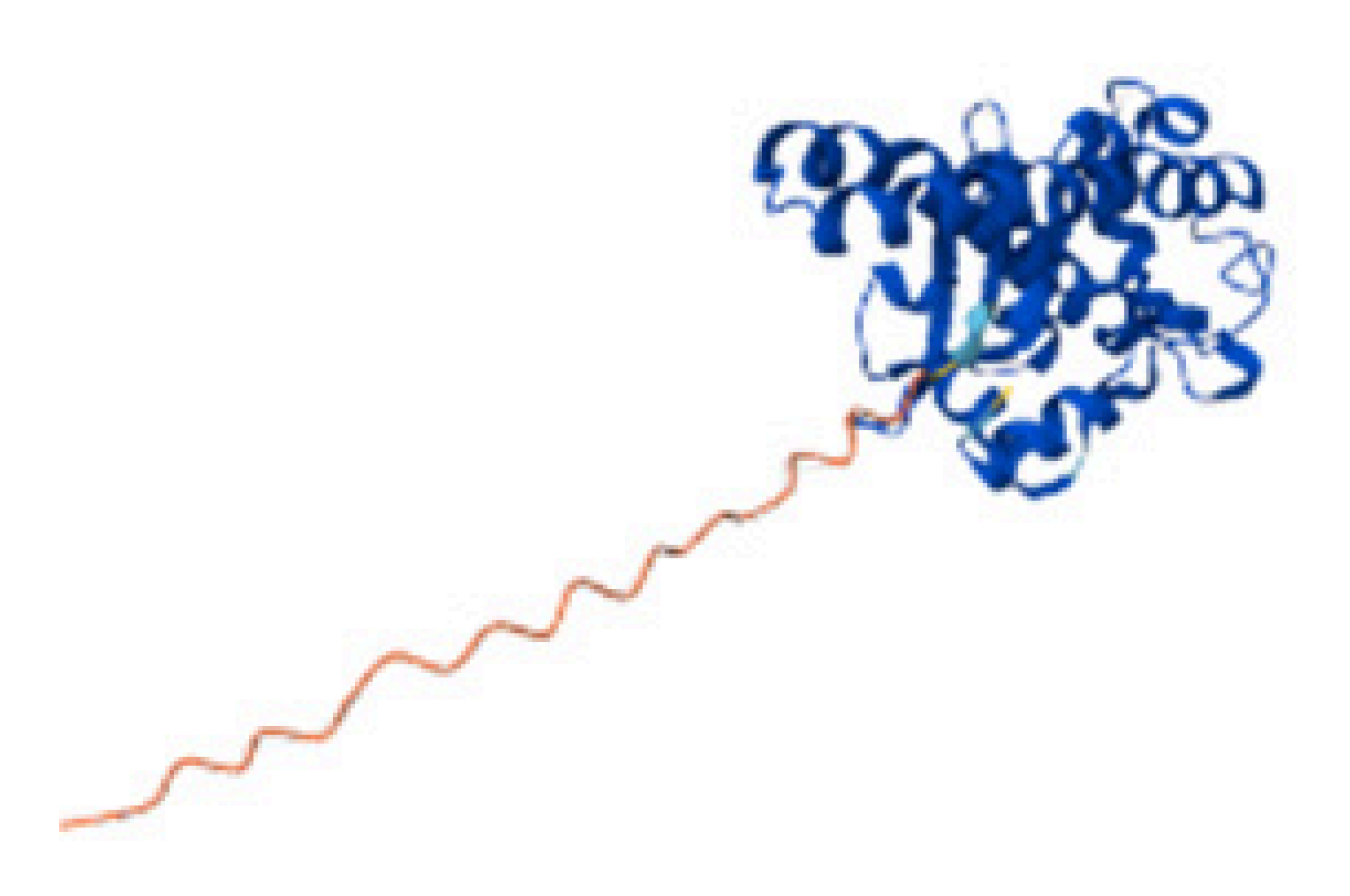	40%		
	Pis v 5	11S globulin subunit	36 kDa (acidic subunit)	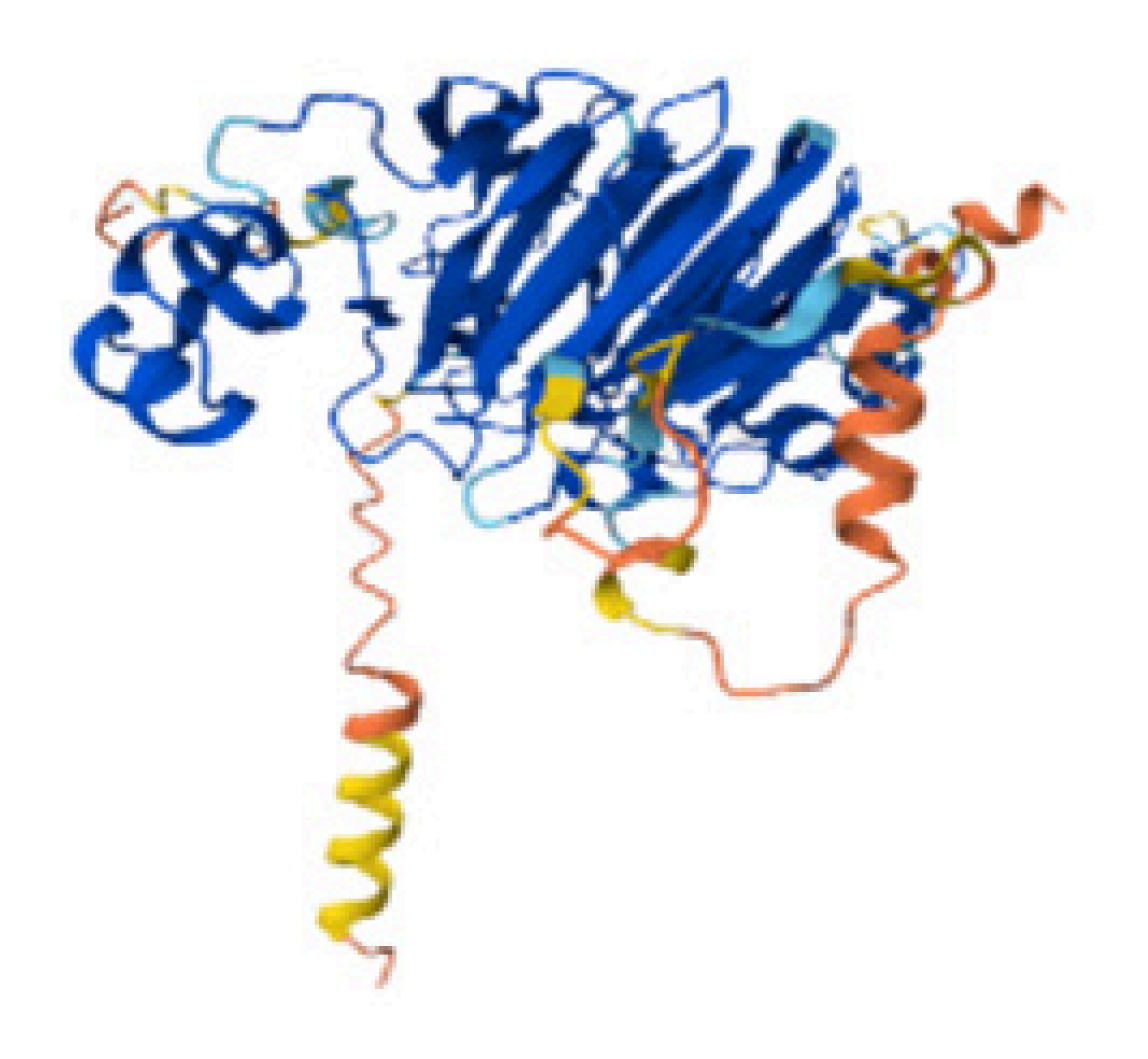	36%		AA251-269 and AA312-332
Brazil nut	Ber e 1	2S sulfur-rich seed storage albumin	9 kDa	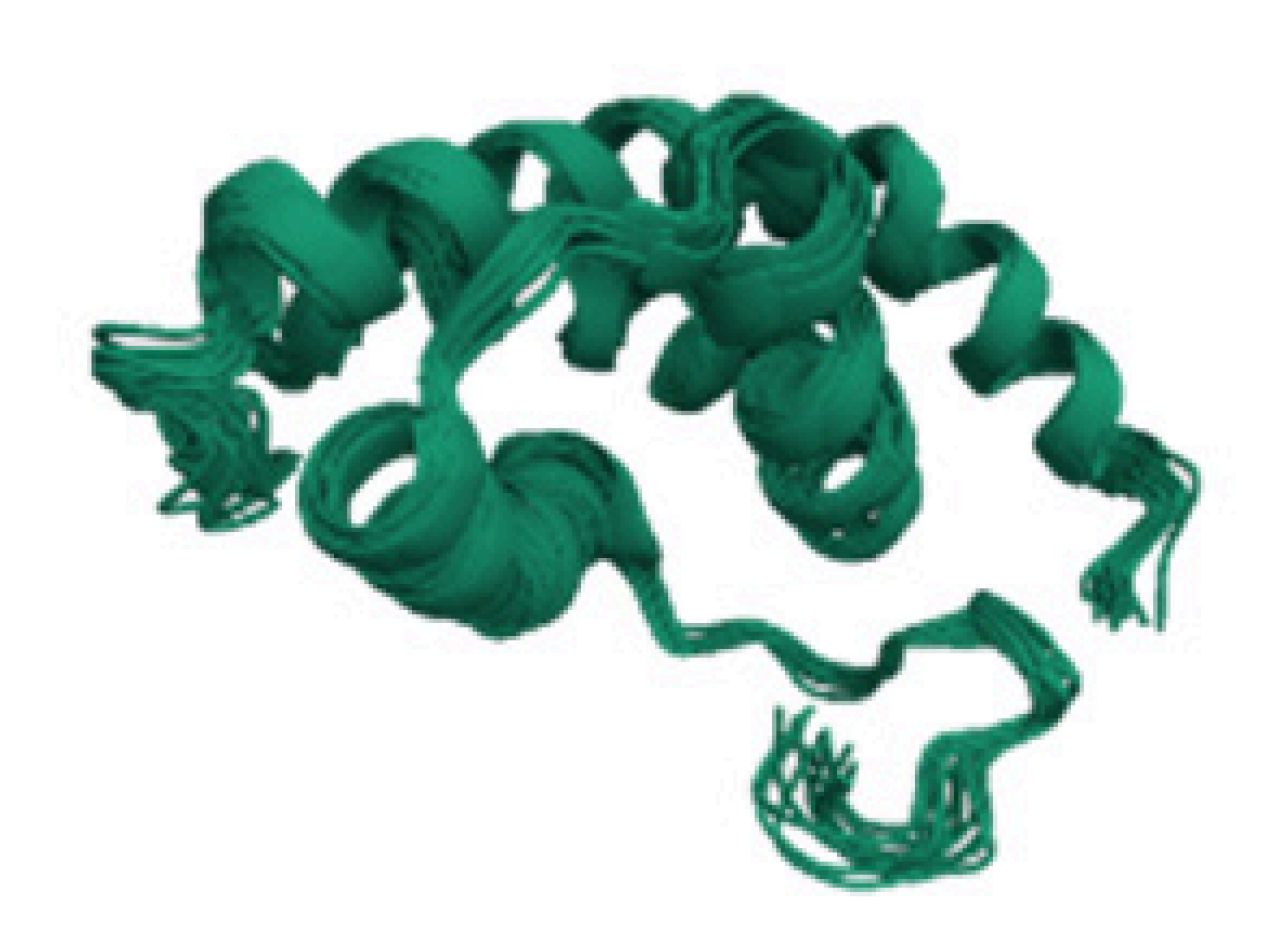	100%		AA7-20
	Ber e 2	11S globulin seed storage protein	29 kDa	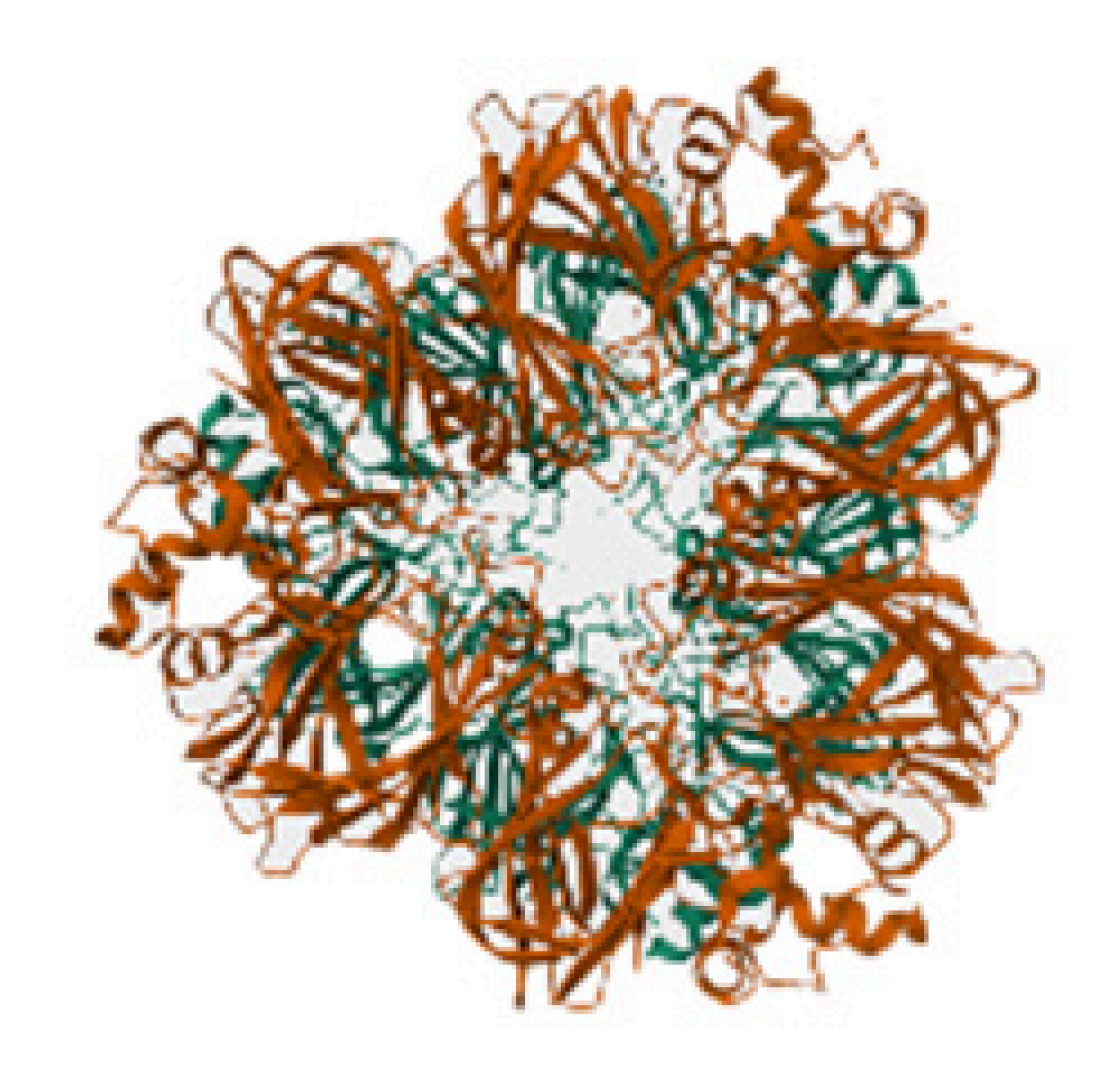	-		AA11-24 and AA60-73
Macadamia nuts	Mac i 1	Vicilin	50 kDa	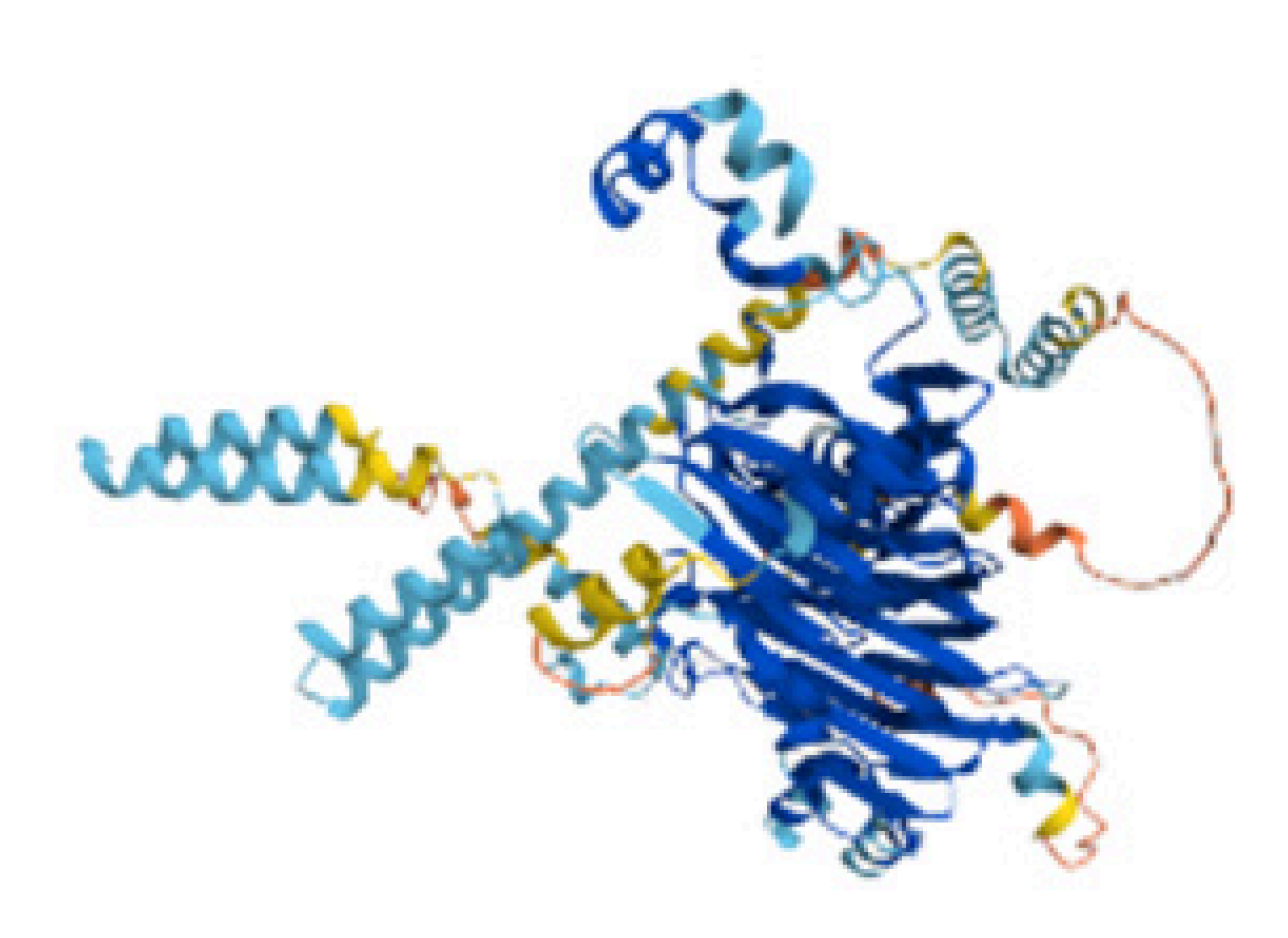	60%	Mac i 1.0101 Mac i 1.0101 (28–76)	
	Mac i 2	Legumin	60 kDa (non-reducing) 20 kDa and 40 kDa (reducing)	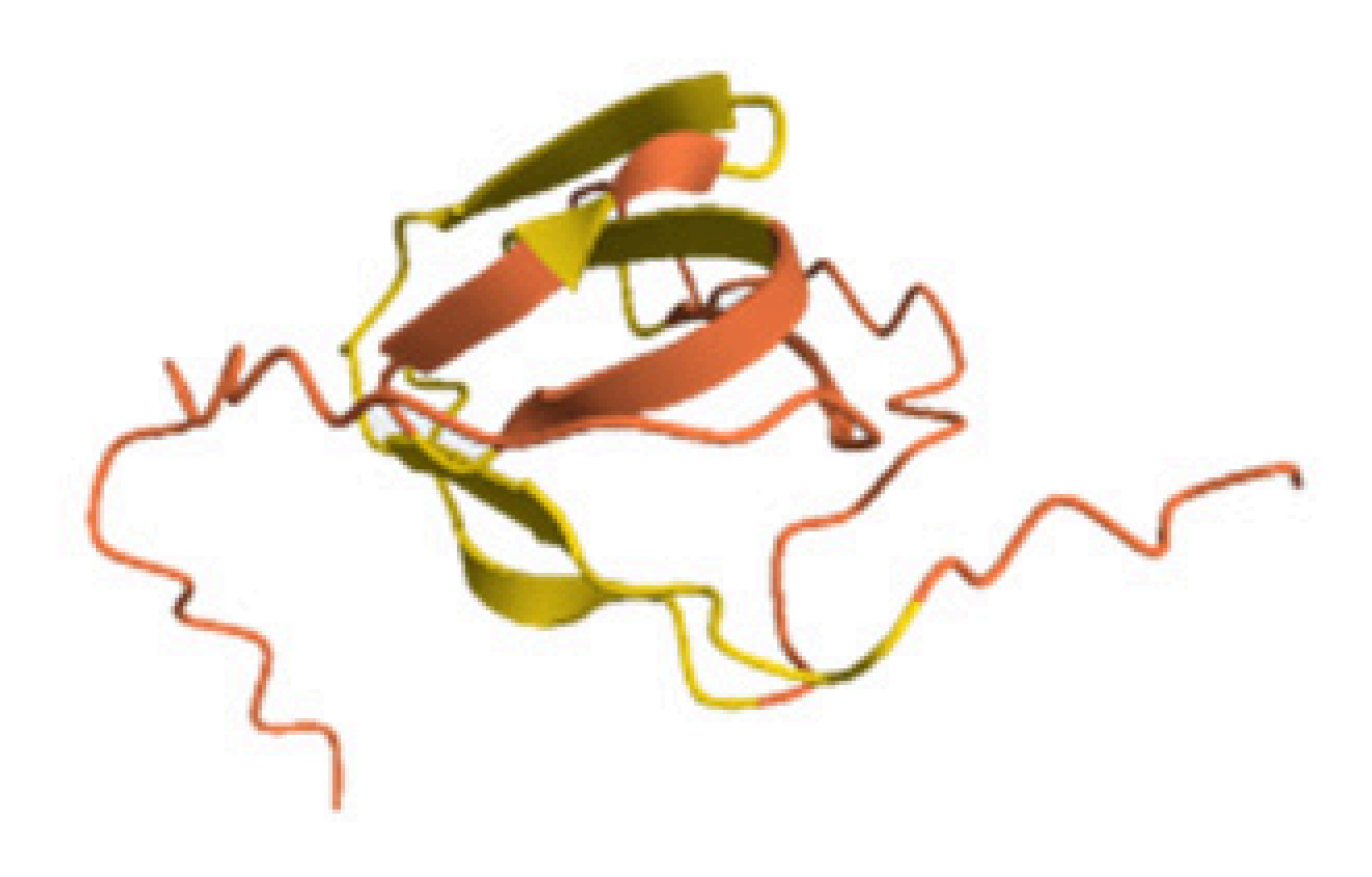	100%		
Chestnut	Cas s 1	Pathogenesis-related protein, PR-10, Bet v 1 family member	22 kDa	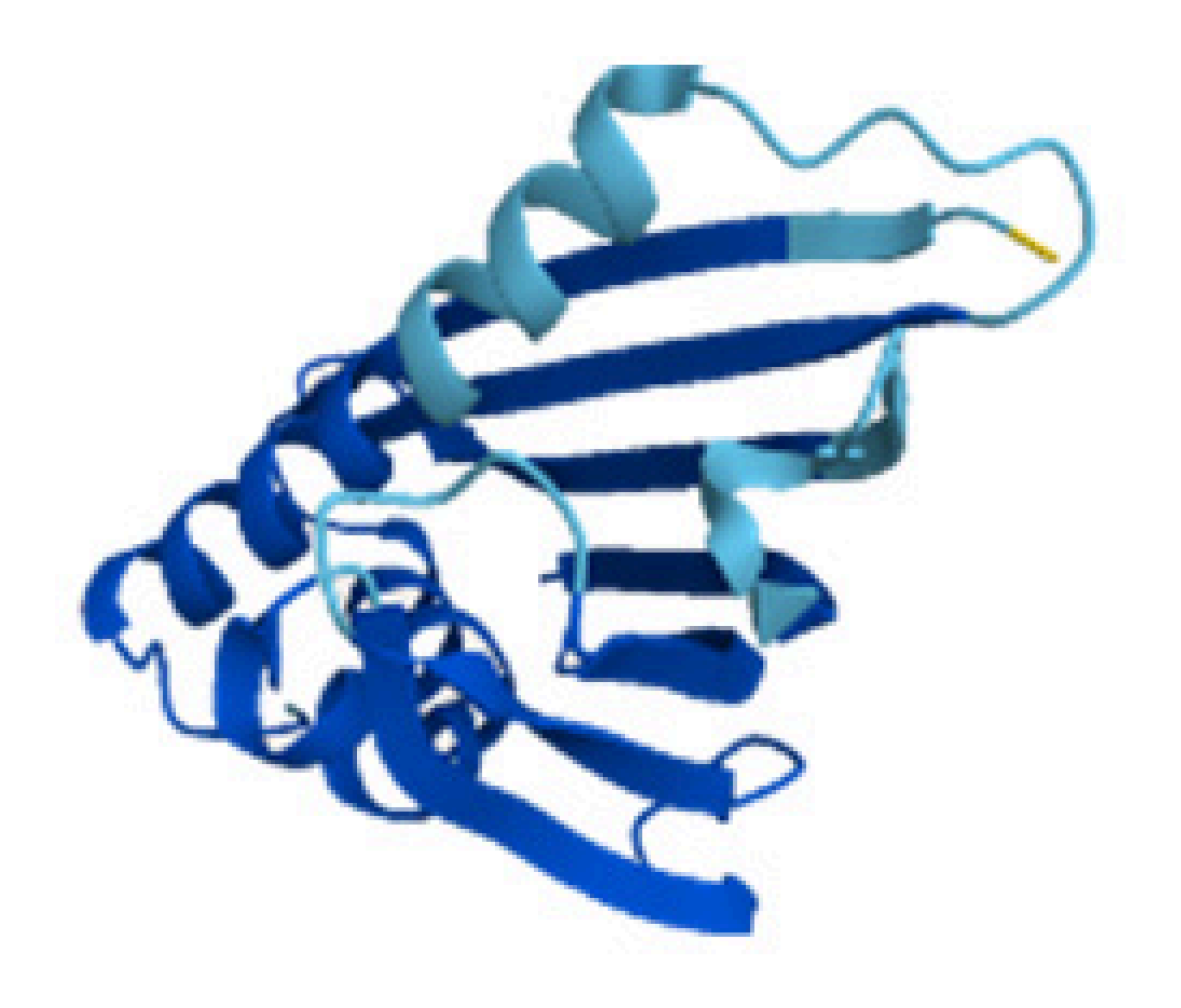	100%		
	Cas s 5	Chitinase class I	-	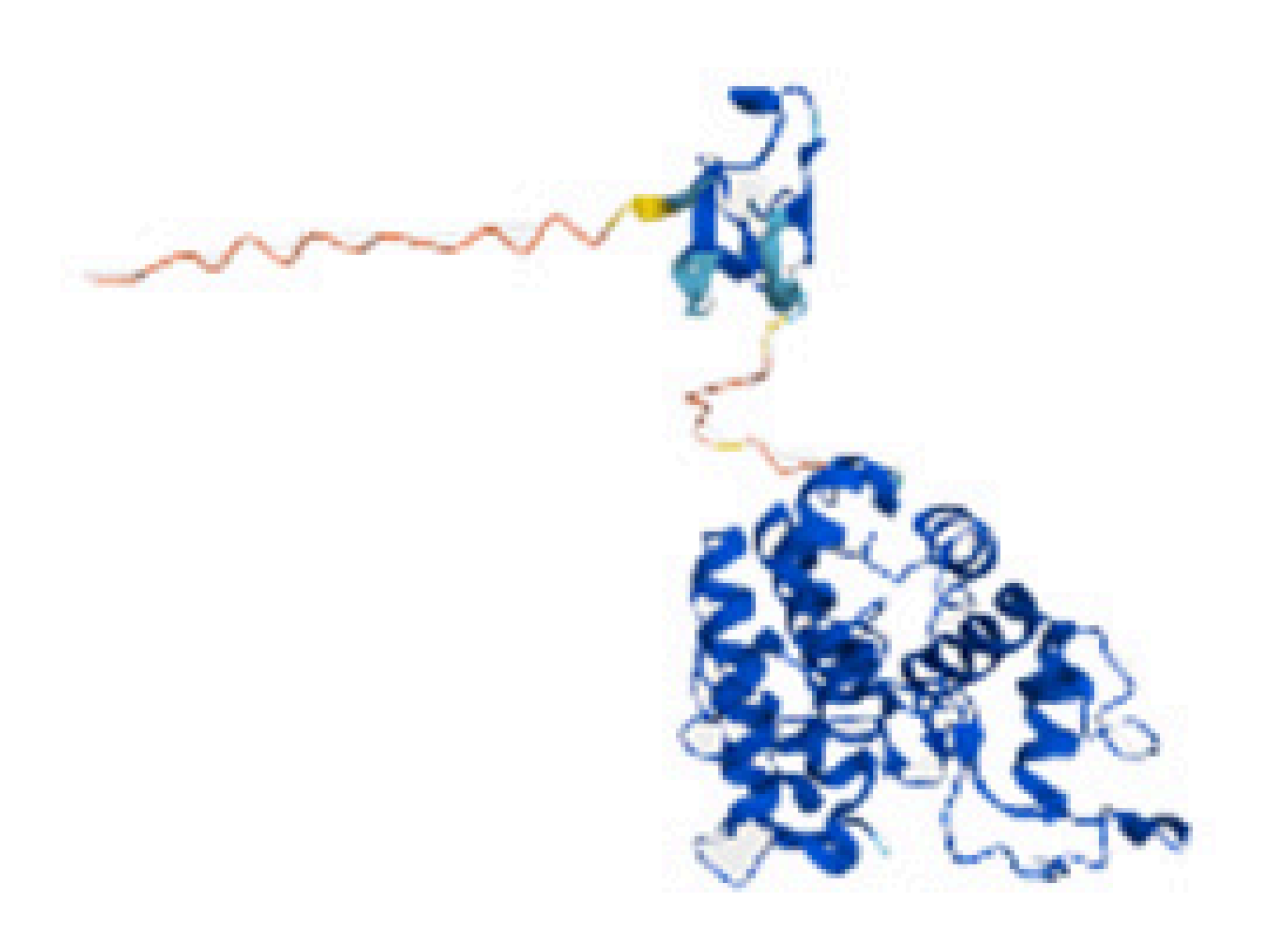	-		
	Cas s 8	Non-specific lipid transfer protein type 1 (nsLTP1)	12–13 kDa (non-red), 9 kDa (red)	-	66%		
	Cas s 9	Cytosolic class I small heat shock protein; Hsp20	17 kDa	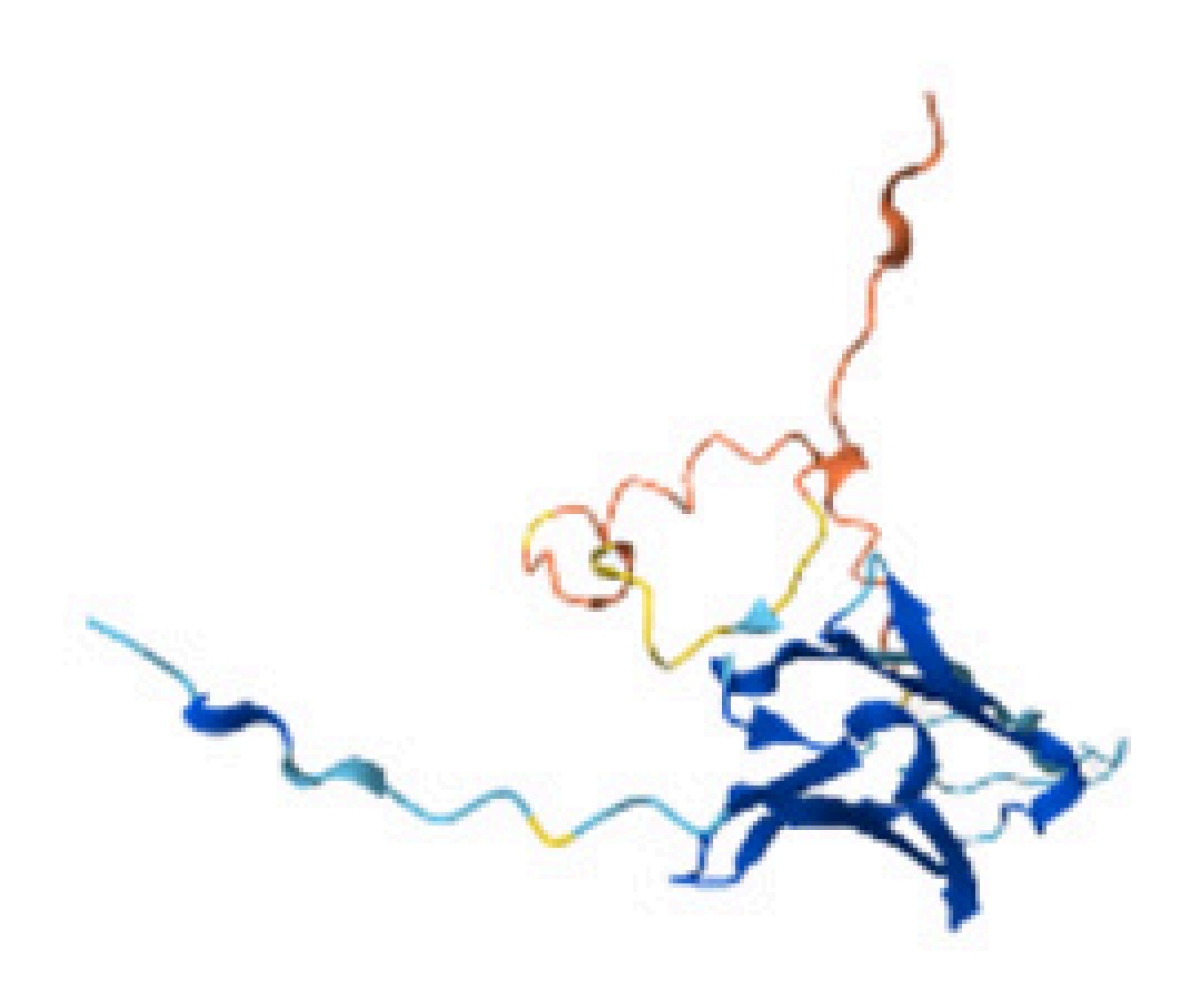	-		
*Pinus pinea* (Stone pine)	Pin p 1	2S albumin	6 kDa (reducing) and 15 kDa (non-reducing)	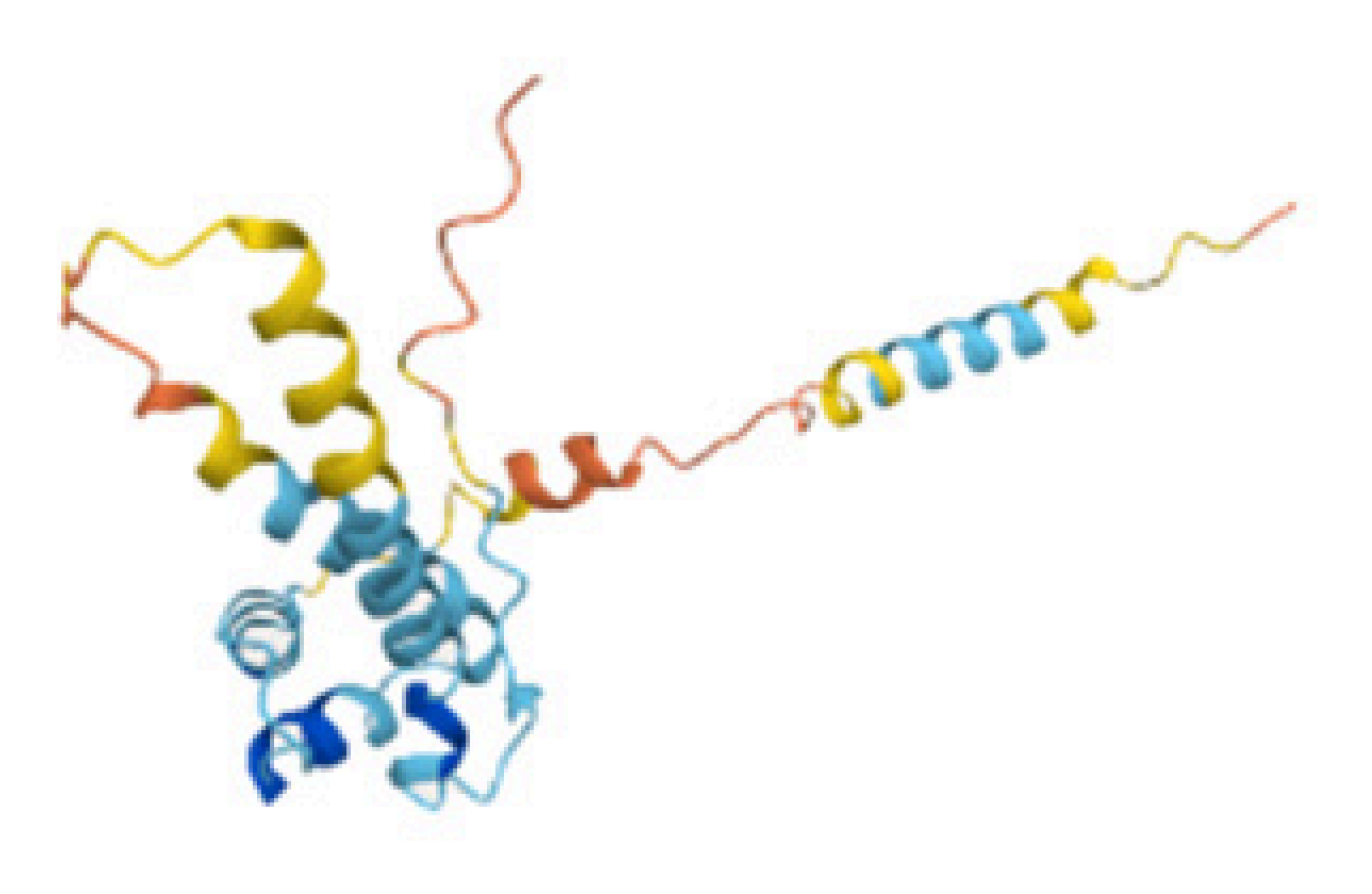	75%		AA61-68, AA69-75, AA112-118, AA136-142 and AA143-153
*Pinus koraiensis* (Korean pine)	Pin k 2	Vicilin	48 kDa	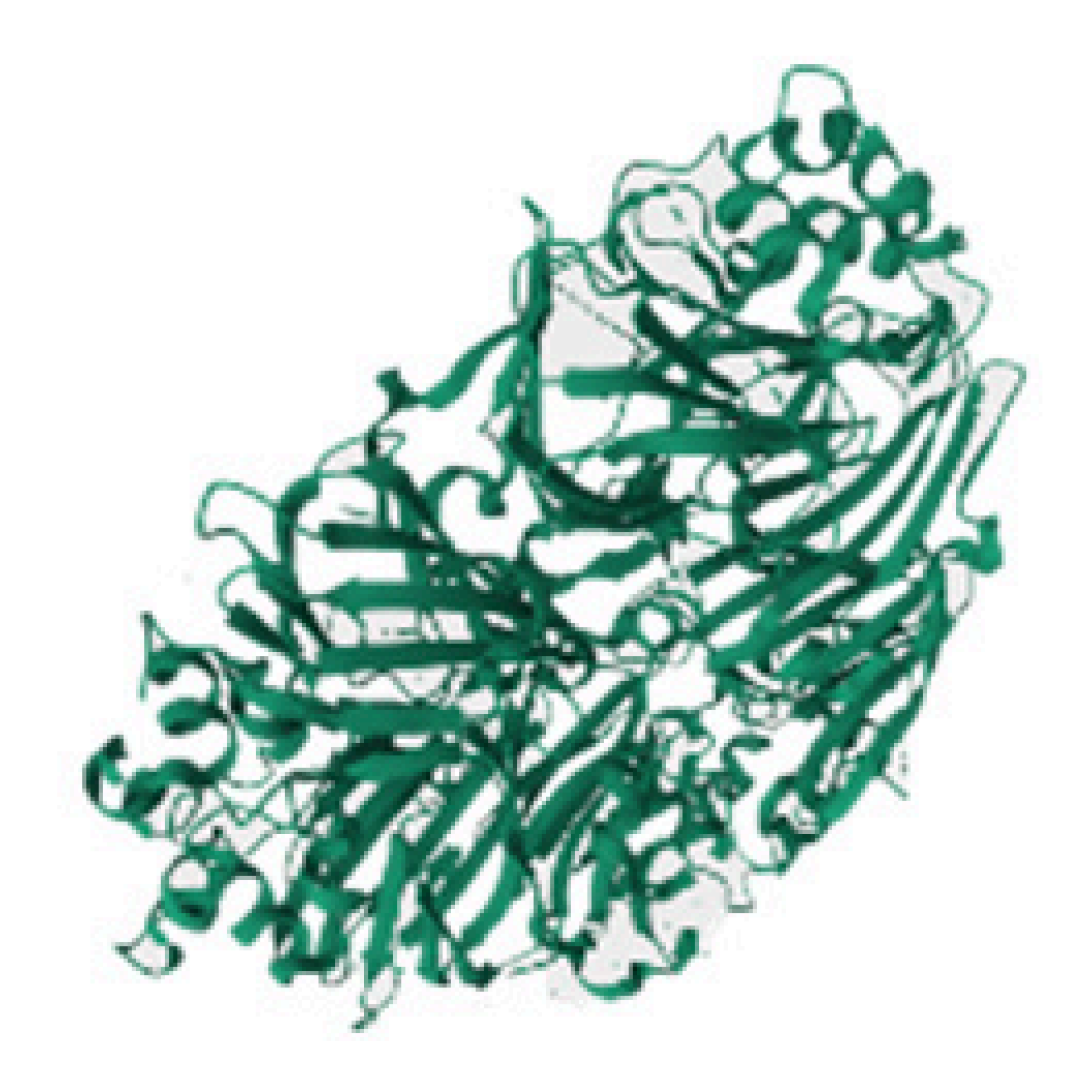	29%		
*Pinus yunnanensis* (*Yunnan pine*)	Pin y 2	Vicilin-like seed storage protein, 7S globulin	50 kDa	-	35%		

## Data Availability

No new data were created or analyzed in this study. Data sharing is not applicable.
